# Base-Labile Safety-Catch Linker: Synthesis and Applications in Solid-Phase Peptide Synthesis

**DOI:** 10.3390/ijms26052210

**Published:** 2025-02-28

**Authors:** Sikabwe Noki, Hossain Saneii, Beatriz G. de la Torre, Fernando Albericio

**Affiliations:** 1 Peptide Science Laboratory, School of Chemistry and Physics, University of KwaZulu-Natal, Westville, Durban 4000, South Africa; sikabwenoki7@gmail.com; 2 School of Laboratory Medicine and Medical Sciences, College of Health Sciences, University of KwaZulu-Natal, Durban 4041, South Africa; 3 AAPPTec, 6309 Shepherdsville Road, Louisville, KY 40228, USA; hsaneii@aapptec.com; 4 Department of Organic Chemistry, University of Barcelona, 08028 Barcelona, Spain

**Keywords:** diketopiperazine, peptide handle, polyfluoroalkyl substances, protecting group, solid-phase peptide synthesis

## Abstract

The safety-catch concept involves a protecting group that remains stable under a range of chemical conditions and subsequently becomes labile under one of those conditions upon a chemical modification of the protecting group. The safety-catch approach introduces flexibility into the scheme, enabling the use of the same reagent in two distinct steps of the chemical process. For example, it facilitates α-amino deprotection and final cleavage in a solid-phase peptide synthesis scheme. Herein, we developed a safety-catch linker based on sulfinyl designed to enable peptide elongation via fluorenylmethoxycarbonyl (Fmoc) chemistry. Subsequently, upon chemical modification (oxidation of the sulfinyl group into the corresponding sulfone), the peptide is released using a secondary amine via a β-elimination reaction, which also serves to remove the Fmoc group in each step. The optimization of both key reactions, oxidation of the linker, and peptide release were achieved using a multi-detachable system, which allows specific control of both reactions. The use of this linker opens the possibility of cleaving peptides from the solid support without trifluoroacetic acid.

## 1. Introduction

**Scheme 1:**
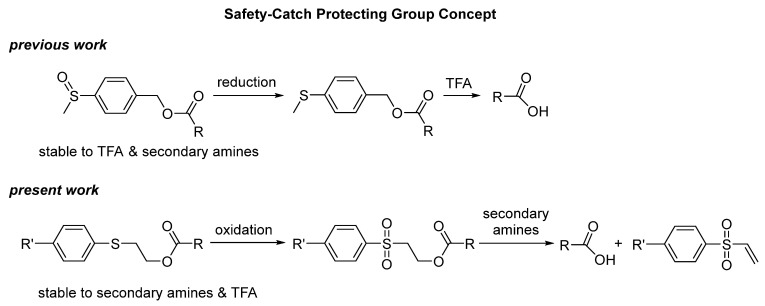
Previous and present work.

In the 1960s, R. Bruce Merrifield introduced the solid-phase concept into peptide synthesis [1]. The idea was simple: to use a solid (polymeric) protecting group for the *C*-terminal carboxylic group. Thus, elongation of the peptide chain is achieved by iterative coupling of protected amino acids (temporal protection for the α-amino function and permanent protection for the side chains of the trifunctional amino acids). This strategy allows for using excess reagents, as unreacted ones can be easily removed by filtration and washing of the solid support. The so-called solid-phase peptide synthesis (SPPS) approach has become the method of choice for preparing peptides for both research and industrial applications [2]. The development of SPPS techniques has had bidirectional effects. On the one hand, it has enabled the multi-kilogram production of linear peptides comprising more than 30 residues using only two types of protecting groups, namely those that are labile in response to a base [fluorenylmethoxycarbonyl (Fmoc)] and those labile in response to an acid [*tert-*butyl (*t*Bu) or similar] [3]. However, it has also facilitated the synthesis and subsequent biological investigation of more complex peptides, including those with cyclic or branched structures and/or labile amino acids, as a first step towards bringing these peptides to market [4]. These advancements have been fueled by developing novel SPPS approaches, including new solid supports [5,6], coupling reagents [7], and protecting groups [8]. Among the latter, it has been proposed that they can be removed with Pd (0) (allyl), with mild reducing agents (*p*-nitrobenzyl), or by photolysis (*o*-nitrobenzyl) [8]. However, none of these protecting groups show the ease of manipulation associated with treatments with weak acids or bases. The ideal protecting group is stable in response to both acid and base treatment but then becomes labile in the presence of either acid or base after a chemical manipulation. This is known as a *safety-catch* protecting group [9,10,11]. In recent years, our group and others have proposed the 4-(methylsulfinyl) benzyl (Msib)/4-(methylthio)benzyl (Mtb) pair as a useful example of this concept [12,13]. Msib and related sulfinyl protecting groups are stable in acids such as trifluoracetic acid (TFA) and also in bases. However, at the end of the synthetic process, a convenient reduction to thio derivatives makes the protecting group labile in response to TFA. The key to this process is the reduction of methylsulfoxide (electron-withdrawing group that destabilizes the benzyl carbocation) to methylthio (electron-donating group that stabilizes the benzyl carbocation). Currently, we are advocating a complementary *safety-catch* protecting group also based on a S moiety. In this case, the group is stable in response to bases such as piperidine and to other secondary amines and acids, and after peptide elongation, it becomes labile in the presence of secondary amines. Thus, 2-(phenylthio)ethyl derivatives are stable in response to secondary amines and acid treatments. However, after oxidation to the corresponding sulfones, they can undergo a β-elimination reaction, similar to fluorenylmethyl (Fm), with the release of the functional group (Scheme 1).

The oxidized form, the sulfinylethyloxy moiety, was developed by Tesser and co-workers as a protecting group in the form of a carbamate of the α-amino function [14,15,16]. This protecting group has similar properties to the Fmoc group developed by Carpino a few years earlier [17], which is stable in response to acids and labile in the presence of secondary amines through a β-elimination reaction. Our group has also developed a nitrophenyl variant for the α-amino function [18]. Later, a similar oxidized moiety was used as a linker in the *tert-*butyloxycarbonyl (Boc) strategy [19]. 

**Figure 1:**
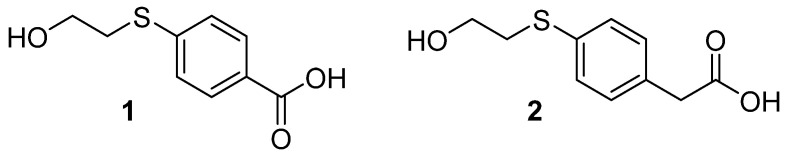
Structures of 4-((2-hydroxyethyl) thio) benzoic acid (**1**) and 2-(4-((2-hydroxyethyl)thio)phenyl)acetic acid (**2**) linkers.

Herein, we propose two thio linkers for SPPS, namely 4-((2-hydroxyethyl) thio) benzoic acid (**1**) and 2-(4-((2-hydroxyethyl) thio) phenyl) acetic acid (**2**) (Figure 1). The *C*-terminal protected amino acid and the linker form an ester bond that is stable in response to bases such as secondary amines and to acids such as TFA and is therefore compatible with Fmoc and Boc chemistry. The use of the two α-amino protecting groups in the same synthesis can have advantages in minimizing side reactions. After peptide elongation, oxidation of the thioether to the sulfone will render an ester bond labile in response to secondary amines through a β-elimination reaction. Similar versions of these linkers were used by Garcia-Echeverria [20], Wade et al. [21], and Tumelty et al. [22] for the solid-phase synthesis (SPS) of small molecules. Furthermore, in the same context of supported organic synthesis, the oxidized (sulfone) derivatives of similar linkers, which do not follow the safety-catch strategy, have been described by the groups led by Rutjes and Hiemstra [23,24].

## 2. Results and Discussion

### 2.1. Linker Syntheses

**Scheme 2:**
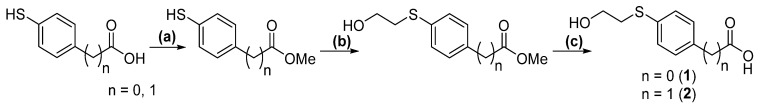
Synthesis of linkers **1** and **2**. Reagents: (**a**) MeOH, H_2_SO_4,_ 70 °C, 2 h; (**b**) 2-bromoethanol, Cs_2_CO_3_, DMF, RT, overnight; and (**c**) LiOH, THF-H_2_O (1:1), RT, overnight. See SI for a detailed experimental part and Figures S1–S11 for characterization spectra.

First, two versions of the linker were prepared starting from two commercially available mercapto-phenyl acid derivatives, namely 4-mercaptobenzoic acid and 4-mercaptophenylacetic acid, using straightforward synthesis (Scheme 2). Both linkers were obtained with an overall yield of 90% (see the Supporting Information (SI) for the full experimental protocol and characterization). These linkers can be incorporated into any amino resin using standard coupling conditions (see below). These resins should allow for the elongation of the peptide chain using either Fmoc, Boc, or even other α-amino protecting groups such as allyloxycarbonyl (Alloc) [25] or *p*-nitrobenzyloxycarbonyl (pNz) [26], due to the high stability of the ester bond formed between the first amino acid and linkers (**1**, **2**). 

The performance of the two linkers was very similar, and although we initially worked with both, we decided to continue with linker **1** [from here, 4-((2-hydroxy**E**thyl) **Thio**)**B**enzoic acid (ETB linker)] because the starting product was much more affordable.

### 2.2. ETB Linker Activation [Oxidation of Sulfide (Thio) to Sulfone] 

**Scheme 3:**
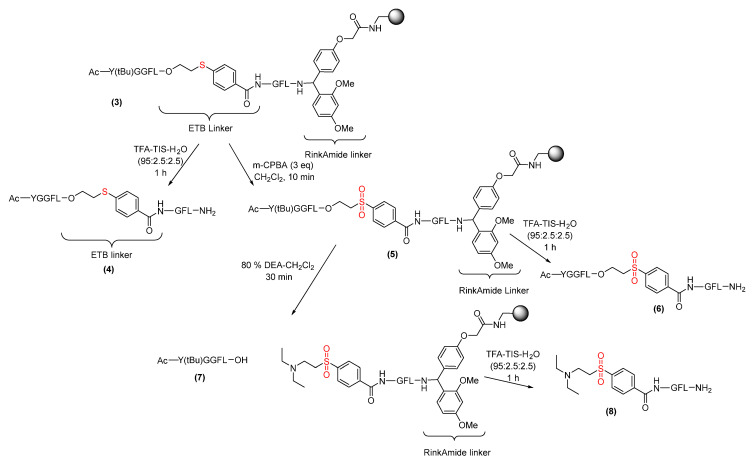
Study of the activation and cleavage of ETB resin.

We studied the linker activation in SPPS using a multi-detachable (double) linker strategy [27], which used a Rink Amide linker in combination with the ETB linker (**1**). The peptide linker (**3**) constructs shown in Scheme 3 were employed to study the activation process, involving the oxidation of sulfide (thio) to sulfone, for the ETB linker (**1**). Initially, a tripeptide, Gly-Phe-Leu, was elongated on Rink Amide resin using Fmoc chemistry to facilitate the detection of compound (**8**) by LC-MS. Then, the ETB linker (**1**) was introduced, and the full sequence of Leu-enkephalin (Tyr-Gly-Gly-Phe-Leu) was extended from the ETB linker, with acetylation of the α-amino terminal of Tyr using Fmoc/*t*Bu chemistry once again.

Elongation of the two peptides was achieved using *N*,*N*’-diisopropylcarbodiimide (DIC)–Oxyma Pure as a coupling mixture, which is one of the most potent acylation methods [28]. Conversely, the ETB linker was introduced using DIC in the presence of 1-hydroxybenzotriazole (HOBt). Although less effective than Oxyma Pure, this approach helps prevent double acylation of the ETB linker, and it was carried out for 1 h in DMF. Similar to other hydroxymethyl resins, the incorporation of the first protected amino acid into the ETB linker was facilitated by using DIC in the presence of *N*,*N*-dimethylaminopyridine (DMAP) as a catalyst. This reaction took place for 2 h in DMF (see SI, Scheme S1, for details).

**Figure 2:**
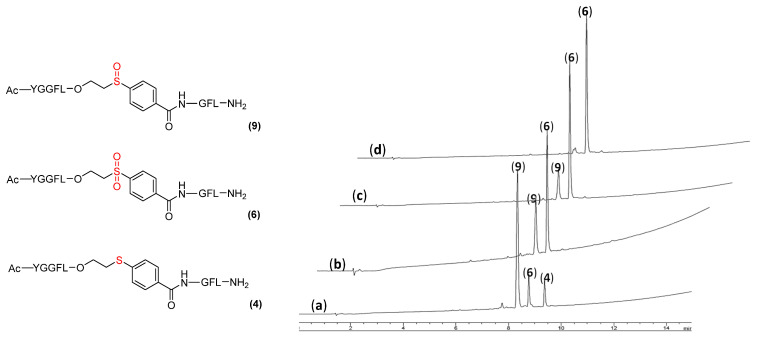
Study of ETB linker oxidation with *m*-CPBA: Conditions—all Cleavage-TFA/TIPS/H_2_O (95/2.5/2,5, v/v/v) 1 h. Gradient: 5/95 (v/v) of MeCN+0.1% TFA/H_2_O+0.1% TFA, (**a**) *m*-CPBA (1 Eq.) for 10 min, (**b**) *m*-CPBA (2 Eq.) for 10 min, (**c**) *m*-CPBA (2 Eq.) for 30 min, and (**d**) m-CPBA (3 Eq.) for 10 min. See Figures S12–S15 for characterization.

Using Ac-Tyr(*t*Bu)-Gly-Gly-Phe-Leu-O-ETB-linker-Gly-Phe-Leu-NH-Rink Amide resin (**3**) as a substrate, we studied the oxidation of the sulfide to sulfone using *meta*-chloroperoxybenzoic acid (*m*-CPBA) in DCM. Various amounts of *m*-CPBA (1–3 Eq.) and reaction times (10–30 min) were tested. After activation, the peptide resin was subjected to a TFA treatment for further High-performance liquid chromatograph (HPLC) analysis of the oxidation step. In cases where oxidation did not occur, the reduced form (**4**) was produced. The presence of the sulfone derivative (**6**) indicated full oxidation, whereas the presence of the product (**9**) indicated partial oxidation, leading to the corresponding sulfoxide (Figure 2). 

First, a non-excess of *m*-CPBA (1 Eq.) was used to identify the corresponding sulfoxide (partial oxidation). Figure 2a shows a major peak, indicating the incorporation of a single oxygen atom (sulfoxide) (**9**), accompanied by roughly equivalent amounts of the double oxidation product (sulfone) (**6**) and the initial starting material (sulfide) (**4**). This result implies the complete consumption of all *m*-CPBA within 10 min. Subsequently, 2 Eq. of *m*-CPBA was used, and after 10 min, the main peak corresponded to the sulfone derivative (**6**), with the sulfoxide derivative (**9**) accounting for approximately half the total Figure 2b. By the 30 min mark Figure 2c, the peak corresponding to the targeted sulfone derivative (**6**) showed a slight increase, while the presence of the sulfoxide derivative (**9**) remained notable (approx. 25%). Finally, almost quantitative conversion was achieved using 3 Eq. (1.5 excess) of *m*-CPBA within 30 min Figure 2d. In summary, the solid-phase oxidation of the sulfide to sulfone using moderate excesses of *m*-CPBA proceeded smoothly, without affecting the peptide chain and within a relatively short timeframe (30 min). 

**Figure 3:**
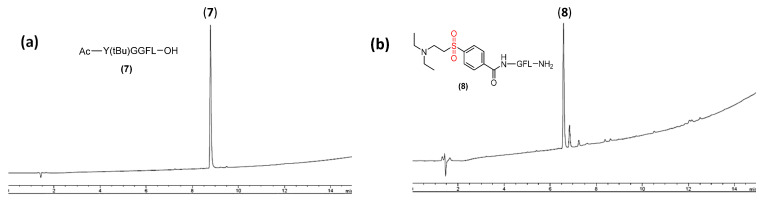
(**a**) Direct cleavage of the peptide from the resin with 80% DEA-DCM; (**b**) cleavage of the remaining peptide resin with TFA after cleavage with 80% DEA-DCM. Gradient: 5/95 (v/v) of MeCN+0.1% TFA/H_2_O+0.1% TFA.

To confirm our hypothesis, the protected Ac-Leu-enkephalin peptide [Ac-Tyr(*t*Bu)-Gly-Gy-Phe-Leu-OH (**7**)] was cleaved from the resin using 80% Diethylamine (DEA) in DCM. The HPLC chromatogram Figure 3a showed the target peptide with excellent purity. Furthermore, the remaining peptide-oxidized resin was treated with TFA in the presence of scavengers, and the HPLC chromatogram clearly showed the formation of the DEA adduct (Figure 3b). Peaks that appeared after the main peak (**8**) between 6.7 and 7.1 min did not correspond to the non-oxidized compounds (**4**) and (**9**), which appeared between 8.4 and 9.5 min (Figure 2). If the oxidation had not been complete, the peptide (**7**) would not have been cleaved and would have appeared as part of the moiety cleaved with TFA in Figure 3b.

### 2.3. Peptide Cleavage from Activated ETB Linker

**Scheme 4:**
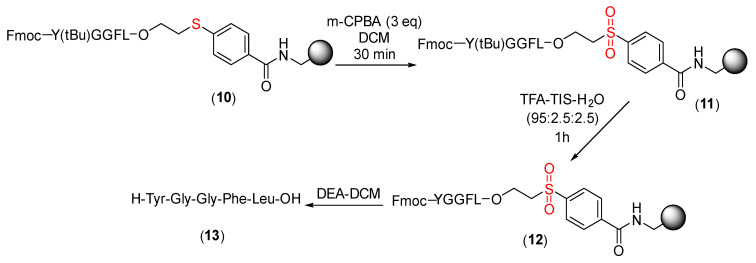
Study of the cleavage of the peptide from ETB resin using DEA in DCM.

Having addressed the activation/oxidation step, we studied the cleavage. As a first screening, the Fmoc/*t*Bu protected peptide ETB resin (**10**) was oxidized and treated with TFA to remove the *t*Bu of Tyr. The peptide resin was subjected to three cleavages with DEA (100%, 80%, and 50%) for 20 min (Scheme 4). First, DEA was selected over piperidine as the amine of choice for Fmoc removal, along with other secondary amines, due to its lower boiling point (55.6 °C) compared to piperidine (106 °C) and morpholine (129 °C). Furthermore, it is important to note that DEA is not subjected to the same regulatory restrictions as piperidine [29]. The outcomes presented in Table 1 indicate that even a 50% concentration of DEA in DCM resulted in excellent peptide detachment within 20 min.

**Figure 4:**
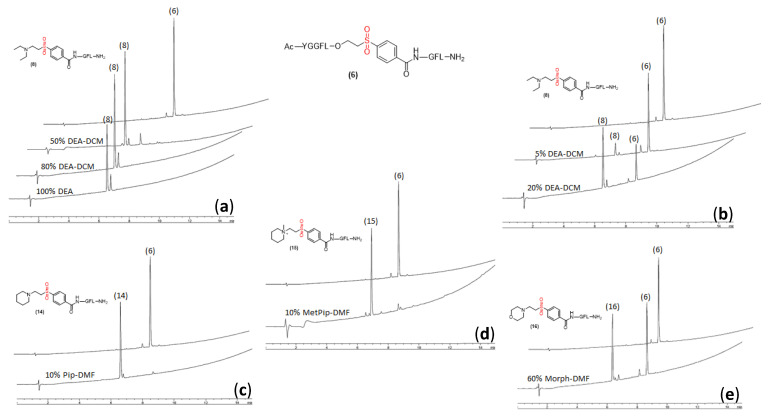
HPLC chromatogram for cleavage study using various basic conditions. Gradient: 5/95 (v/v) of MeCN+0.1% TFA/H_2_O+0.1% TFA. (**a**) Cleavage condition 100% DEA, 80% and 50% DEA-DCM in 30 min; (**b**) 20% and 5% DEA-DCM in 30 min; (**c**) 10% piperidine–DMF in 30 min; (**d**) 10% 4-methylpiperidine–DMF in 30 min; and (**e**) 60% morpholine–DMF in 30 min. See Scheme S3 and Figures S18–S27 for the HPLC of the cleaved peptide Ac-Y(*t*Bu)GGFL-OH and the rest of the peptides addressed in this study.

Next, the cleavage was studied in greater detail under various basic conditions, again using the multi-detachable linker system shown in Scheme 3. Thus, the peptide resin (**5**) was treated with a base for 30 min, resulting in Ac-Y(*t*Bu)GGFL-OH (**7**). The remaining peptide-oxidized resin was treated with TFA in the presence of scavengers. If the cleavage with bases is not successful, the ETB-containing linker moiety (**6**) will be obtained. If the cleavage occurs, the adduct of the base with vinylsufone derivative (**8**) will be obtained. The **8:6** ratio will render the yield of the basic cleavage. First, the conditions shown in Table 1 were repeated. Thus, the peptide resin (**5**) was treated with 100% DEA, 80% DEA-DCM, and 50% DEA-DCM for 30 min. The HPLC chromatogram Figure 4a demonstrated complete cleavage of the peptide using these three basic conditions, with no detectable presence of peptide (**6**).

The peptide resin (**5**) was then treated with 20% DEA-DCM and 5% DEA-DCM for 30 min, yielding peptide (**7**). The remaining peptide-oxidized resin was again treated with TFA and scavengers. In the case of treatment with 20% DEA-DCM, only 63% of the peptide was cleaved, while 37% [Ac-YGGFL-O-ETB-Linker-GFL-NH2] (**6**) remained uncleaved. For the treatment with 5% DEA-DCM, only 15% of the peptide was cleaved, with 85% (**6**) remaining uncleaved Figure 4b.

Furthermore, the protected peptide resin (**5**) was treated with 10% piperidine, 10% 4-methylpiperidine, and 60% morpholine in DMF for 30 min, resulting in peptide (**7**). The remaining peptide oxidized and bound to the resin was treated with TFA in the presence of scavengers. The HPLC chromatogram revealed that when treated with 10% piperidine, 96% of the peptide was cleaved, while only 4% (**6**) remained uncleaved Figure 4c. When treated with 10% 4-methylpiperidine, 93% of the peptide was cleaved while 7% (**6**) remained uncleaved (Figure 4d). Finally, when treated with 60% morpholine in DMF, only 47% of the peptide was cleaved, with 53% of peptide (**6**) remaining uncleaved (Figure 4e). (See Table 2 for cleavage study) These results are consistent with the proven efficiency of these three cyclic secondary amines at removing Fmoc in SPPS [30], where piperidine is slightly more efficient than 4-methylpiperidine and much more efficient than morpholine.

### 2.4. Stability of Peptides with Sensitive Amino Acids (His, Trp, and Cys) in m-CPBA Oxidizing Reagent

**Figure 5:**
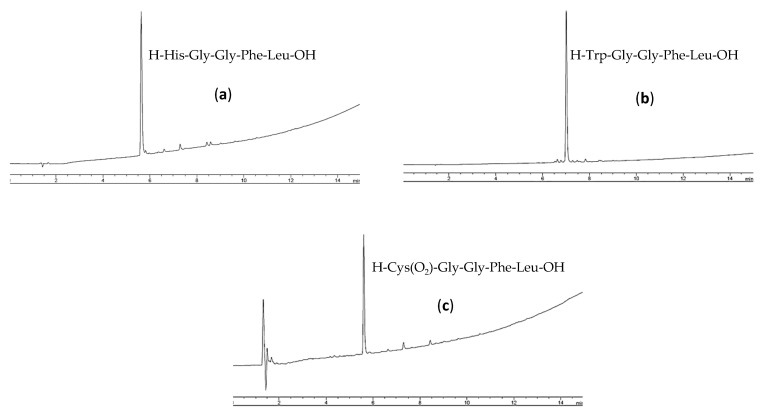
HPLC chromatograms of Leu-enkephalin peptides containing His, Trp, and Cys. Gradient: 5/95 (v/v) of MeCN+0.1% TFA/H2O+0.1% TFA. See Schemes S4–S6 and Figures S28–S33 for LC-MS characterization.

This strategy is not compatible with Met-containing peptides because the oxidizing conditions will also render the corresponding sulfone of Met, which cannot be reduced back to the sulfide. To test the compatibility of our strategy with other peptides containing sensitive amino acids, protected Leu-enkephalin peptides containing His(Trt), Trp(Boc), and Cys(Trt) were synthesized following standard Fmoc chemistry. For peptides containing His and Trp, treatment with 3 Eq. of *m*-CPBA in DCM for 10 min rendered peptides of acceptable purity or higher Figure 5a,b. On the other hand, the Cys peptide, even when protected with the hindered trityl (Trt) group, was cleanly converted to the sulfinic acid derivative, as shown by its m/z (528.22, which represents a mass increase of +32, from the expected m/z of 496.22) (Figure 5c).

### 2.5. Compatibility of ETB Resin with Boc Chemistry to Minimize DKP Formation 

**Figure 6:**
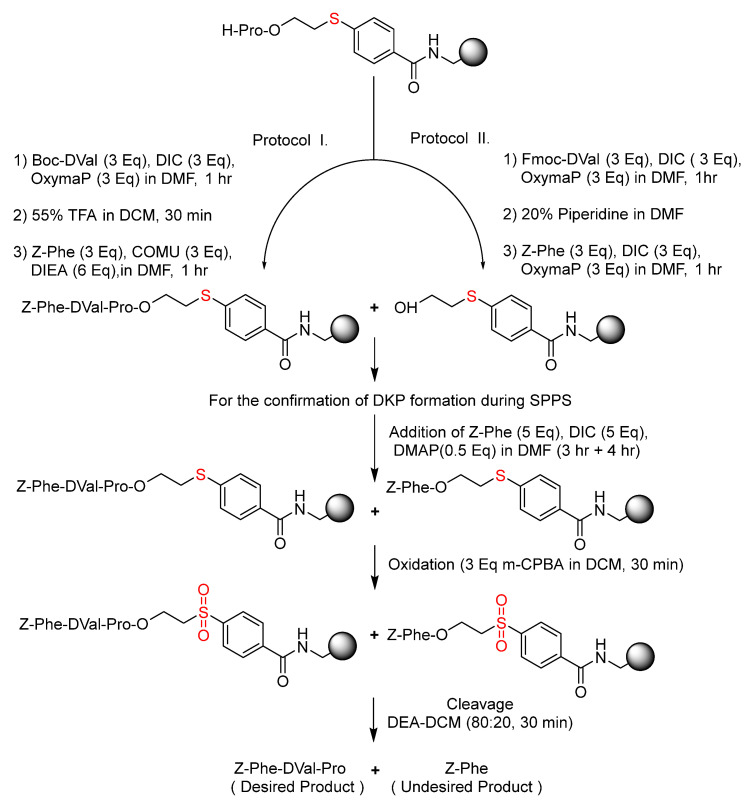
DKP formation experiments. (i) Reference of Z-L-Phe; (ii) products obtained using Boc and neutralization-coupling protocol (I); and (iii) product obtained using Fmoc-L-Phe, protocol (II). See Scheme S7 and Figures S34–S38.

As discussed earlier, in addition to showing stability in response to bases, ETB resin is also stable in response to treatments with an acid such as TFA and is therefore compatible with Boc chemistry. Although Fmoc chemistry generally offers more advantages than its Boc counterpart, there are cases where it is important to have the option of using Boc protection for the α-amino function. This is the case of diketopiperazine (DKP) formation, which is possibly the most severe side reaction in peptide chemistry, due to the stability of the six-membered ring of the cyclic dipeptide [31,32,33,34,35,36]. DKP formation takes place during the synthesis of *C*-terminal acid peptides and is more severe when the *C*-terminal amino acid is Pro and the subsequent amino acid has the D-configuration. Pro favors the cis-configuration of the peptide bond, and the six-member DKP ring containing one L and one D residue is more stable than when the two amino acids are of the same configuration [37]. Although this reaction can take place in an acid medium [38], it is much more severe in the presence of bases, such as the piperidine treatment to remove the Fmoc group of the second amino acid, and even during the neutralization step after removal of the Boc group of the same amino acid [39]. The minimization of this side reaction occurs when the second amino acid is incorporated with the α-amino protected with Boc, and after the removal of the Boc with TFA, the neutralization is avoided. Instead, an in situ neutralization-coupling strategy is performed. Thus, the third protected amino acid is activated and added to the trifluoroacetyl amino resin in the presence of a base such as *N*,*N*-Diisopropylamine (DIPEA) [40]. Once the salt has been neutralized, the acylation step with the third amino acid is used to compete favorably with the cyclization, resulting in the minimization of DKP formation [41]. Using the dipeptide D-Val-L-Pro as a model, we studied DKP formation using ETB resin in conjunction with Boc and Fmoc protection for D-Val. In the case of Boc, the third amino acid, Z-L-Phe, was introduced following an in situ neutralization-coupling protocol, while Z-Phe-OH was incorporated into the Fmoc-synthesis using a regular DIC and Oyxma Pure coupling protocol. If DKP forms, its release causes the formation of the initial hydroxy resin, which can be acylated in low extension with the Z-L-Phe (the esterification is more demanding than the amide formation and requires DMAP as a catalyst). To fully acylate the potential hydroxy groups formed, an extra acylation with Z-L-Phe, DIC, and DMAP is carried out in both resins. Oxidation of the resin followed by DEA treatment should render the tripeptide Z-L-Phe-D-Val-L-Pro in the absence of DKP formation or just Z-L-Phe in its presence. Figure 6 shows the scheme followed, and the HPLC profiles of both experiments. For the synthesis carried out with Fmoc-D-Val, only Z-L-Phe was obtained, thereby indicating 100% DKP formation. On the other hand, when Boc and in situ neutralization-coupling was performed, only 15% of Z-L-Phe formed. These results are consistent with others obtained using a similar in situ neutralization-coupling method with other protecting groups and/or resins [13,30,42]. 

It is important to highlight that DKP formation was not detected in the present work during the synthesis of peptides not containing *C*-terminal dipeptides prone to this side reaction (see below).

### 2.6. Synthesis of Unprotected Peptides Without TFA Cleavage 

The use of fluoride reagents such as TFA, trifluoromethanesulfonic acid (TFMSA), trifluoroethanol (TFE), or hexafluoroisopropanol (HFIP) for the cleavage of unprotected peptides (high content of TFA and TFMSA) or protected peptides (low content of TFA, TFE, and HFIP) is associated with two main problems in the context of sustainability and greenness. The first issue regards the waste generated by these compounds. For instance, TFA, which is widely used in SPPS, has a potentially hazardous nature. In this regard, the European Chemical Agency’s registration dossier revision indicated that TFA is resistant to biodegradation in water, showing no microbial breakdown under aerobic conditions [43]. TFA has been shown to display extreme persistence, exhibiting no degradation in year-long field studies or laboratory microcosms [44]. In forest experiments, TFA addition leads to significant drainage water export in upland forests and minimal flow in wetland forests, with varying retention rates in soil and vegetation [45]. On the other hand, the use of these reagents favors the presence of significant amounts, or even traces, of the fluorinated compounds in the final target. In many cases, the presence of such compounds is not acceptable in the cosmetic and food industry. 

The use of ETB resin can minimize the use of these fluoride reagents, and it can be used in earlier steps of the upstream process, which will result in the minimization of their presence in the final product. To demonstrate this concept, we prepared palmitoyl tripeptide and pentapeptide, used as anti-aging ingredients in cosmetics [46], and SAK, a peptide with biological activity of interest. 

In these cases, after elongation of the peptide, oxidation, and removal of the side-chain protecting groups with TFA, the peptide resin was gently washed with 5% DIPEA in DCM, before the final cleavage of the peptide with DEA. The washings with DIPEA should minimize the presence of trifluoroacetyl salts in the final product.

**Figure 7:**
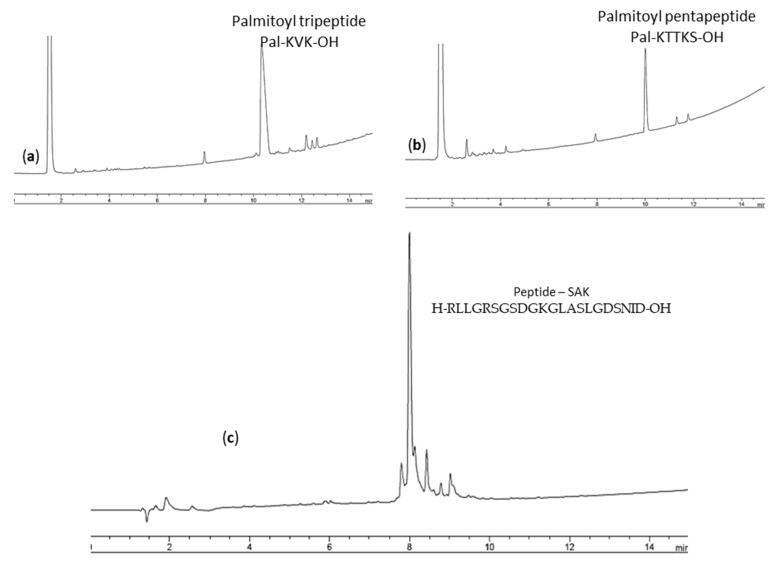
The HPLC profiles of palmitoyl tripeptide, pentapeptide-5, and SAK peptide synthesized using ETB resin through the following steps: (i) Fmoc/*t*Bu peptide elongation using DIC–Oxyma Pure (3 Eq.of each one, 1 h coupling); (ii) oxidation of the thio to the sulfone with *m*-CPBA (3 Eq.) in DCM for 10 min; (iii) side-chain deprotection with TFA/Triisopropylsilane (TIPS)/H_2_O (95/2.5/2,5, v/v/v) for 1 h followed by washing with DMF-DCM; (iv) neutralization with 5% DIPEA in DCM; and (v) cleavage with 80% DEA in DCM for 30 min. Gradient: 0/60 (v/v) of MeCN+0.1% TFA/H_2_O+0.1% TFA. Schemes S8 and S9 and Figures S38–S44.

The workup for SAK was similar to that used when the cleavage from the resin was carried out with TFA. After treatment with DEA, cold ether was added to the cleavage cocktail containing the resin and the precipitated peptide. The supernatant was removed by decantation after centrifugation, and cold ether was added again; the process was repeated two more times. Finally, SAK was extracted with H_2_O and lyophilized. As the two small palmitoyl peptides did not precipitate when cold ether was added, the supernatant after cleavage was removed by evaporation with a N_2_ stream, ether was added and removed with a N_2_ stream (these were repeated twice), and the peptide was extracted with 10% aqueous acetic acid and lyophilized. The small peaks around 6 min and between 12 and 13 min did not correspond to peptide material (Figure 7a,b).

SAK preparation was repeated using a CEM microwave-assisted automatic synthesizer following a regular program. The synthesis of the same peptide was also carried out with a CEM synthesizer using Wang resin. In both cases, the first three amino acids were incorporated manually to minimize the risk of DKP formation. In the case of Wang resin, the peptide was cleaved with TFA/TIPS/H_2_O (95/2.5/2,5, v/v/v) for 1 h. Both syntheses gave a similar purity profile. These observations indicate that the ETB resin is compatible with microwave synthesis (see Figures S41–S44).

## 3. Material and Methods

All amino acids were purchased from Iris Biotech-marktredwitz-Germanyand solvents from Honeywell-Seelze-Germany and were used without further purification, unless otherwise stated. The following Fmoc-AA-OH derivatives were used: Fmoc-Arg(Pbf)-OH, Fmoc-Lys(Boc)-OH, Fmoc-Asn(Trt)-OH, Fmoc-His(Trt)-OH, Fmoc-Trp(Boc)-OH, Fmoc-Asp(*t*Bu)-OH, Fmoc-Cys(Trt)-OH, Fmoc-Ser(*t*Bu)-OH, and Fmoc-Thr(*t*Bu)-OH. 

Nuclear magnetic resonance (NMR) spectra, proton (^1^H NMR) and carbon (^13^C NMR) were recorded on a Bruker AVANCE III 600 MHz spectrometer. Chemical shift values (*δ*) are expressed in parts per million (ppm). The multiplicities NMR description is: (s = singlet, d = doublet, dd = doublet of doublet, t = triplet, *J* = Coupling constant and ArH = aromatic proton) The deuterated dimethyl formamide (DMSO-d6) chemical shift for 1H NMR = 2.5 ppm and for 13C NMR = 39.52 ppm. Analytical HPLC was performed on an Agilent 1100 system using a Phenomenex C18 column (3 μm, 4.6 × 50 mm), and Chemstation (version B.04.03) software was used for data processing over a 5–95% gradient of MeCN (0.1% TFA)/H_2_O (0.1% TFA) over 15 min, flow rate: 1.0 mL/min, with detection at 220 nm. All mass spectrometry data were obtained from a Thermo Fisher Scientific UltiMate 3000 UHPLC-ISQ^TM^ EC single quadrupole mass spectrometer in positive-ion mode over a 5–95 % gradient of MeCN (0.1% HCOOH)/H_2_O (0.1% HCOOH) for 15 min unless otherwise specified. High-resolution mass spectrometry (HRMS) was performed using an Agilent MSD-TOF mass spectrometer in positive-ion mode.

### 3.1. Preparation of Methyl 4-Mercaptobenzoate

A mixture of 4 mercaptobenzoic acid (5.0 g), H_2_SO_4_ (0.063 mL) in MeOH (20 mL) was heated to 70 °C with stirring for 2 h, and the reaction mixture was monitored by TLC (SiO_2_) (MeOH/EtOAc, 5/95, v/v). The reaction mixture was cooled to 0°C in an ice bath and then neutralized with NaHCO_3_ to pH = 7. The solvent was concentrated to about 10 mL. Then, DCM (30 mL) and water (20 mL) were added. The aqueous phase was extracted with DCM (20 mL × 3). The combined organics were dried over MgSO_4_, filtered, and concentrated to render methyl 4-mercaptobenzoate (4.8 g) (96% yield).


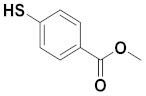
**HPLC** [5–95% of MeCN (0.1% TFA/H_2_O (0.1% TFA) over 15 min] t_R_ = 8.493 min; **^1^H NMR** (600 MHz, DMSO-*d_6_*): *δ* =7.81 (d, *J* = 7.9 Hz, 2H; ArH), 7.43 (d, *J* = 7.8 Hz, 2H; ArH), 3.83 (s, 3H, CH_3_), 3.34 (s, 1H, SH); **^13^C NMR** (150 MHz, DMSO-*d_6_*): 166.3, 140.8, 130.5, 130.1, 128.3, 126.7, 126.3, 52.4.

### 3.2. Preparation of Methyl 4-((2-Hydroxyethyl)thio)benzoate

A mixture of methyl 4-mercaptobenzoate (4.8 g), 2-bromoethanol (2.5 g), and Cs_2_CO_3_ (3.10 g) in *N*,*N*-dimethylformamide (DMF) (70 mL) was stirred at room temperature overnight. The mixture was filtered, and DCM (70 mL) was added. The solution was washed with water (50 mL × 5) and brine (50 mL × 2), dried over MgSO4, filtered, and concentrated. The crude product was purified by flash chromatography (silica gel; Petroleum ether/EtOAc = 20/1 to 2/1, v/v) to render methyl 4-((2-hydroxyethyl)thio)benzoate (4.4 g) as a white solid (91% yield).


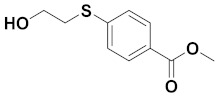
**HPLC** [5–95% of MeCN (0.1% TFA/H_2_O (0.1% TFA) over 15 min] t_R_ = 7.078 min; **^1^H NMR** (600 MHz, DMSO-*d_6_*): *δ* =7.85 (dd, *J* = 6.3 Hz, 2H; ArH), 7.42 (dd, *J* = 6.3 Hz, 2H, ArH), 5.04 (s, 1H, OH), 3.83 (s, 3H, CH_3_), 3.62 (t, *J* = 5.1 Hz, 2H), 3.14 (m, *J* = 6.4 Hz, 2H);^**13**^**C NMR** (150 MHz, DMSO-*d_6_*): 166.4, 144.6, 130.1, 126.5, 126.3, 60.1, 52.4, 34.2. **HRMS**: m/z: for C_10_H_12_O_3_S^+^: calcd: 213.0580 [M + H]^+^; found: 213.0577.

### 3.3. Preparation of 4-((2-Hydroxyethyl)thio)Benzoic Acid

Lithium hydroxide monohydrate (1.366 g) in water (70 mL) was added into a solution of methyl 4-((2-hydroxyethyl)thio)benzoate (4.4 g) in tetrahydrofuran (THF) (70 mL). The reaction mixture was stirred at room temperature overnight. The solvent was removed under reduced pressure, and the mixture was cooled in an ice bath and acidified to pH = 1–2 with conc. HCl. The aqueous phase was extracted with EtOAc (50 mL × 5). The combined organic phases were washed with brine (50 mL × 2), dried over MgSO_4_, filtered, and concentrated to render 4-((2-hydroxyethyl)thio)benzoic acid (4.0 g) as a white solid (90% yield).


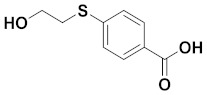
**HPLC** [5–95% of MeCN (0.1% TFA/H_2_O (0.1% TFA) over 15 min] t_R_ = 5.448 min; **^1^H NMR** (600 MHz, DMSO-*d_6_*): *δ* = 12.8 (s, 1H, COOH), 7.84 (dd, *J* = 8.3 Hz, 2H; ArH), 7.39 (dd, *J* = 8.4 Hz, 2H, ArH), 5.03 (s, 1H, OH), 3.61 (dd, *J* = 7.86 Hz, 2H, CH_2_), 3.13 (dd, *J* = 7.9 Hz, 2H, CH_2_);^**13**^**C NMR** (150 MHz, DMSO-*d_6_*): 167.5, 143.9, 130.2, 127.6, 126.5, 60.1, 34.2. **HRMS**: m/z: for C_9_H_9_O_3_S^−^: calcd: 197.0278 [M − H]^−^; found: 197.0282. 

### 3.4. General Peptide Synthesis Protocol

Washings and Fmoc-removal steps were carried out with 1 mL of solvent/0.1 g of resin (10 vol), while coupling and TFA treatment were carried out with 0.5 mL of solution/0.1 g of resin (5 vol).

### 3.5. ETB Linker Attachment to Aminomethyl Polystyrene Resin 

Aminomethyl polystyrene resin (0.1 mmol) was swollen in 5% DIPEA-DCM for 5 min, and then the solvent was drained and washed with DCM (× 3) and DMF (× 3). ETB linker (2 Eq.) and HOBt (2 Eq.) were dissolved in DMF, and the mixture was added to the resin, followed by DIC (2 Eq.). The mixture was put on a shaker and agitated for 1 h. The mixture was drained, and the resin beads were washed with DMF (× 3) and DCM (× 3) to render ETB resin.

### 3.6. Coupling First Amino Acid to ETB Resin

ETB resin (0.1 mmol) was swollen in DCM for 5 min, then the solvent was drained and washed with DCM (× 3) and DMF (× 3). Fmoc-AA-OH (5 Eq.) and DMAP (0.5 Eq.) were dissolved in DMF; the mixture was added to the resin, and DIC was added (5 Eq.). The mixture was put on a shaker and agitated for 2 h. The mixture was drained, and the resin beads were washed with DMF (× 5) and DCM (× 3). The remaining hydroxy groups of ETB resin were capped by the addition of Ac_2_O (5 Eq.) and DIPEA (10 Eq.) in DCM, and the resin was agitated for 1 h. The mixture was drained, and the resin beads were washed with DCM (× 3) and DMF (× 3). 

### 3.7. Fmoc Removal 

The Fmoc group was removed with a 20% solution of piperidine in DMF (2 × 5 min). The mixture was drained, and the resin beads were washed with DMF (× 5).

### 3.8. Elongation of Peptide

Fmoc-AA-OH (3 Eq.) and Oxyma Pure (3 Eq.) were dissolved in DMF, and DIC (3 Eq.) was added. After a pre-activation period of 3 min, the mixture was added to the resin, put on a shaker, and agitated for 1 h (unless otherwise stated). The mixture was drained, and the resin beads were washed with DMF (× 5). 

### 3.9. Oxidation of ETB Resin (Sulfide to Sulfone)

The oxidation step was always carried out with the protected peptide anchored to the resin, that is, before removing the side-chain protecting groups.

The fully protected peptide resin was treated with *m*-CPBA (3 Eq.) in DCM for 10 min at RT, and the resin was drained and washed with DCM (× 3) and DMF (× 3)

### 3.10. Removal of the Side-Chain Protecting Groups

The removal of the side-chain protecting groups, if needed (unprotected peptides), was performed after the oxidation step. The protected peptide resin was treated with TFA/TIPS/H_2_O (95/2.5/2,5, v/v/v) for 1 h at RT; the resin was drained, washed with DCM (× 5), neutralized with 5% DIPEA in DCM for 5 (× 3), washed with DCM (× 5), and drained.

### 3.11. Cleavage of Unprotected Peptide from ETB Resin

The peptide resin was washed with DCM (3 ×) and treated with 80% DEA in DCM for 30 min. Then, cold diethyl ether (10 vol) was added to the cleavage mixture with the consequent precipitation of the unprotected peptide. The mixture was centrifuged, and the solution was decanted. The operation was repeated one more time, and then the mixture of the precipitated peptide and the resin was extracted with 10% aqueous AcOH, and the solution was filtered off and lyophilized. 

### 3.12. Cleavage of Protected Peptide from ETB Resin

The protected peptide resin was treated similarly to the unprotected peptide resin, but after the cleavage with neat DEA for 30 min, the solution was filtered off and evaporated to dryness. The oil was taken in diethyl ether and evaporated to dryness. The operation was repeated several times until a solid was obtained. 

## 4. Conclusions

Here, we developed a safety-catch strategy for peptide synthesis. This involved the use of a phenylthioethanol moiety (ETB linker) that forms a bond with the carboxylic group of the first protected amino acid, which is stable in response to secondary amines, such as piperidine, and TFA. After oxidation of the thioether moiety to the corresponding sulfone, the same bond is still stable in response to TFA, but upon treatment with secondary amines, it undergoes a β-elimination reaction, rendering the free acid component. 

The two reactions, namely oxidation of the sulfide to sulfone and cleavage of the peptide from the ETB resin, were studied in detail using a multi-detachable linker. In this regard, 3 Eq. of *m*-CPBA in DCM for 30 min proved optimal. These conditions are compatible with peptides containing His and Trp. Once the sulfone has been obtained, the peptide resin can be treated with > 60% DEA, 10% piperidine, or 10% 4-methylpiperidine using DCM as a solvent to release the peptide almost quantitatively and render the protected peptides. Neat DEA, which is inexpensive and not restricted like piperidine, has the advantage of eliminating the need for any solvent. For the preparation of unprotected peptides, the side-chain protecting groups can be removed with TFA in the presence of scavengers (in the case of a simple protecting group such as *t*Bu, HCl in dioxane can also be used). Washings with 5% DIPEA solutions should minimize the TFA salt content in the final product. After this, cleavage with DEA will render unprotected peptides. As initial examples of this new strategy, the ETB linker was prepared and applied to the SPPS in manual and microwave-assisted automatic synthesizers. The ETB resin allowed a combination of Fmoc and Boc chemistry, which can be very useful for the minimization of side reactions such as DKP formation. 

We anticipate that the ETB linker will be useful for optimizing the synthesis of *C*-terminal acid peptides, particularly in relation to the use of polyfluoroalkyl substances (PFASs). For the synthesis of unprotected peptides, the removal of the side-chain protecting groups when the peptide is still anchored to the resin can be achieved by continuous flow, limiting the contact of carbocations with sensitive amino acids and therefore minimizing the formation of side products. 

Our laboratory is currently working on the substitution of hazardous solvents such as DMF and DCM with greener ones such as *N*-butylpyrrolidone (NBP) and γ-valerolactone (GVL). The use of non-PFASs for the removal of the side-chain protecting groups of the trifunctional amino acids is being carried out in our laboratory.

## Figures and Tables

**Table 1:** HPLC analysis conducted for the cleavage study to determine the percentage of peptide recovery. Gradient: 5/95 (v/v) of MeCN+0.1% TFA/H_2_O+0.1% TFA.

Basic Cleavage Conditions rt, 20 min	Yield % *
100% DEA,	95.1
80% DEA—DCM	93.5
50% DEA—DCM	91.6

* Yields were calculated using an external standard. See Figures S16 and S17 for LC-MS.

**Table 2:** Cleavage study using various basic conditions.

Basic Cleavage Conditions, rt, 30 min	Percentage of Cleaved Protected Peptide (7) (Ac-Y(*t*Bu)GGFL-OH) * (%)	Percentage of Uncleaved Peptide (Ac-YGGFL-ETB-Linker(O=S=O)-GFL-NH_2_ (%)
**100% DEA**	100	0
**80% DEA-**DCM	>99	<1%
**50% DEA-**DCM	98	2
**20% DEA-**DCM	63	37
**5% DEA-**DCM	15	85
**10% Piperidine-**DCM	96	4
**10% 4-methylpiperidine–DMF**	93	7
**60% morpholine–DMF**	47	53

* Indirectly calculated by the ratio of the adduct of the base with vinylsufonyl moiety [(**8**) in the case of DEA].

## Data Availability

Data are contained within the article.

## Supplementary Materials

The following supporting information can be downloaded at: https://www.mdpi.com/article/10.3390/ijms26052210/s1, Materials and methods. General protocol and characterization of linkers and peptides.
