# Supplementary material for: Base-Labile Safety-Catch Linker: Synthesis and Applications in Solid-Phase Peptide Synthesis
Source: Int J Mol Sci. 2025 Feb 28;26(5):2210. doi: 10.3390/ijms26052210 (PMC11899736; doi:10.3390/ijms26052210)
Supplement: Supplementary file 1 [file ijms-26-02210-s001.zip › ijms-3482974-supplementary.pdf]

Supporting Information

Base labile safety catch linker. Synthesis and Applications in Solid Phase Peptides Synthesis

Sikabwe Noki,[1,2] Hossain Saneii,[3] Beatriz G. de la Torre,[2*] Fernando Albericio[1,4*]

[1] Peptide Science Laboratory, School of Chemistry and Physics, University of KwaZulu-Natal, Westville, Durban 4000, South Africa.

[2] School of Laboratory Medicine and Medical Sciences, College of Health Sciences, University of KwaZulu-Natal, Durban 4041, South Africa.

[3] AAPPTec, 6309 Shepherdsville Road, Louisville, Kentucky 40228, USA.

[4] Department of Organic Chemistry, University of Barcelona, 08028 Barcelona, Spain.

*Email: albericio@ukzn.ac.za ; garciadelatorreb@ukzn.ac.za

HPLC NMR Spectra Chromatograms and HRMS Spectra

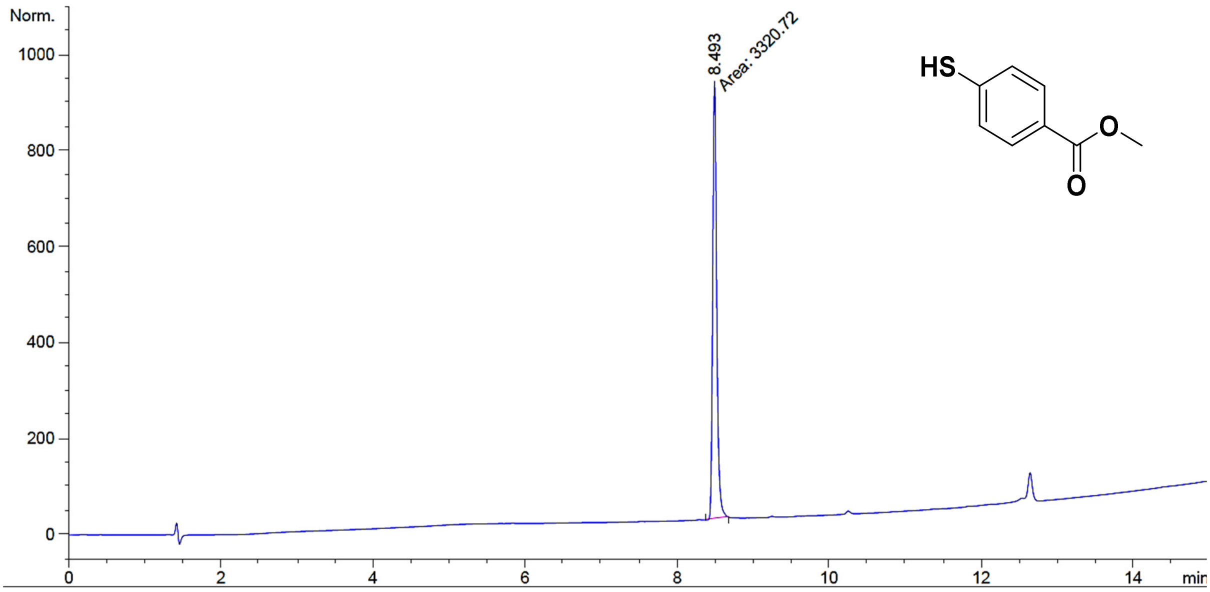

Figure S1. HPLC for **methyl 4-mercaptobenzoate**

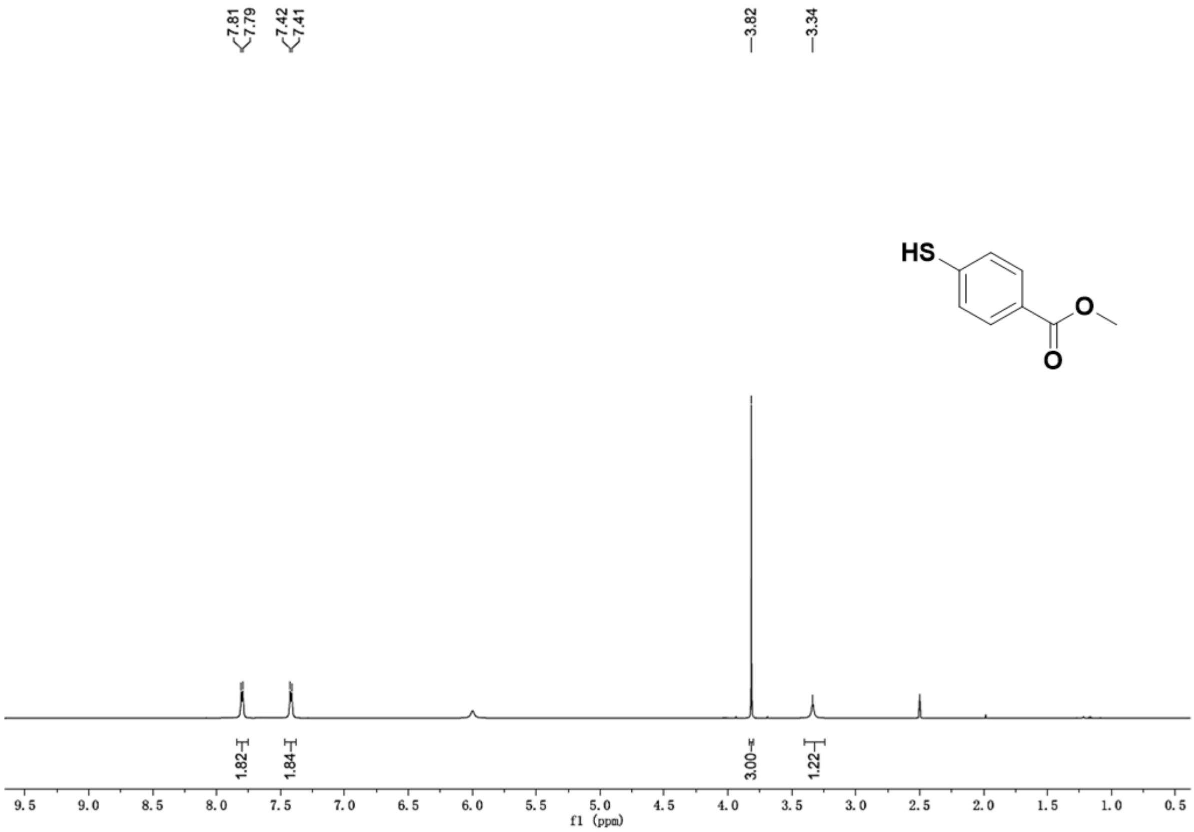

Figure S2. ^{1}H NMR, DMSO, 600 MHz for methyl 4-mercaptobenzoate

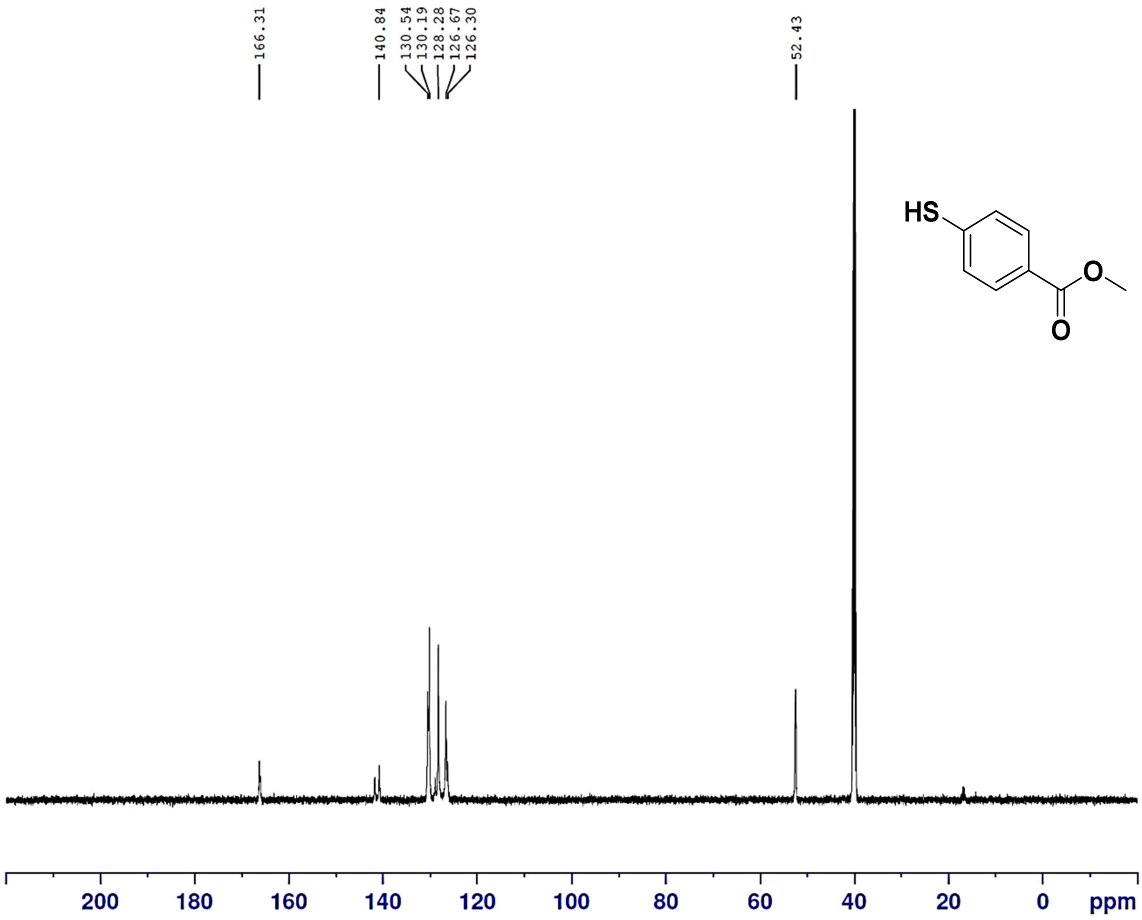

Figure S3. ^{13}C NMR, DMSO, 600 MHz for methyl 4-mercaptobenzoate

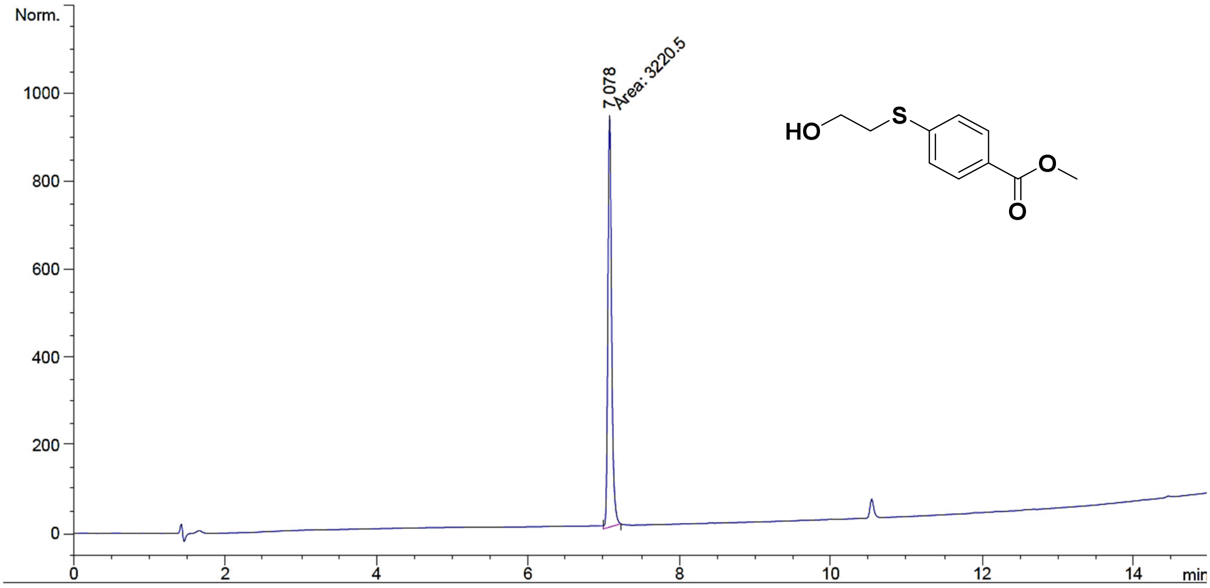

Figure S4. HPLC for methyl 4-((2-hydroxyethyl) thio) benzoate

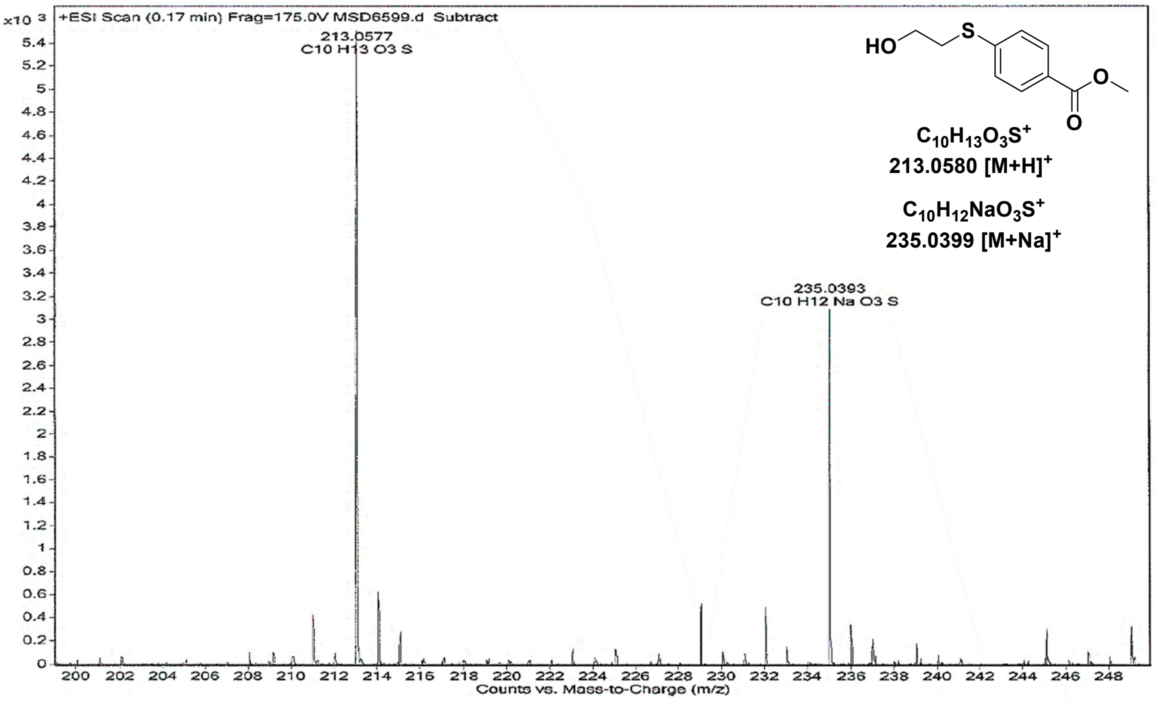

Figure S5. HRMS for methyl 4-((2-hydroxyethyl) thio) benzoate

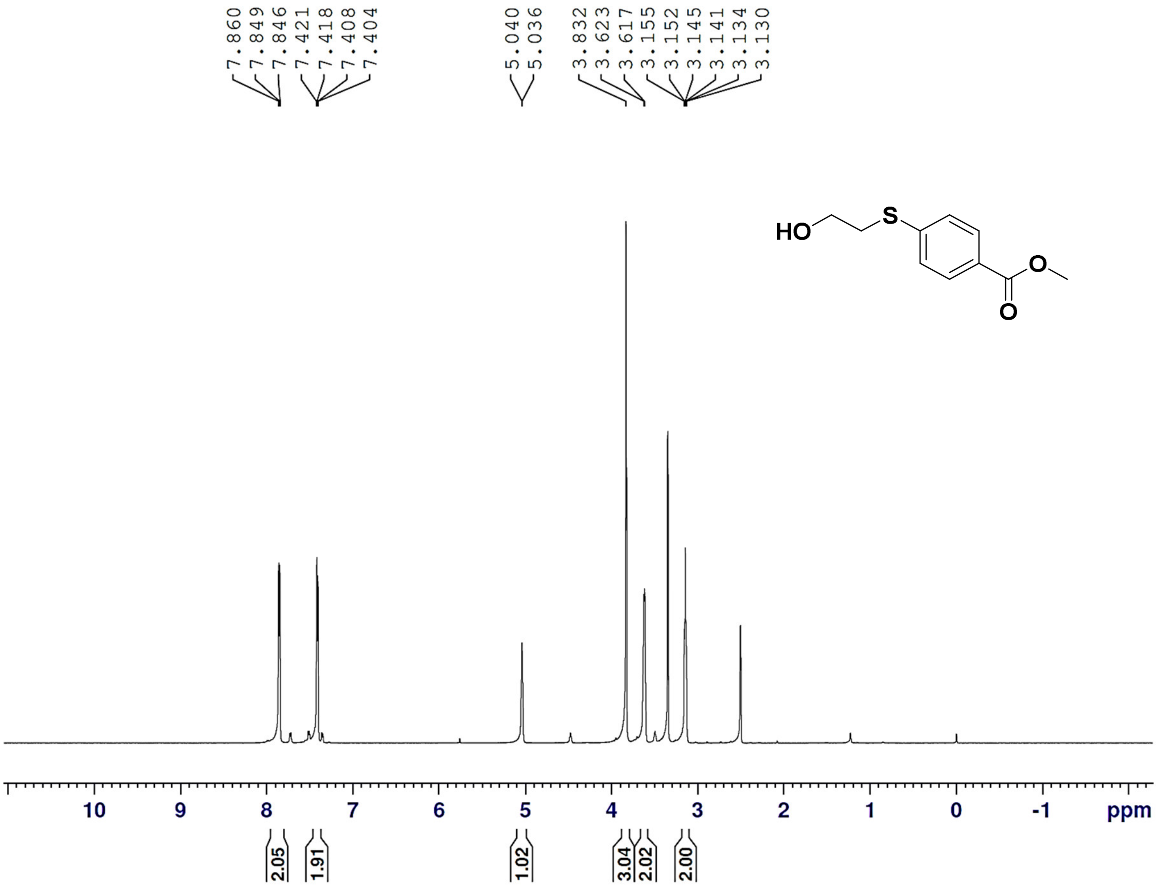

Figure S6. ^{1}H NMR, DMSO, 600 MHz for methyl 4-((2-hydroxyethyl) thio) benzoate

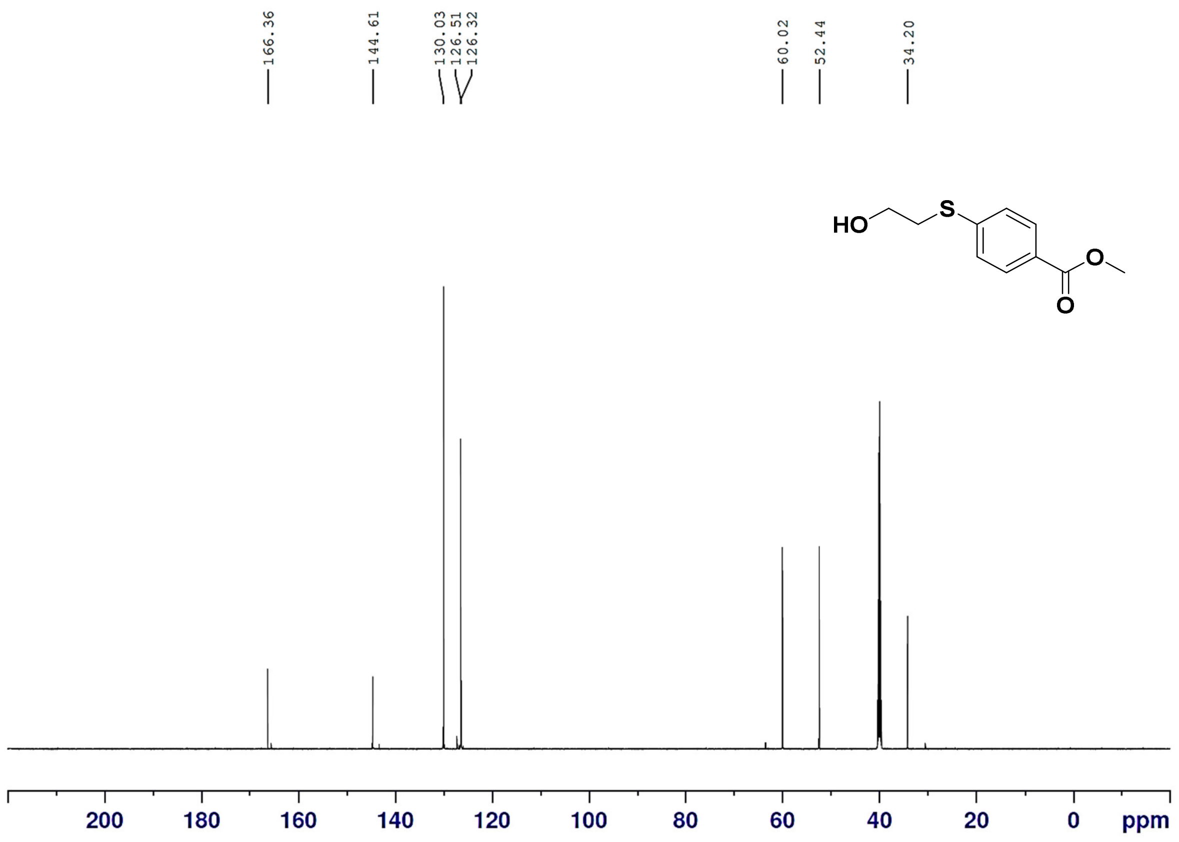

Figure S7. ^{13}C NMR, DMSO, 600 MHz for methyl 4-((2-hydroxyethyl) thio) benzoate

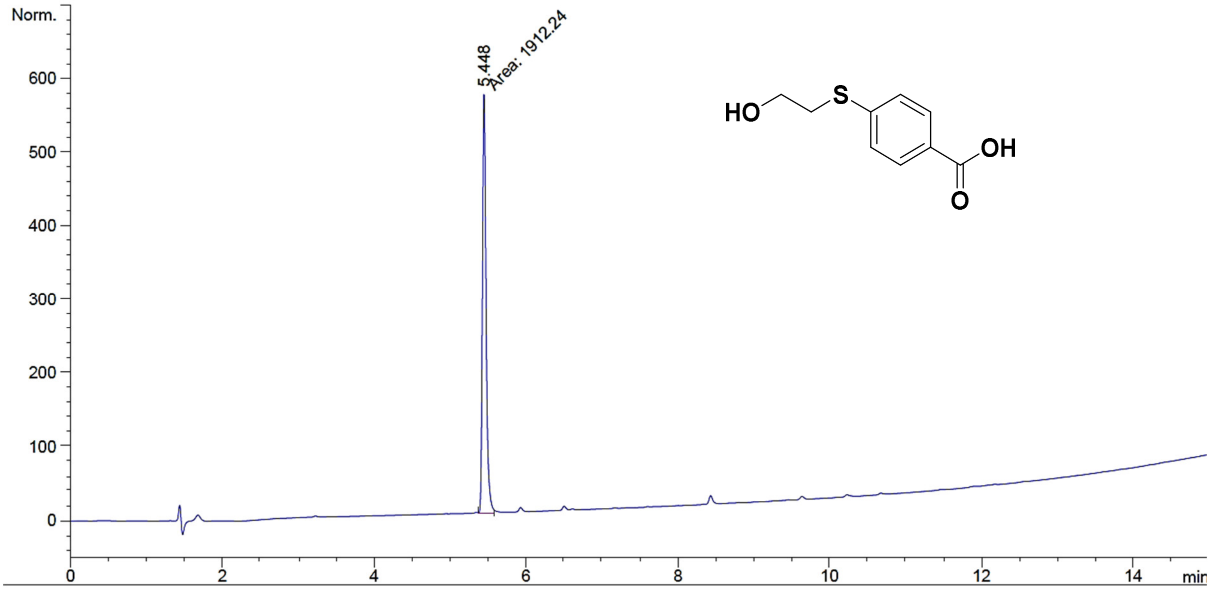

Figure S8. HPLC for 4-((2-hydroxyethyl) thio) benzoic acid

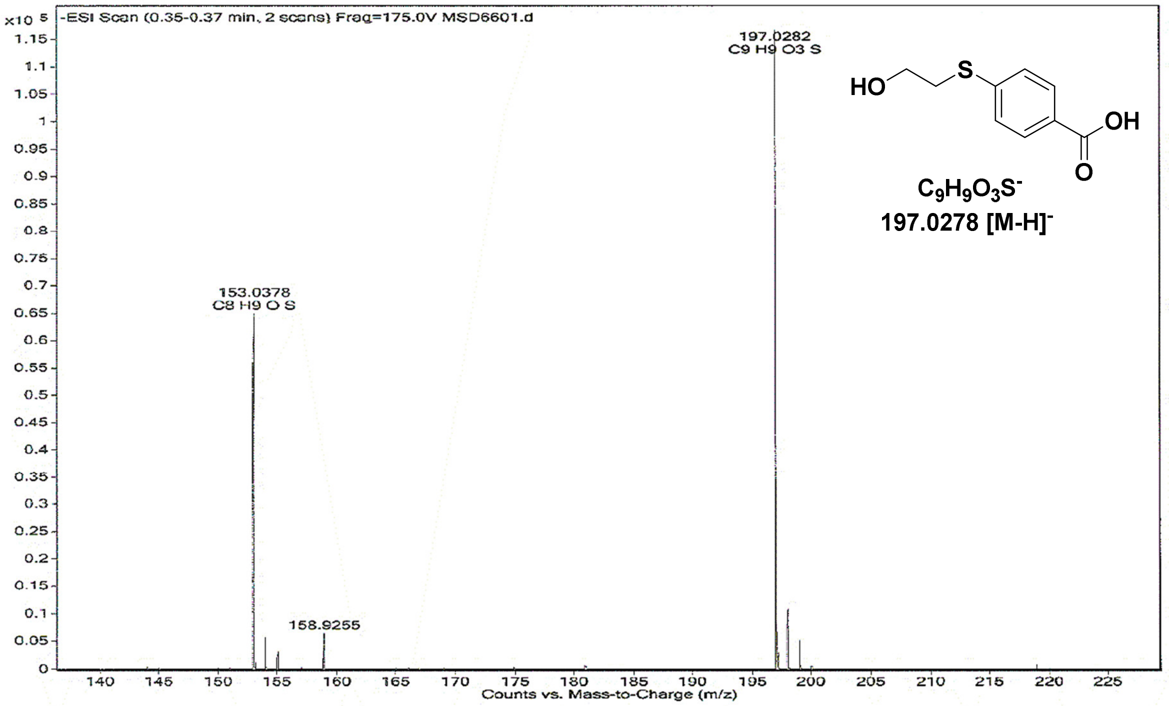

Figure S9. HRMS for 4-((2-hydroxyethyl) thio) benzoic acid

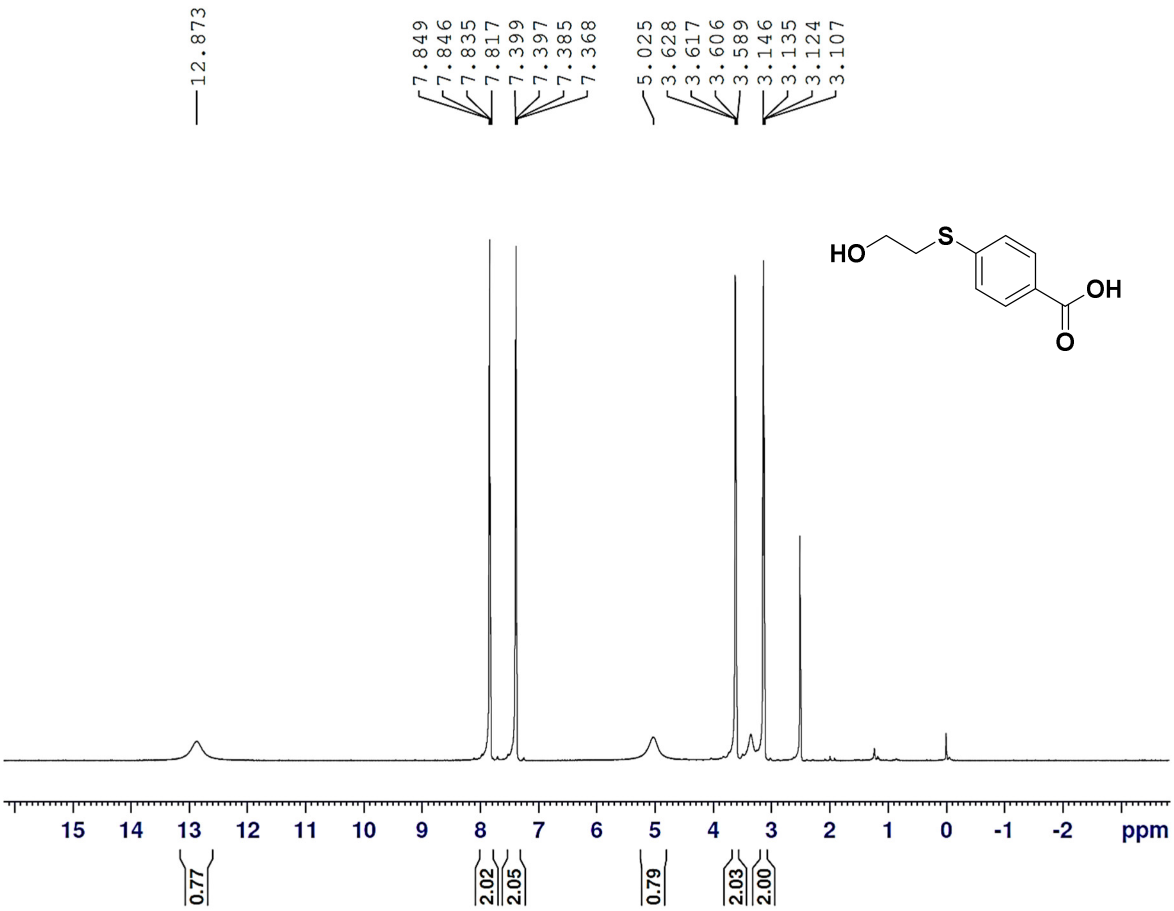

Figure S10. ^{1}H NMR, DMSO, 600 MHz for 4-((2-hydroxyethyl) thio) benzoic acid

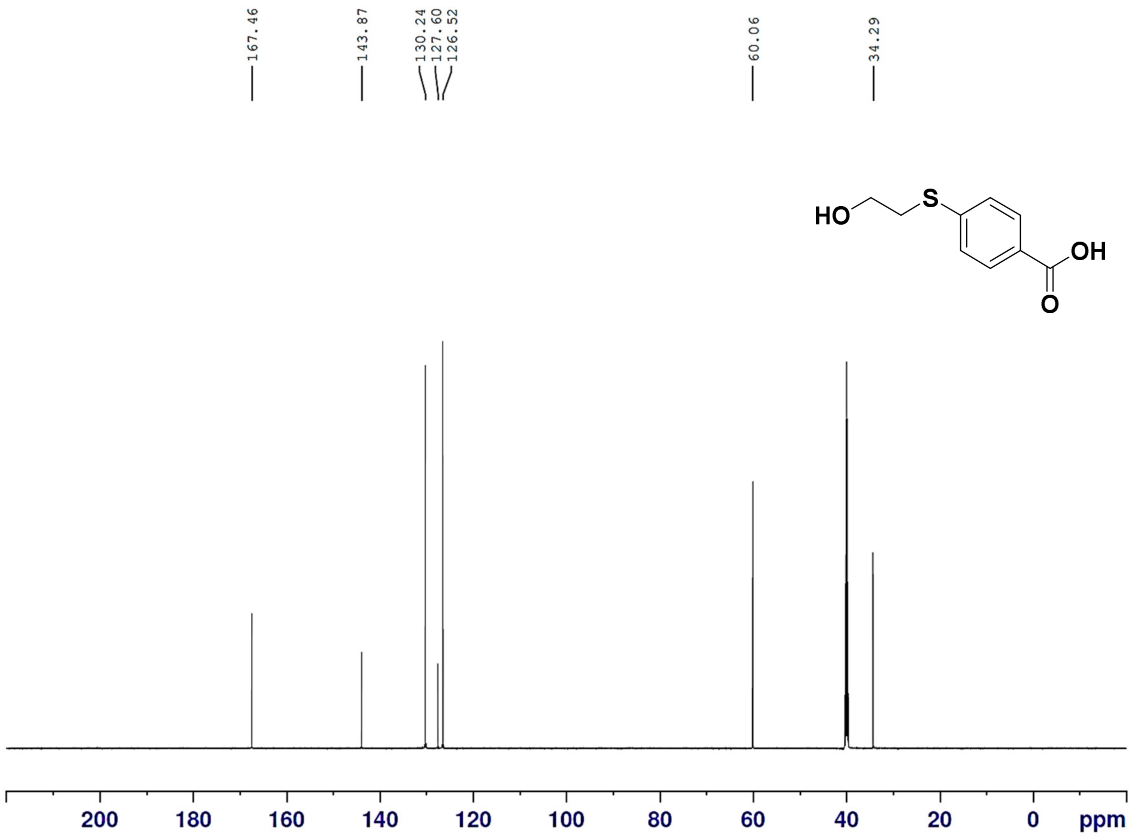

Figure S11. ^{13}C NMR, DMSO, 600 MHz for 4-((2-hydroxyethyl) thio) benzoic acid

General peptide synthesis protocol

Washings and Fmoc-removal steps were carried out with 1 mL of solvent/0.1 g of resin (10 vol), while coupling and TFA treatment with 0.5 mL of solution/0.1 g of resin (5 vol).

ETB linker attachment to aminomethyl polystyrene resin

Aminomethyl polystyrene resin (0.1 mmol) was swollen in 5% DIEA- CH_2Cl_2 for 5 min, then the solvent was drained washed with CH_2Cl_2 (x3) and DMF (x3). ETB linker (2 eq.), HOBt (2 eq.) was dissolved in DMF, the mixture was added to the resin, followed by DIC (2 eq.). The mixture was put on shaker and agitated for 1 hr. The mixture was drained, and the resin beads washed with DMF (x3) and CH_2Cl_2 (x3). render ETB-resin.

Coupling first amino acid to ETB-resin

ETB-resin (0.1 mmol) was swollen in CH_2Cl_2 for 5 min, then the solvent was drained washed with CH_2Cl_2 (x3) and DMF (x3). Fmoc-AA-OH (5 eq.), DMAP (0.5 eq.) was dissolved in DMF, the mixture was added to the resin and DIC was added (5 eq.). The mixture was put on shaker and agitated for 2 hr. The mixture was drained, and the resin beads washed with DMF (x5) and CH_2Cl_2 (x 3). The remaining hydroxy groups of ETB-resin were capped adding Ac_2O (5 eq.), and DIEA (10 eq.) in CH_2Cl_2 the resin was agitated for 1hr. The mixture was drained, and the resin beads washed with CH_2Cl_2 (x3), DMF(x3).

Fmoc removal

The Fmoc group was removed with a 20% solution of piperidine in DMF (2 x 5 min). The mixture was drained, and the resin beads washed with DMF (x5).

Elongation of peptide:

Fmoc-AA-OH (3 eq.) and Oxyma Pure (3 eq.) were dissolved in DMF, DIC (3 eq.) was added. After a pre-activation period of 3 min, the mixture was added to the resin, put on shaker, and agitated for 1 hr (unless otherwise stated). The mixture was drained, and the resin beads were washed with DMF (x5).

Oxidation of ETB-resin (sulfide to sulfone)

The oxidation step is always carried out with the protected peptide anchored to the resin, that is, before removing the side-chain protecting groups.

The fully protected peptide resin was treated with m-CPBA (3 eq,) in CH_2Cl_2 for 10 min at RT, the resin was drained and washed with CH_2Cl_2 (x3) and DMF (x3)

Removal of the side-chain protecting groups

The removal of the side-chain protecting groups, if needed (unprotected peptides), is done after the oxidation step. The protected peptide resin was treated with TFA-TIS-H_2O (95:2.5:2.5) for 1 h at RT, the resin was drained, washed with CH_2Cl_2 (x5), neutralized with 5% DIEA in CH_2Cl_2 for 5 (x3), washed with CH_2Cl_2 (x5) and drained.

Cleavage of unprotected peptide from ETB-resin

The peptide resin was washed with CH_2Cl_2 (3 x), and treated with 80% DEA in CH_2Cl_2 for 30 min. Then, cold diethyl ether (10 vol) was added to the cleavage mixture with the consequent precipitation of the unprotected peptide. The mixture was centrifuged, and the solution was decanted. The operation

was repeated one more time, then the mixture of the precipitated peptide and the resin was extracted with 10% aqueous AcOH, and the solution was filtered off and lyophilized.

Cleavage of protected peptide from ETB-resin

The protected peptide resin was treated similarly to the unprotected peptide resin, but after the cleavage with neat DEA for 30 min the solution was filtered off and evaporated to dryness. The oil was taken in diethyl ether and evaporated to dryness. The operation will be repeated several times until a solid is obtained.

Investigation of Linker activation (oxidation of sulfide to sulfone)

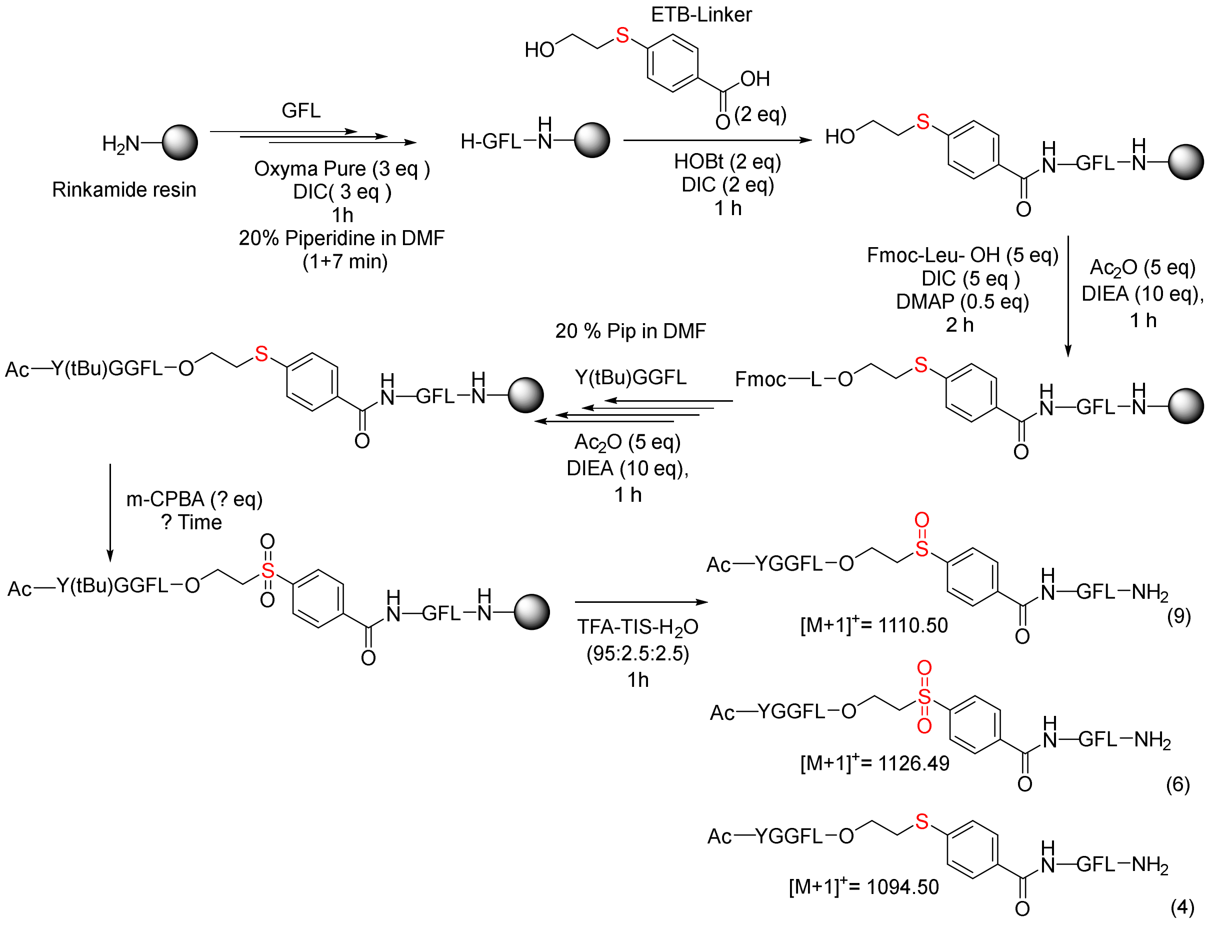

Scheme S1: Oxidation investigation of sulfide to sulfone using m-CPBA in DCM

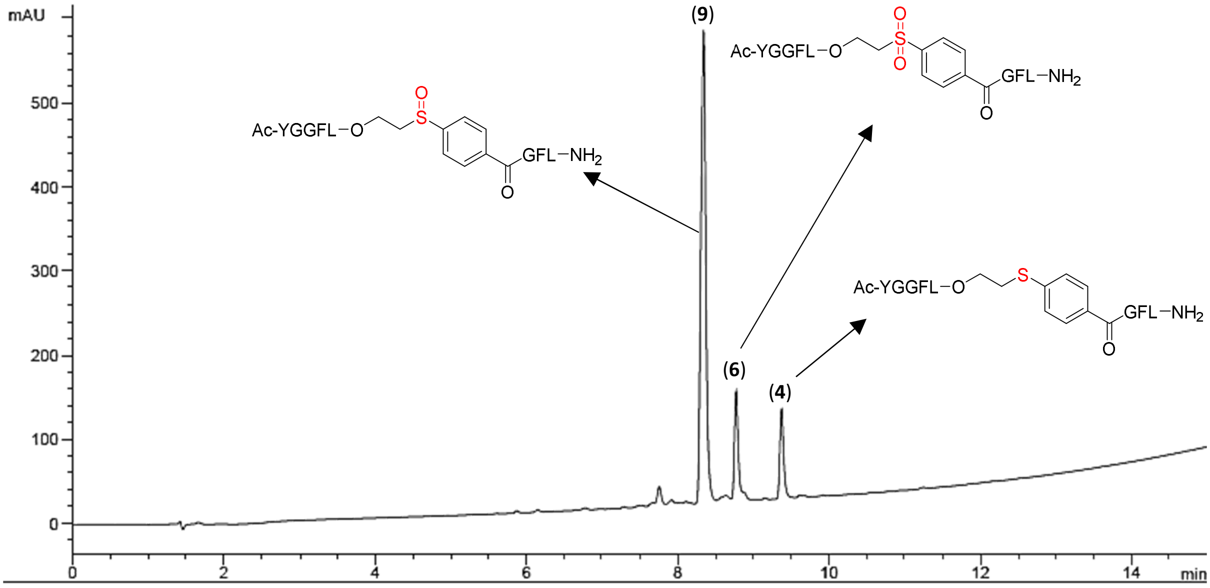

Figure S12. HPLC for oxidation study linker from sulfide to sulfone using peptide (Ac-YGGFL-NH_2), condition: - m-CPBA (1 eq) at 10 min; cleavage: TFA-TIS-H_2O (95:2.5:2.5), 1h.

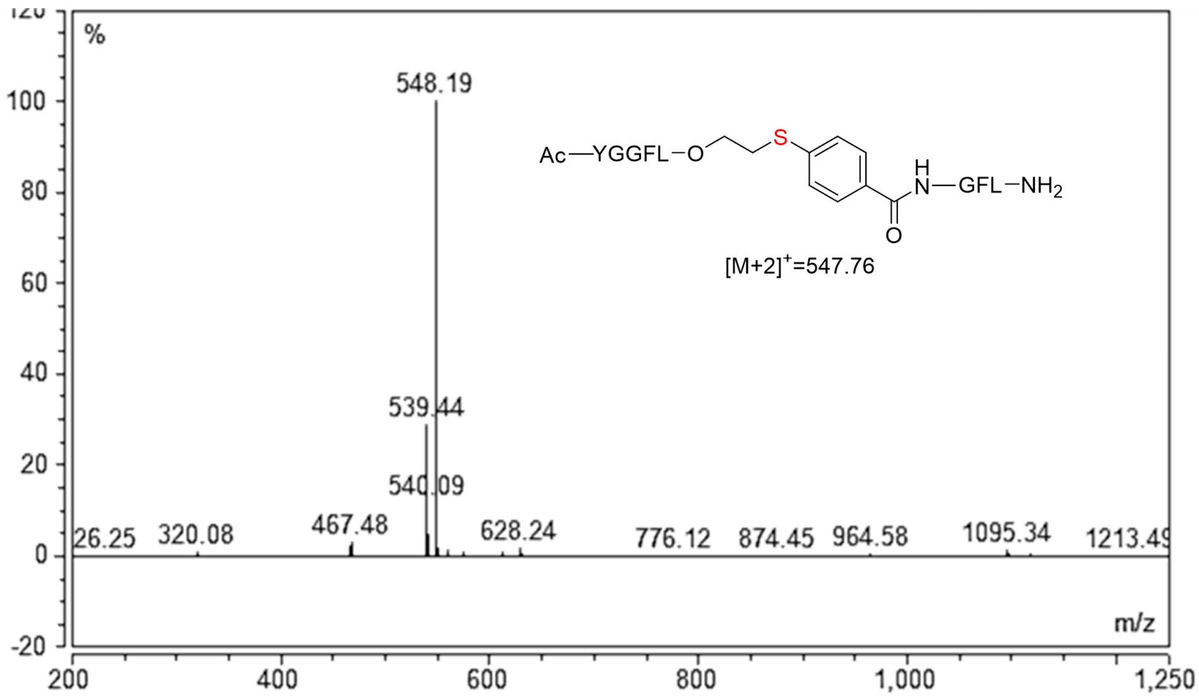

Figure S13. LCMS for peptide **(4)**

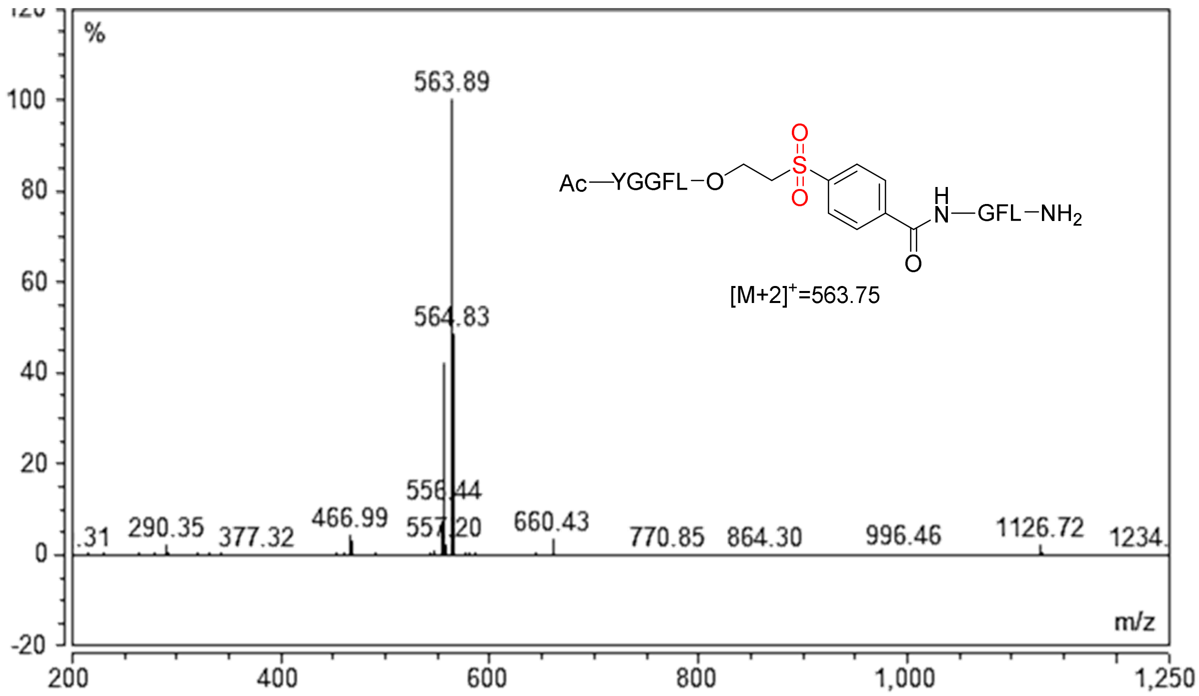

Figure S14. LCMS for peptide **(6)**

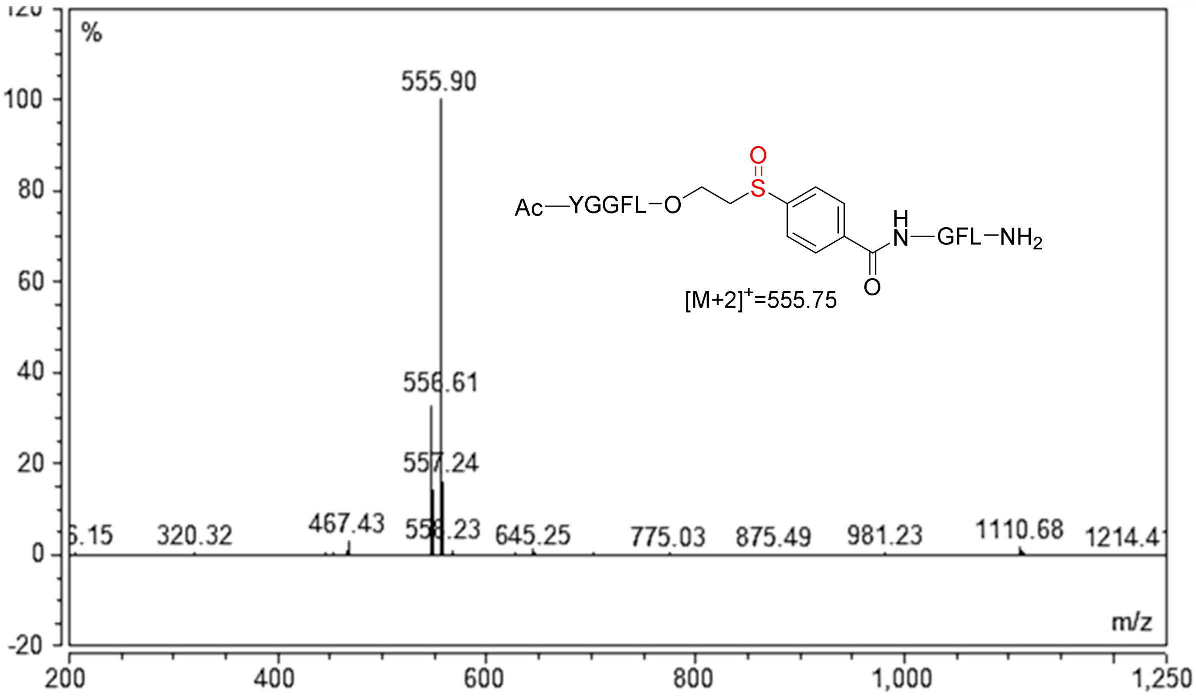

Figure S15. LCMS for peptide **(9)**

Peptide cleavage from activated ETB-Linker

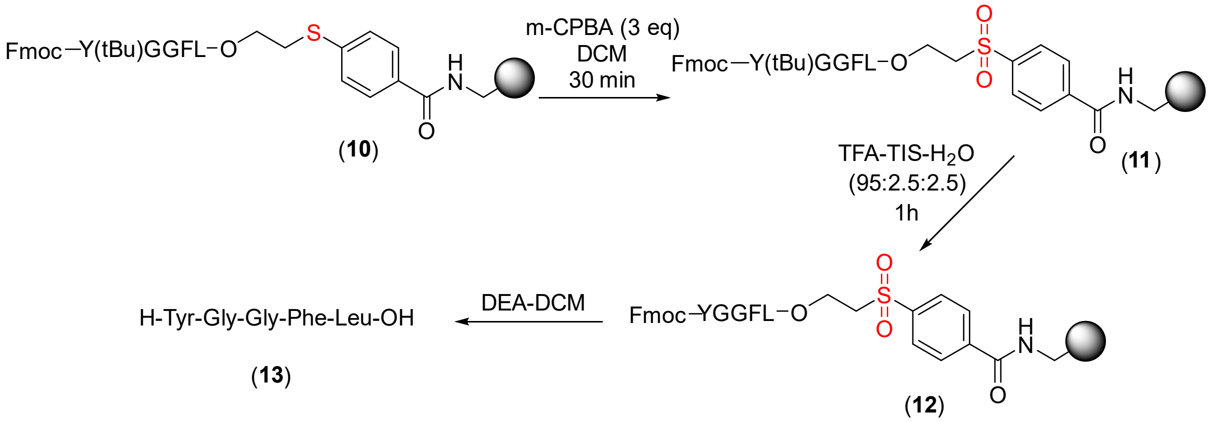

Scheme S2: Cleavage investigation using various basic conditions.

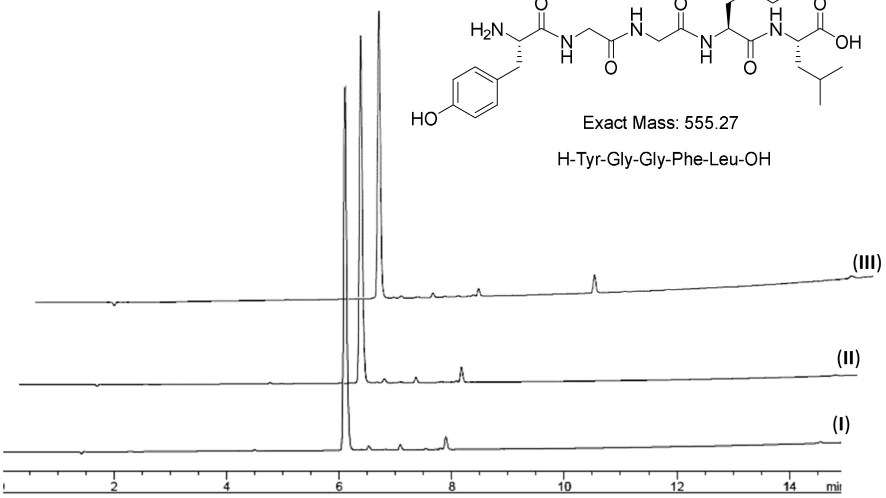

Figure S16: HPLC of cleavage study using various basic conditions: – all Cleavage time was 30 min; **(I)** 100 % DEA, **(II)** 80 % DEA-DCM, **(III)** 50 % DEA-DCM.

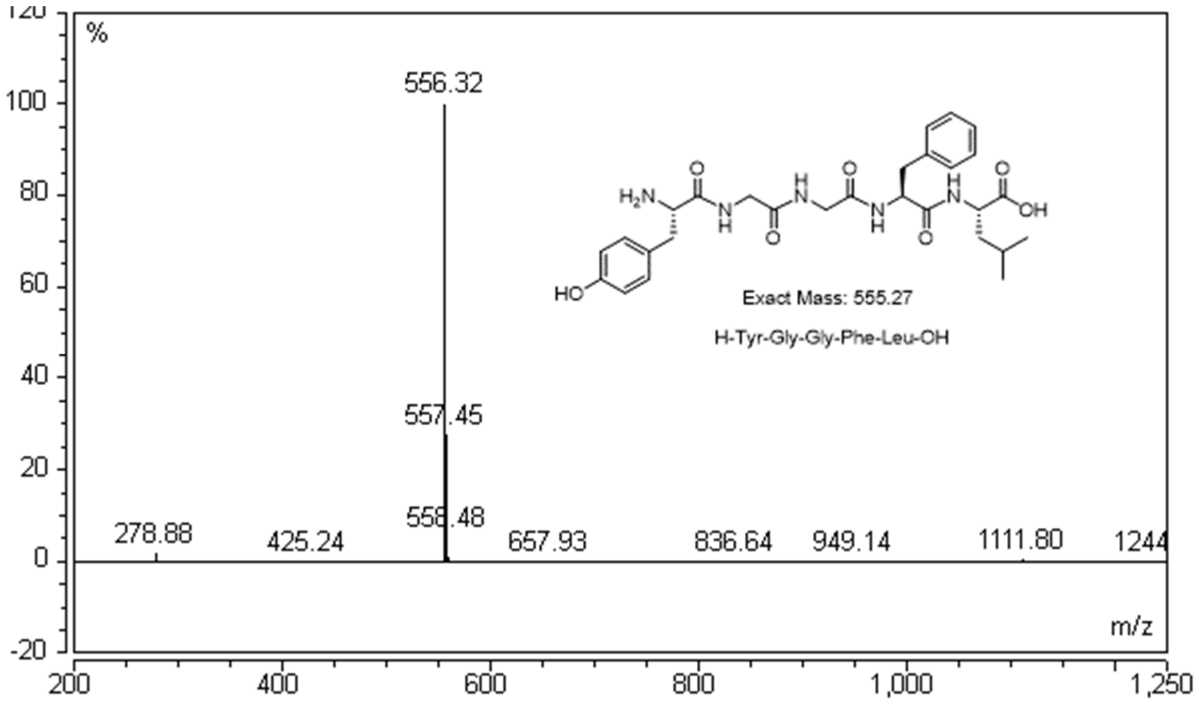

Figure S17. LCMS for H-Tyr-Gly-Gly-Phe-Leu-OH

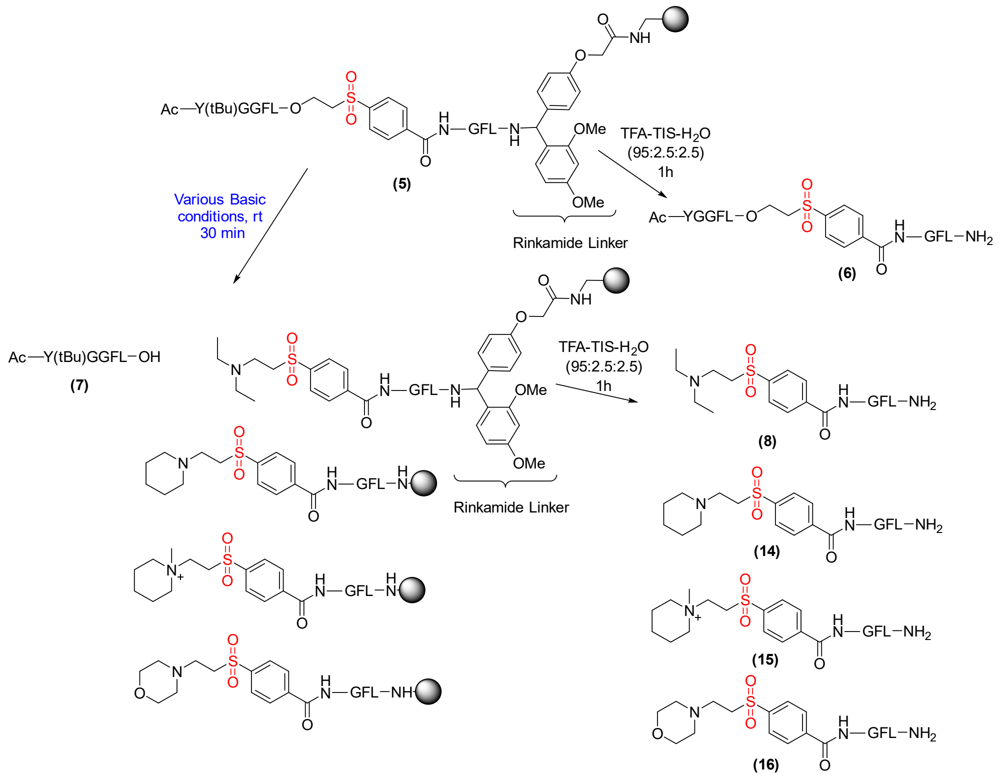

Scheme S3: Cleavage investigation using various basic conditions on multi detachable linkers peptide

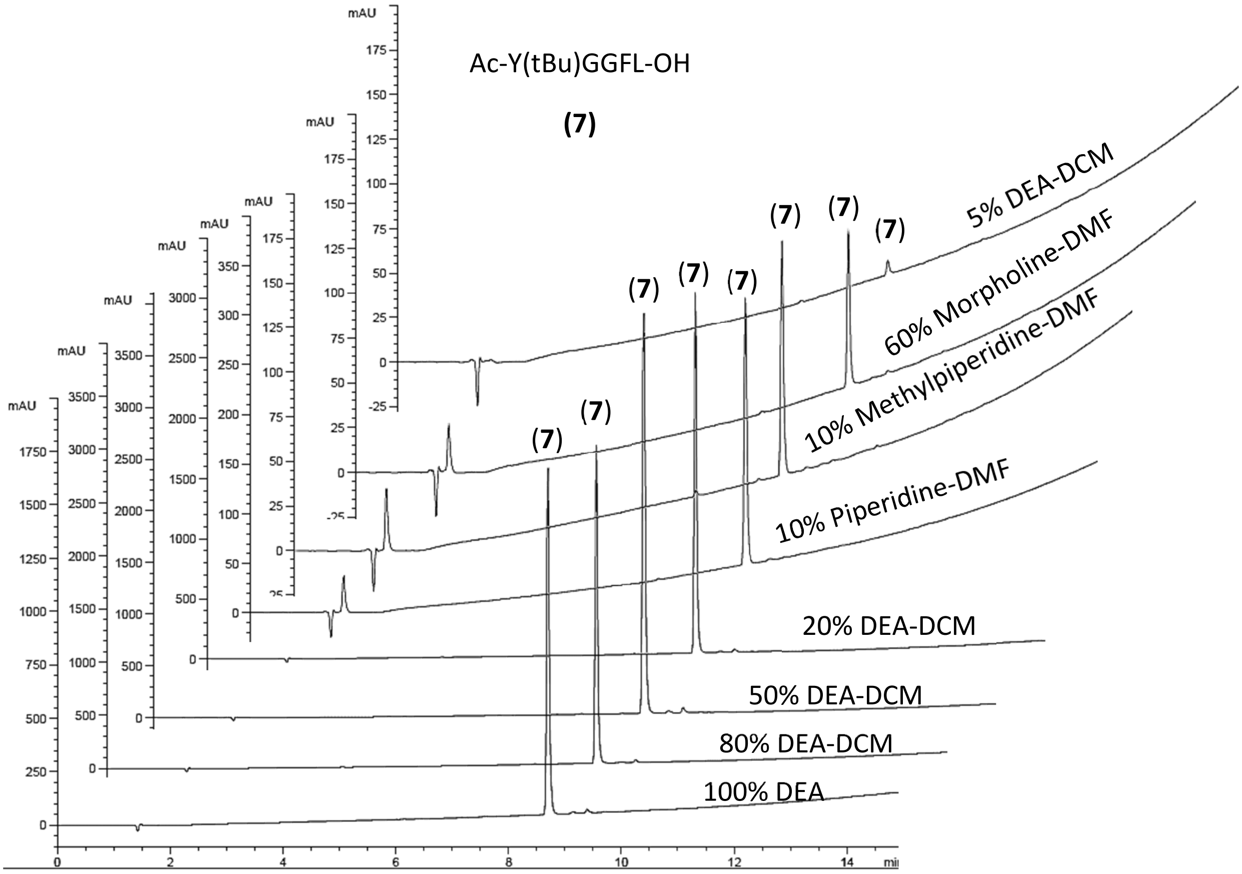

Figure S18. HPLC of Ac-Y(tBu)GGFL-OH, cleavage study using various basic conditions from multi detachable linkers peptide

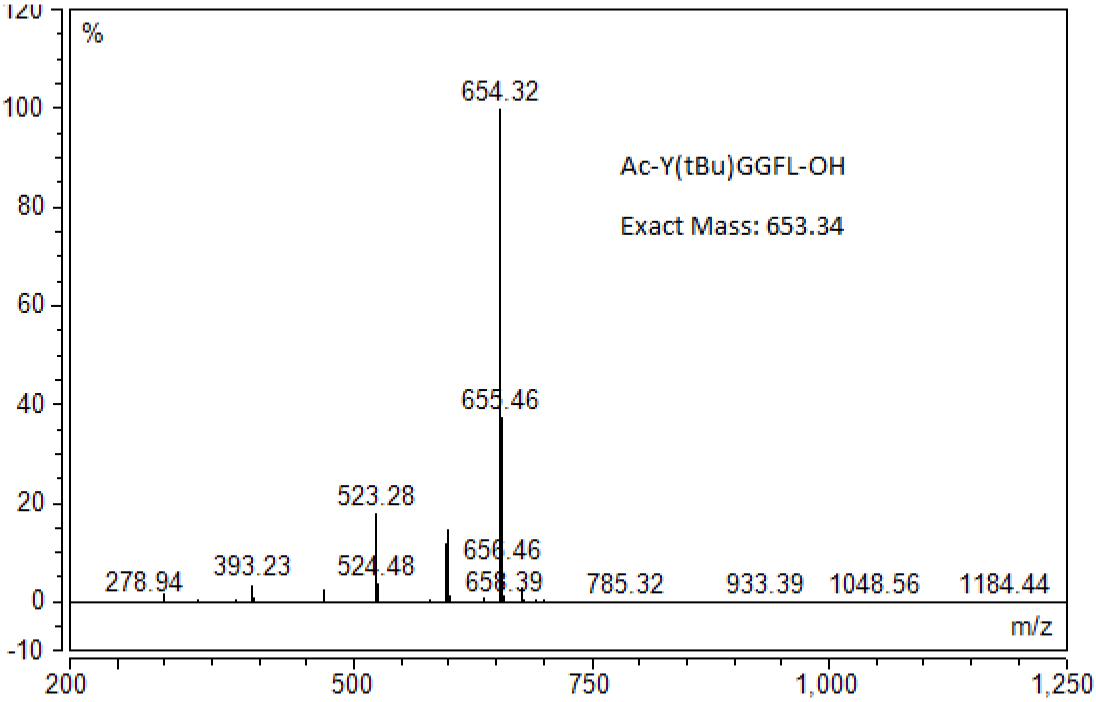

Figure S19. LCMS of Ac-Y(tBu)GGFL-OH, cleavage study using various basic conditions from multi detachable linkers peptide.

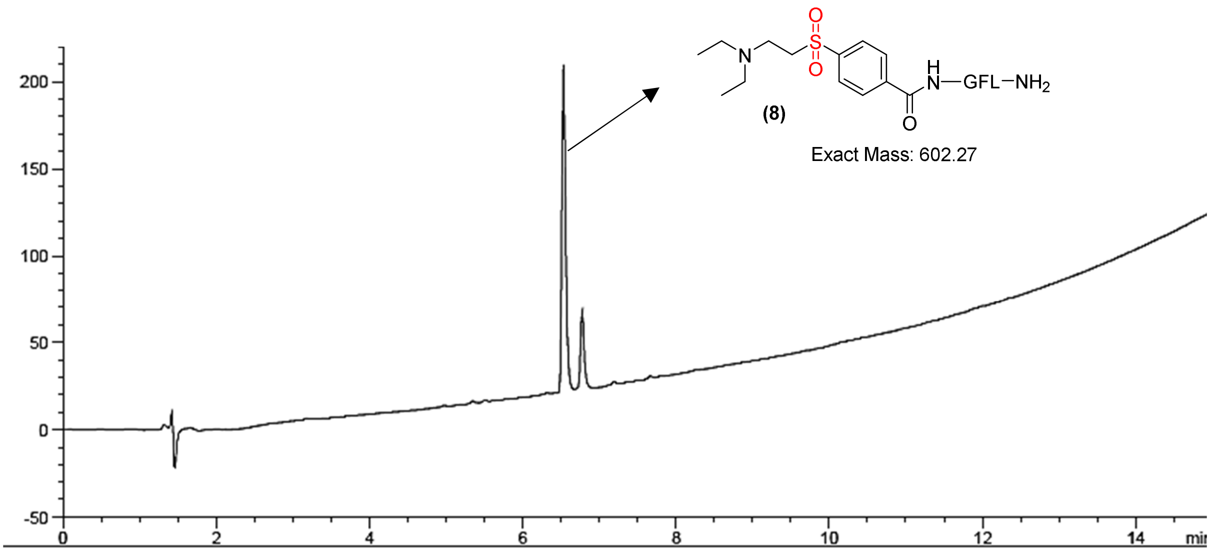

Figure S20. HPLC of Peptide (**8**), with Diethylamine adduct and ETB linker. second cleavage (TFA-TIS-H_20) from multi detachable linkers peptide. Basic condition for the first cleavage (100% DEA, 80% DEA-DCM, 50% DEA-DCM, 20% DEA-DCM, 5%DEA-DCM)

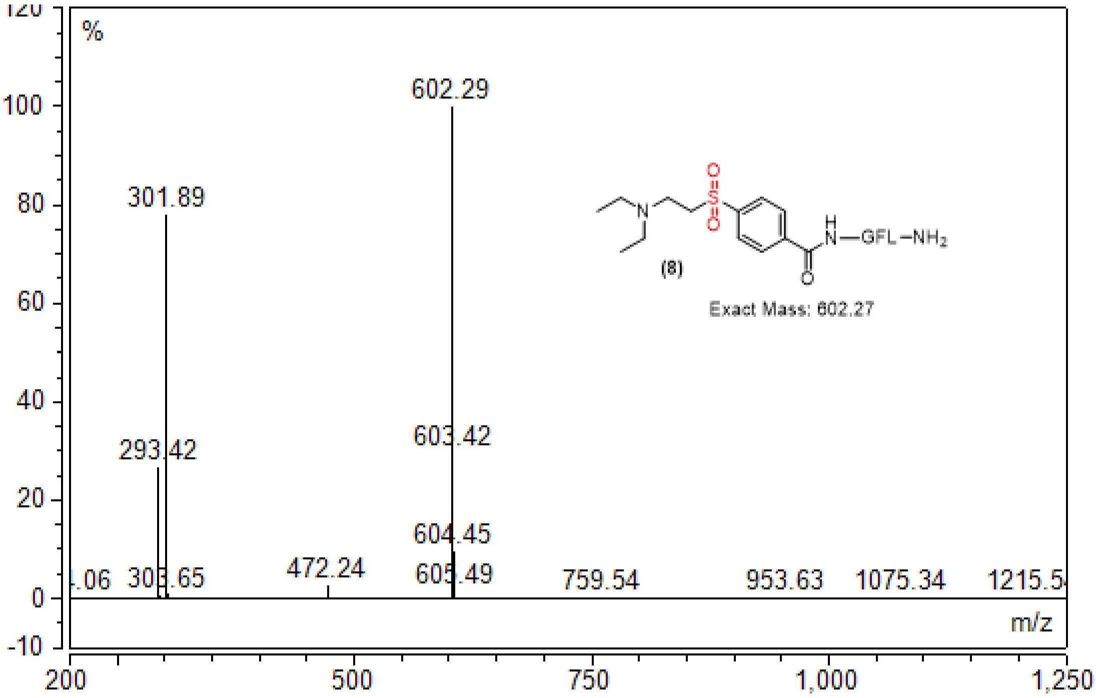

Figure S21. LCMS of Peptide (**8**), with Diethylamine adduct and ETB linker.

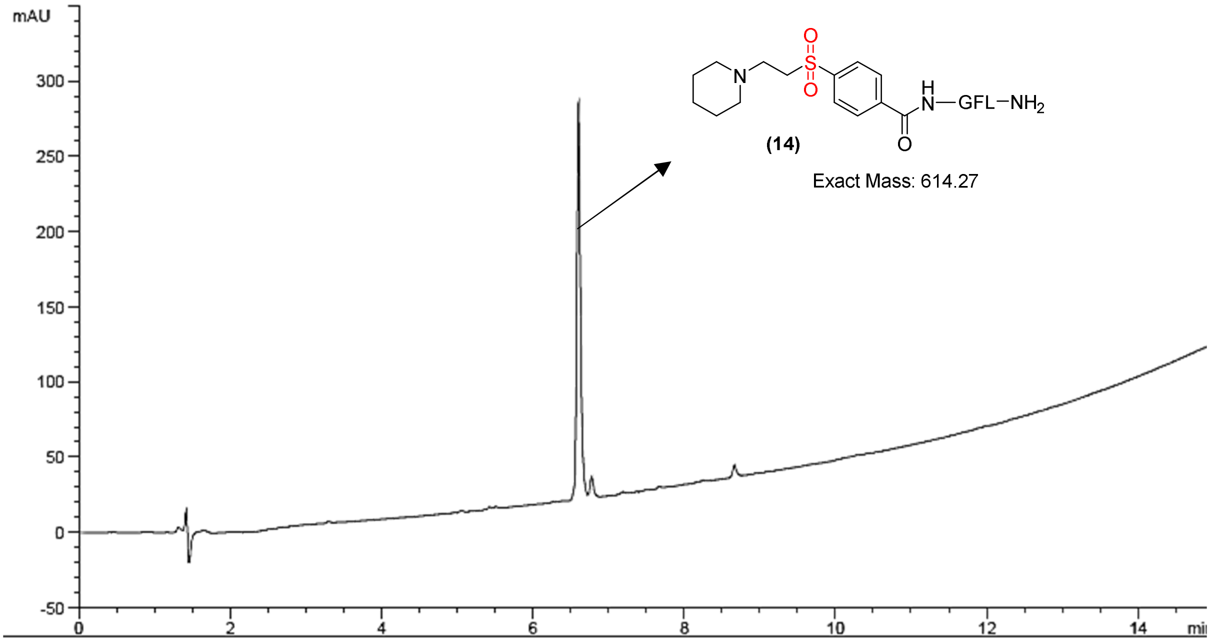

Figure S22. HPLC of Peptide (**14**), with Piperidine adduct and ETB linker. second cleavage (TFA-TIS-H_2O) from multi detachable linkers peptide. Basic condition for the first cleavage (10% Piperidine-DMF)

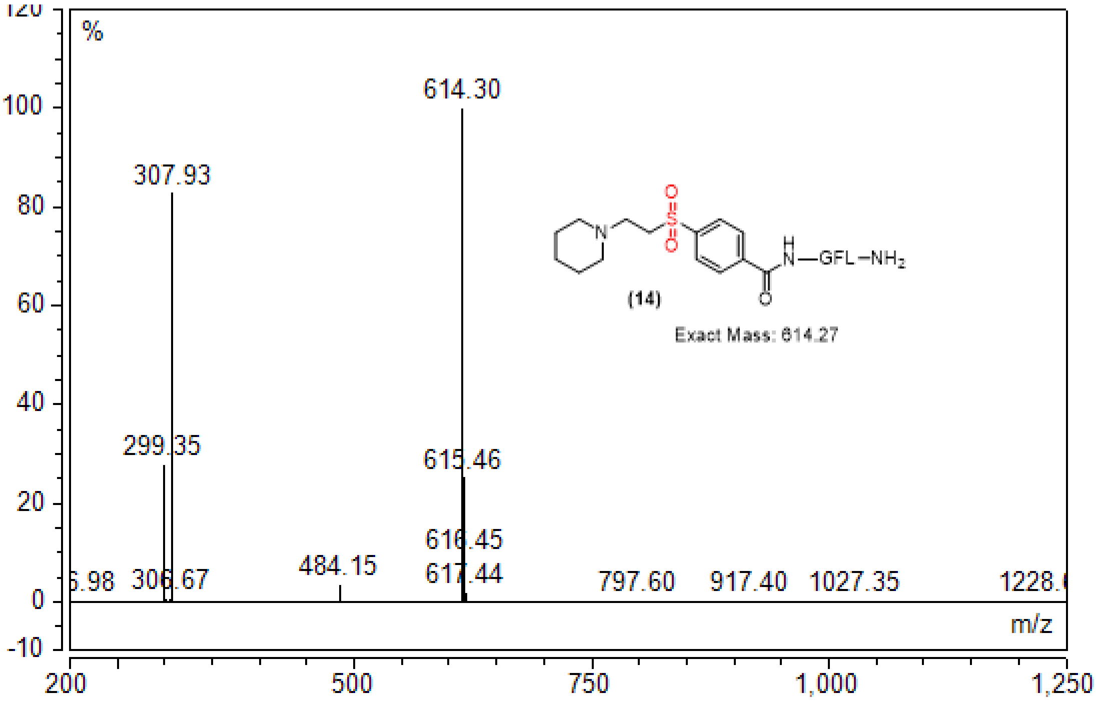

Figure S23. HPLC of Peptide (**14**), with Piperidine adduct and ETB linker.

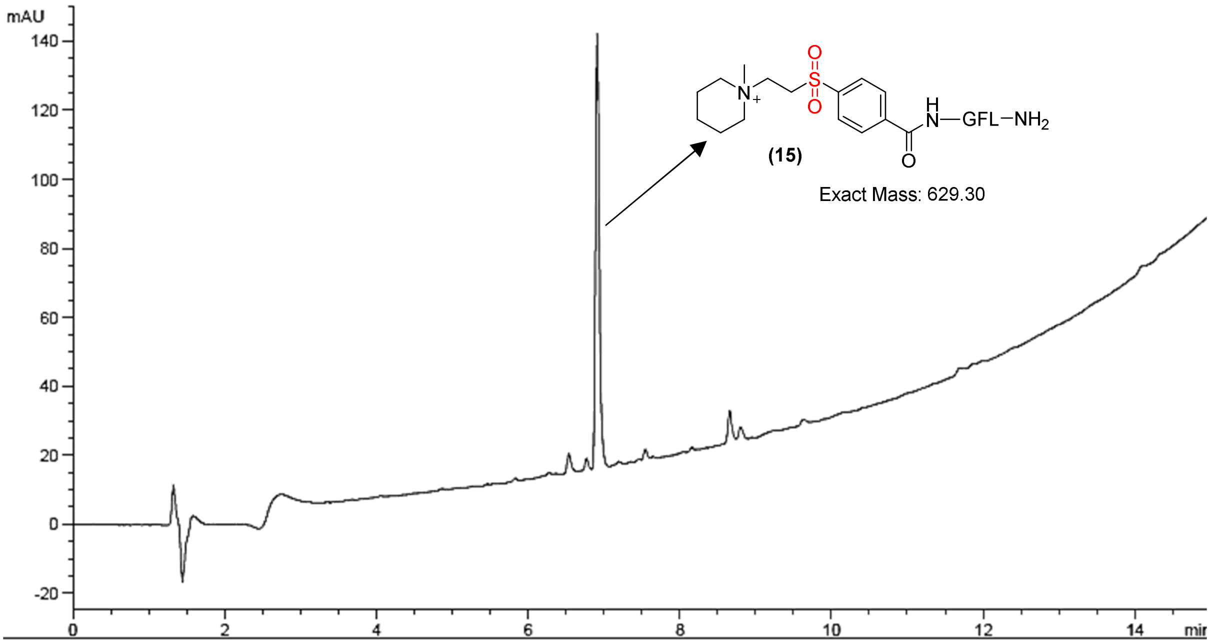

Figure S24. HPLC of Peptide (**15**), with Methylpiperidine adduct and ETB linker. second cleavage (TFA-TIS-H_2O) from multi detachable linkers peptide. Basic condition for the first cleavage (10% Methylpiperidine-DMF)

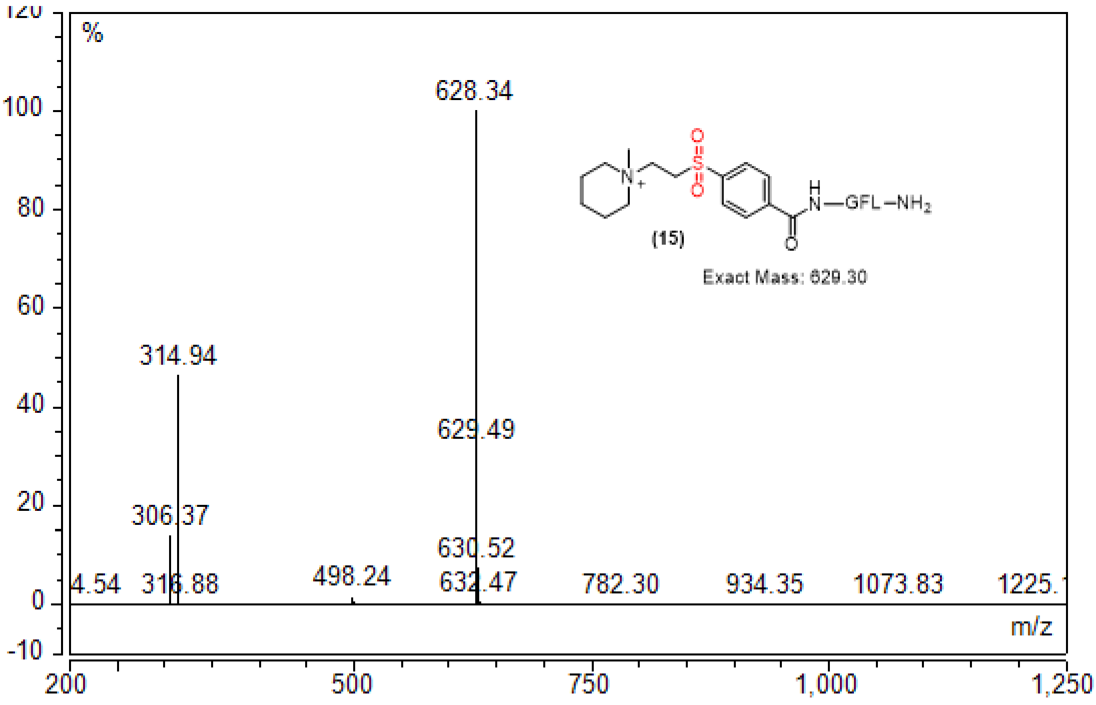

Figure S25. LCMS of Peptide (**15**), with Methylpiperidine adduct and ETB linker.

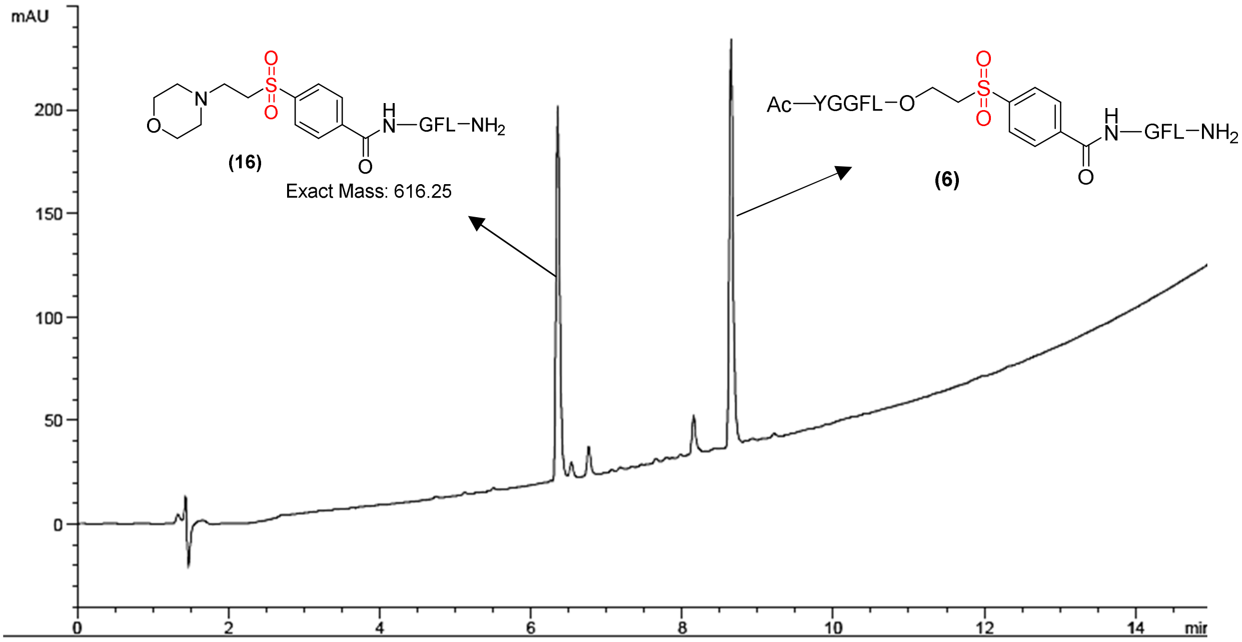

Figure S26. HPLC of Peptide (**16**), with Morpholine adduct and ETB linker. second cleavage (TFA-TIS-H_2O) from multi detachable linkers peptide. Basic condition for the first cleavage (60% Morpholine-DMF)

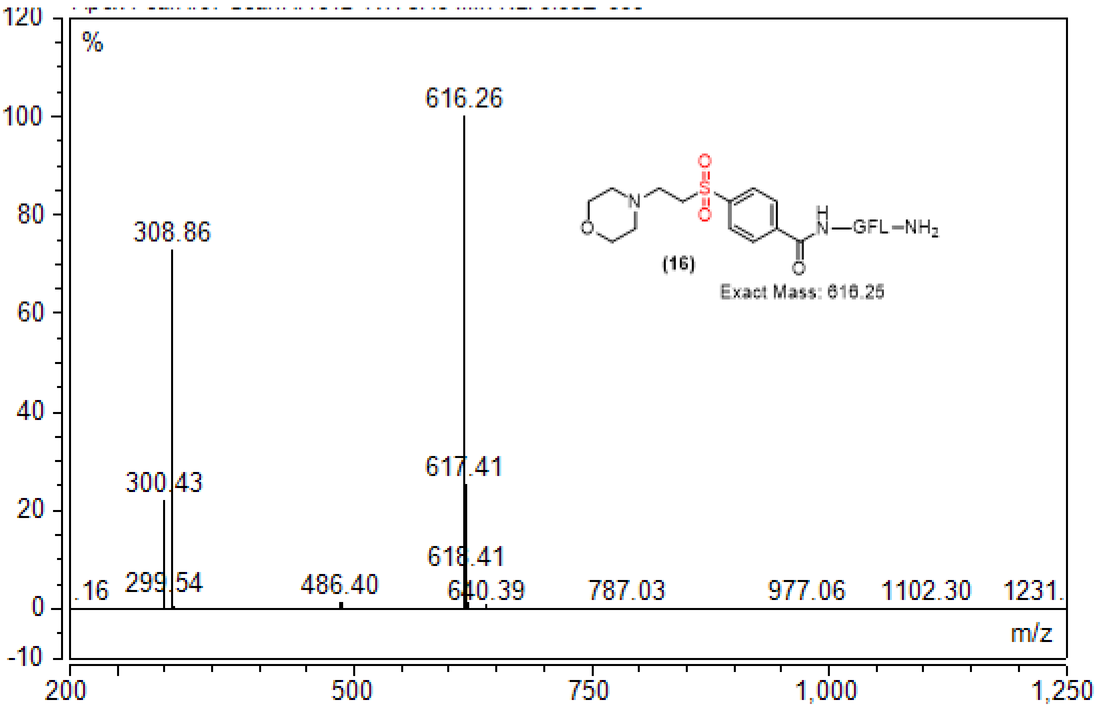

Figure S27. HPLC of Peptide (**16**), with Morpholine adduct and ETB linker.

Synthesis of peptides with sensitive amino acids (Histidine, Tryptophan, and Cysteine) to m-CPBA oxidizing reagent

Peptide (H-His-Gly-Gly-Phe-Leu-OH)

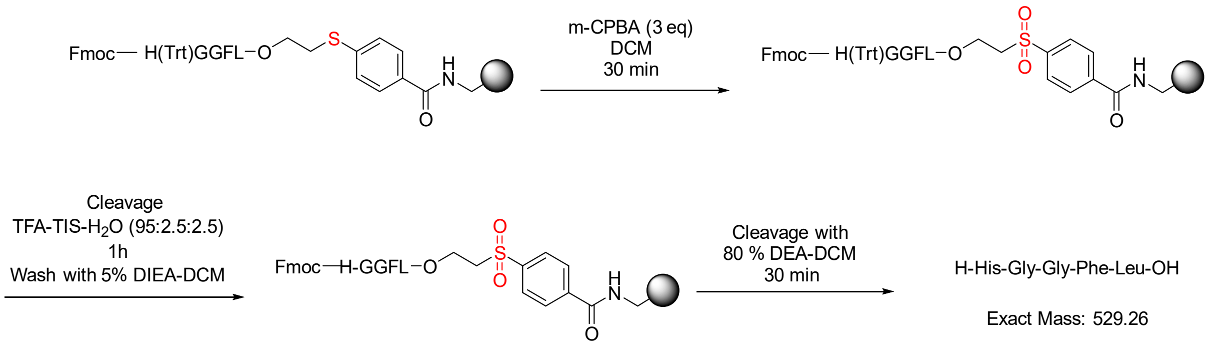

Scheme S4: Synthesis of *H-His-Gly-Gly-Phe-Leu-OH*

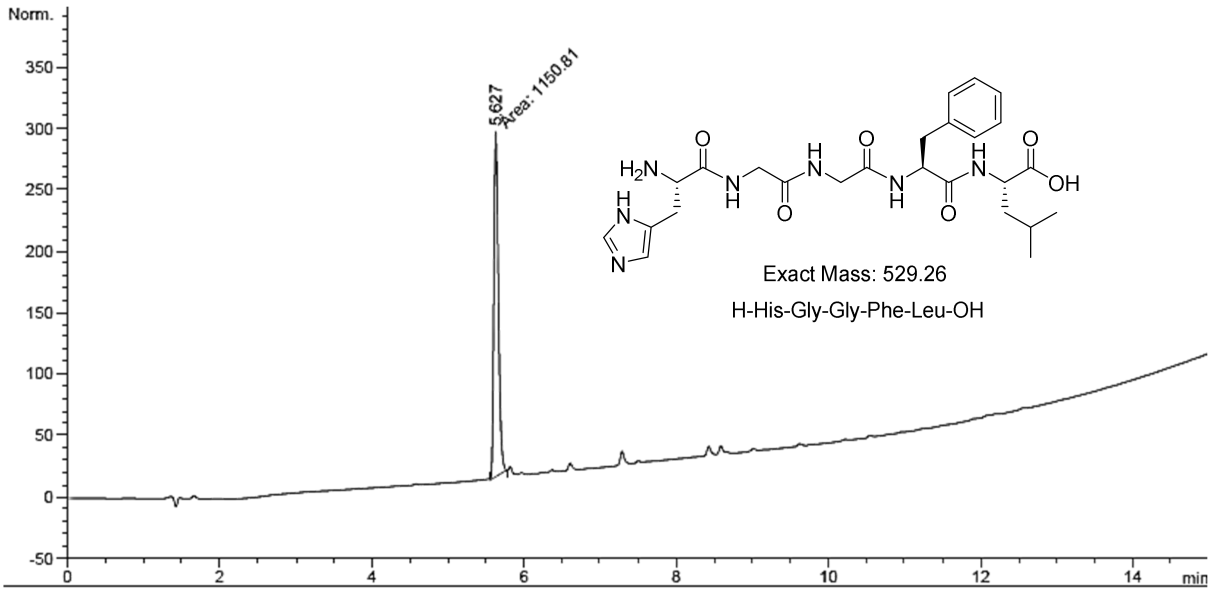

Figure S28. HPLC for peptide **(H-His-Gly-Gly-Phe-Leu-OH)**

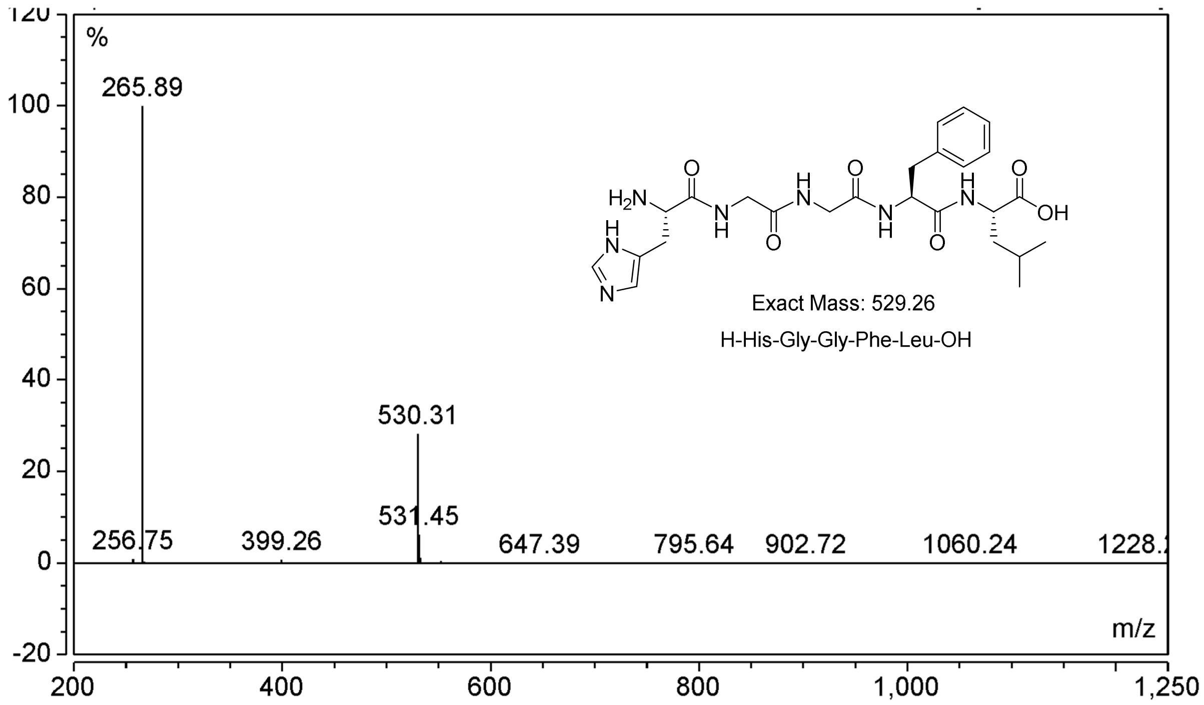

Figure S29. HPLC for peptide **(H-His-Gly-Gly-Phe-Leu-OH)**

Peptide(H-Trp-Gly-Gly-Phe-Leu-OH)

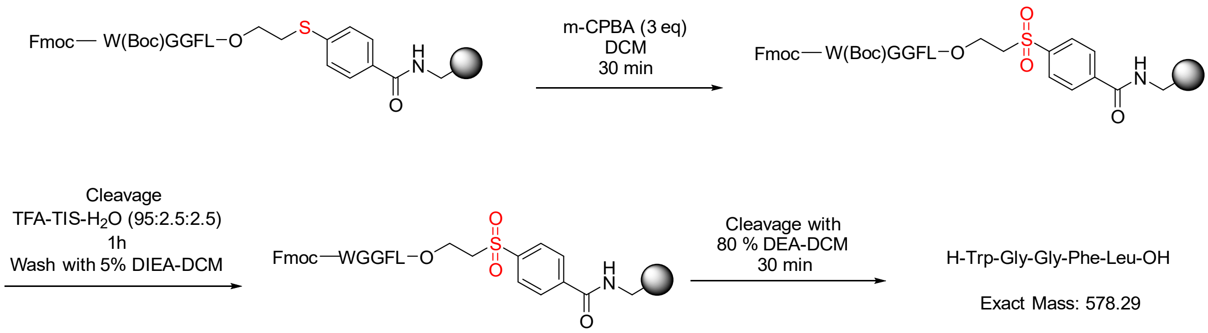

Scheme S5: Synthesis of *H-Trp-Gly-Gly-Phe-Leu-OH*

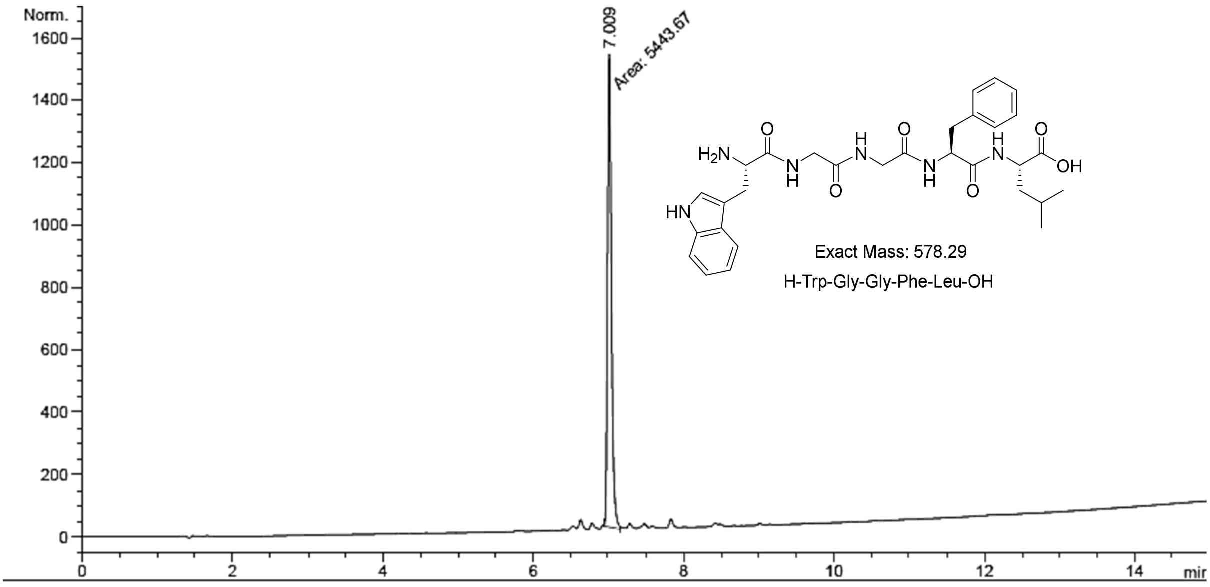

Figure S30. HPLC for peptide **(H-Trp-Gly-Gly-Phe-Leu-OH)**

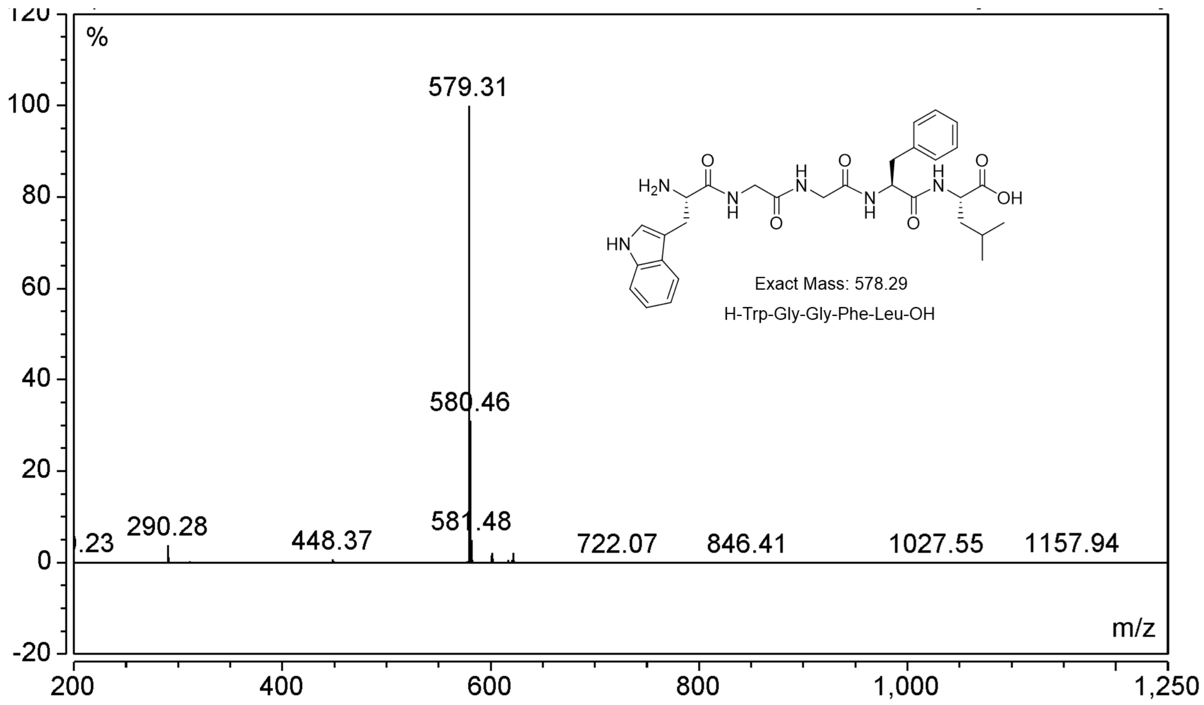

Figure S31. LCMS for peptide **(H-Trp-Gly-Gly-Phe-Leu-OH)**

Peptide(H-Cys-Gly-Gly-Phe-Leu-OH)

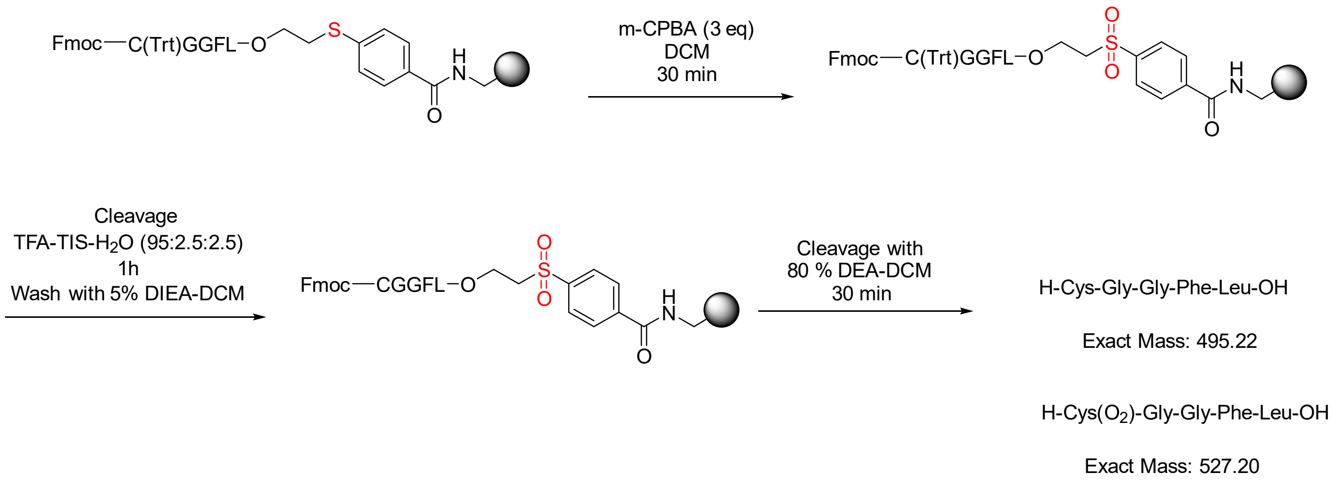

Scheme S6: Synthesis of *H-Cys-Gly-Gly-Phe-Leu-OH*

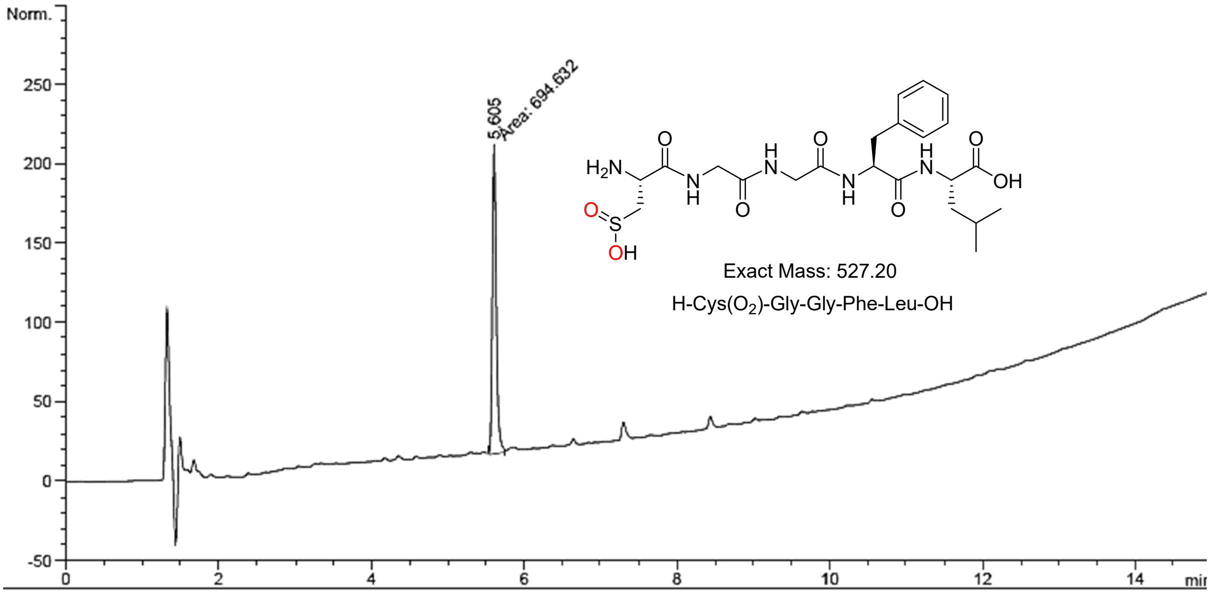

Figure S32. HPLC for peptide **(H-Cys(O_2)-Gly-Gly-Phe-Leu-OH)**

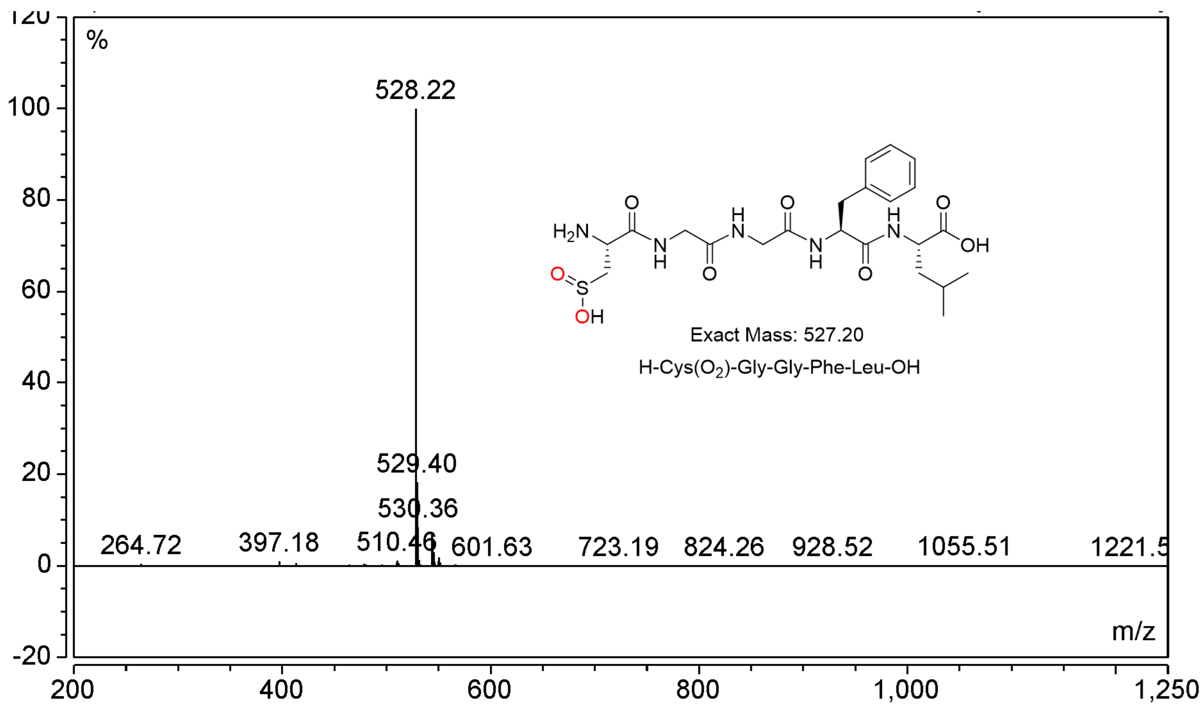

Figure S33. LCMS for peptide **(H-Cys(O_2)-Gly-Gly-Phe-Leu-OH)**

Compatibility of ETB resin with Boc chemistry, minimizing DKP Formation

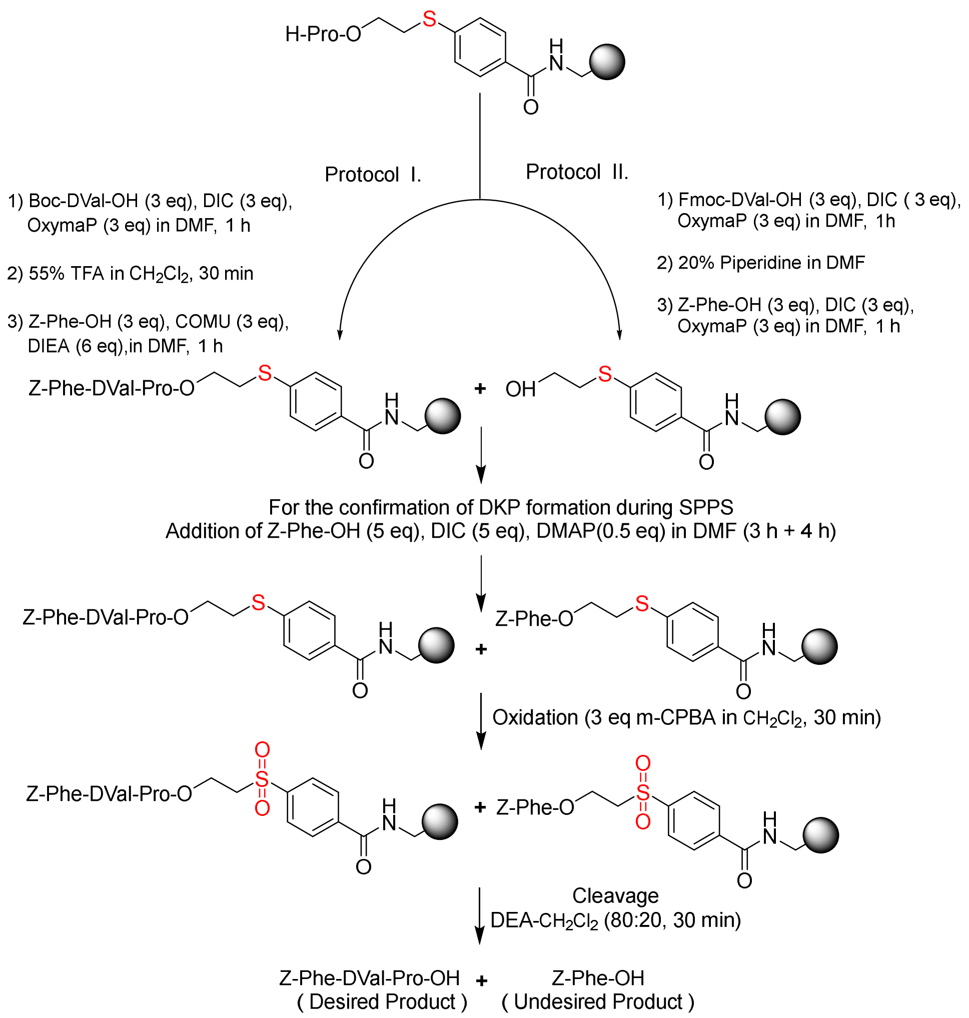

Scheme S7: DKP Formation study on Z-Phe-DVal-Pro-O-ETB Resin

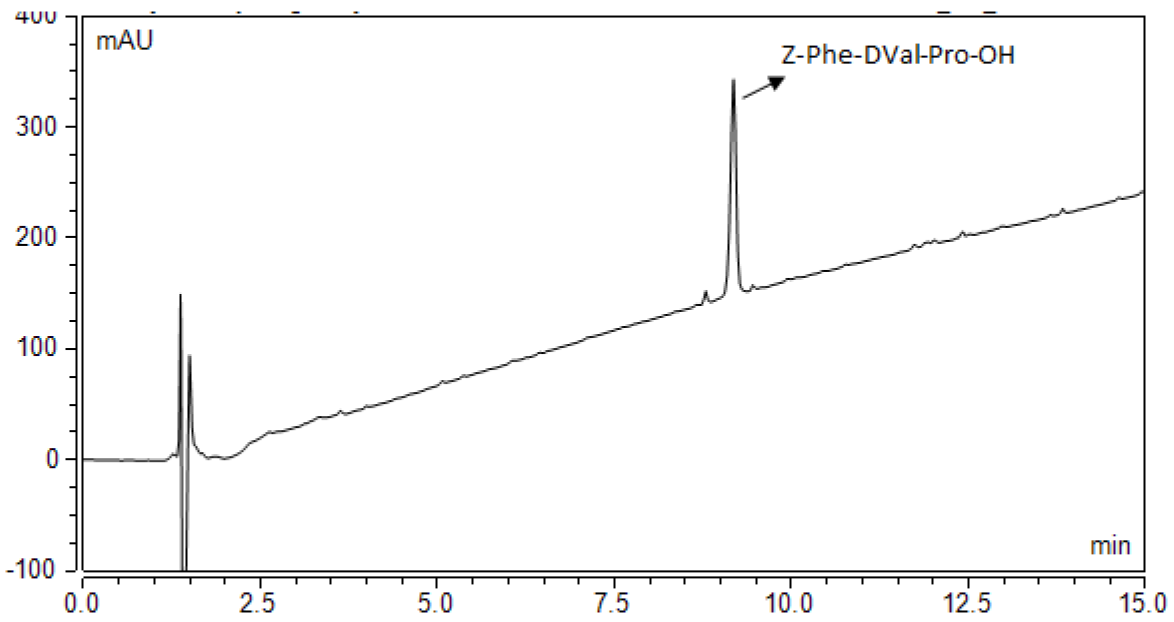

Figure S34: Protocol I: HPLC of Z-Phe-DVal-Pro-OH, before Z-Phe-OH second addition. 0% DKP formation

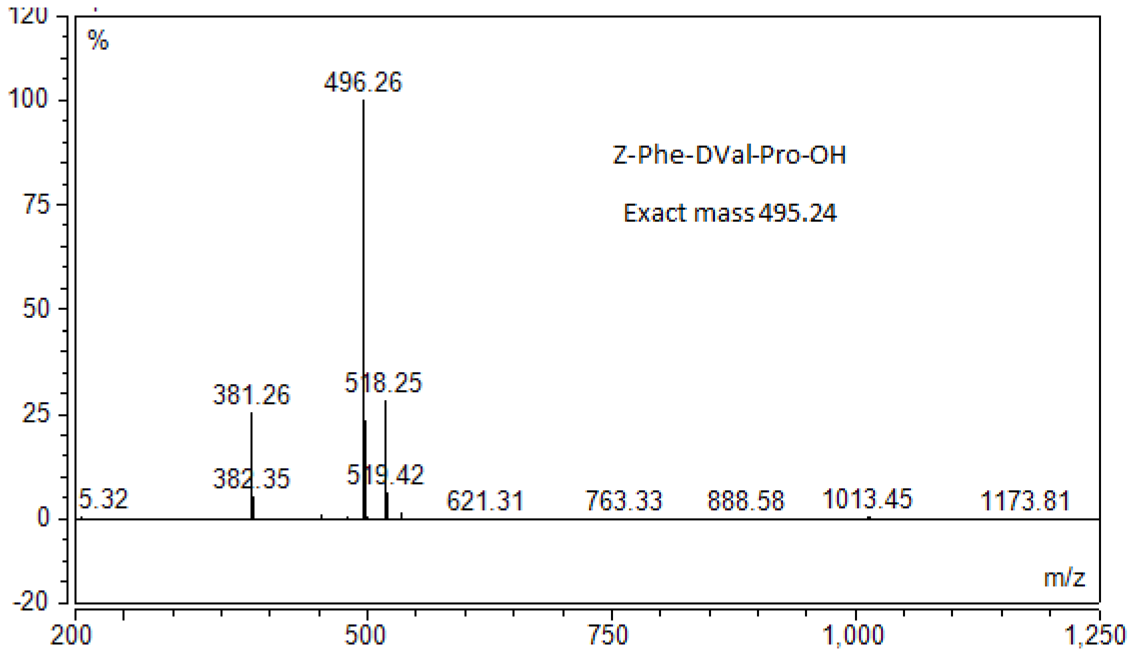

Figure S35: Protocol I: LCMS of Z-Phe-DVal-Pro-OH, **before** Z-Phe-OH second addition.

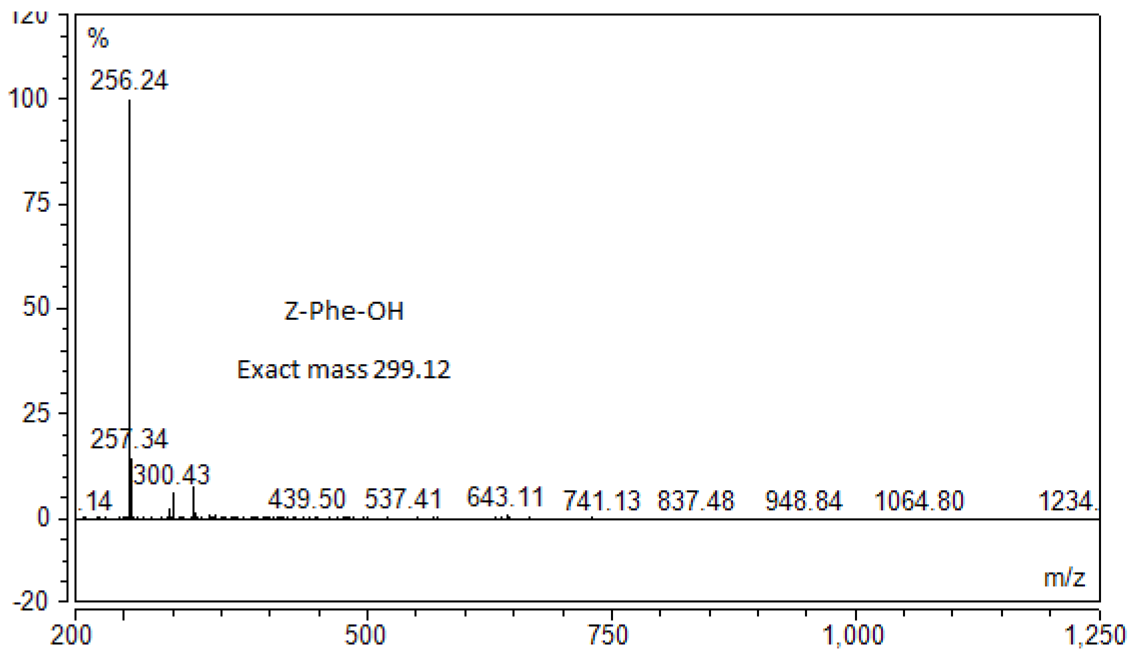

Figure S36: Protocol I: LCMS of Z-Phe-OH, **before** Z-Phe-OH second addition.

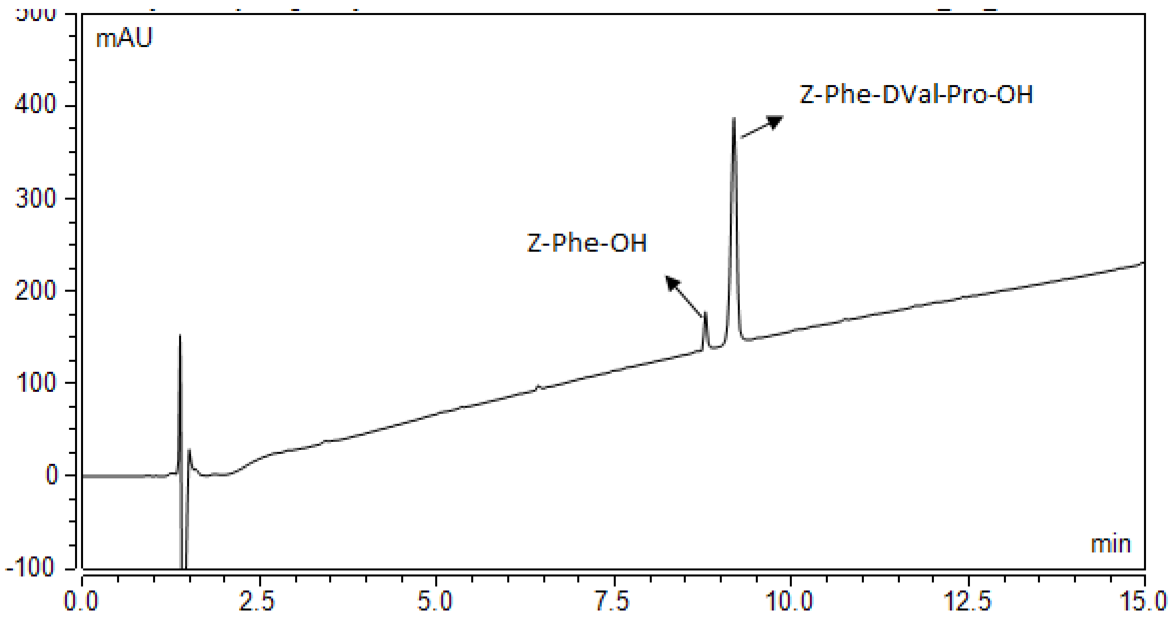

Figure S37: Protocol I: HPLC of Z-Phe-DVal-Pro-OH, **after** Z-Phe-OH second addition.

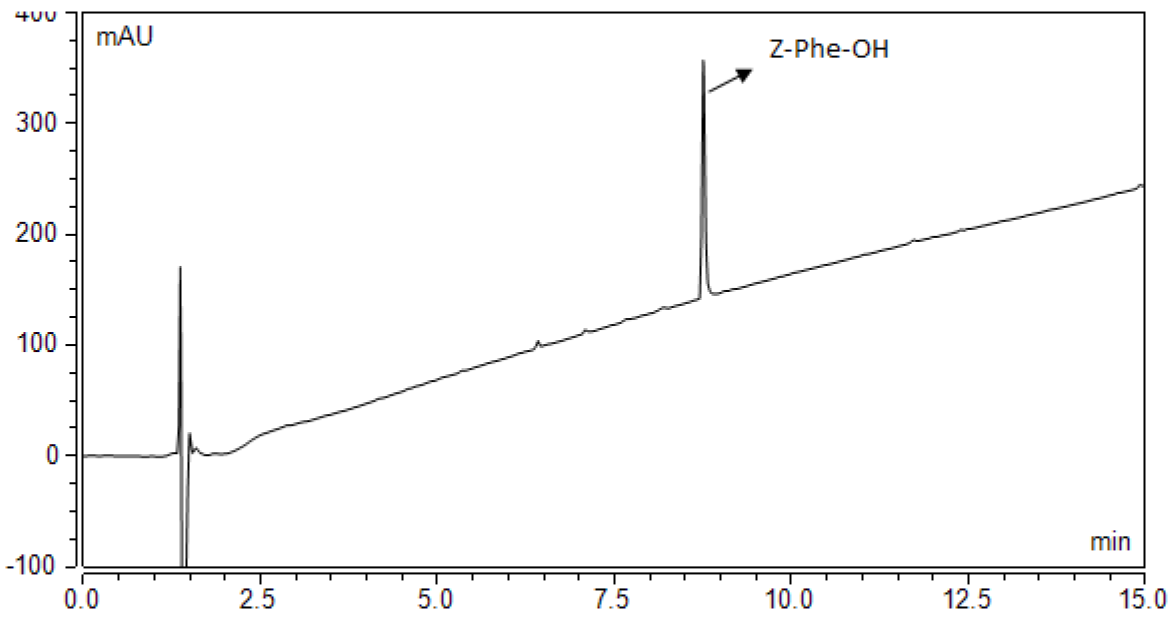

Figure S38: Protocol II: HPLC of Z-Phe-OH, **before** Z-Phe-OH second addition. 100 % DKP formation.

Synthesis of unprotected peptides free of trifluoroacetyl salts

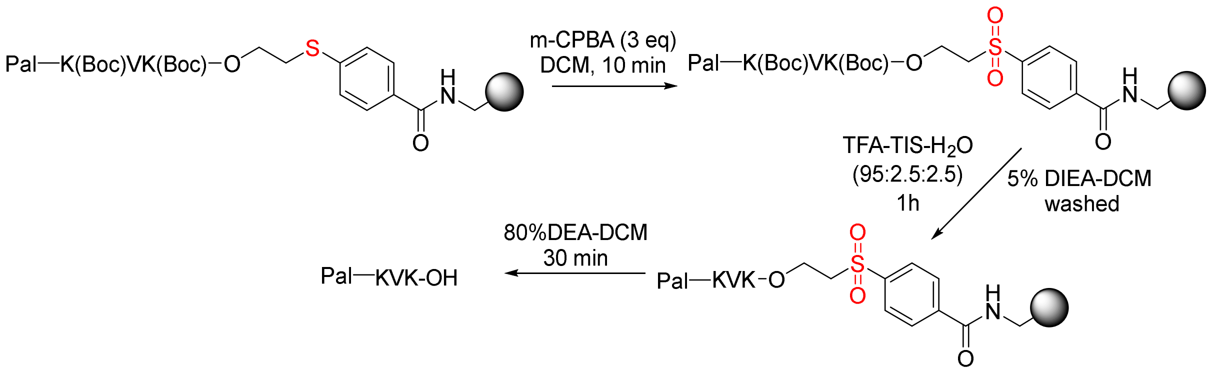

Scheme S8: Synthesis of Palmitoyl tripeptide (Pal-KVK-OH)

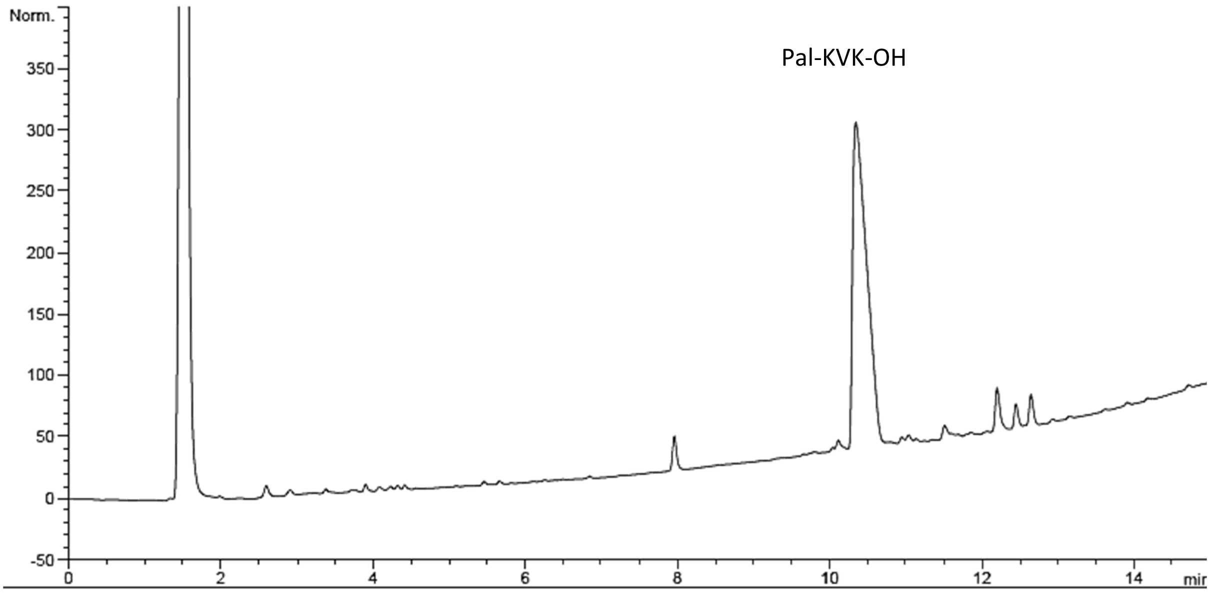

Figure S39: HPLC of Pal-KTTKS-OH

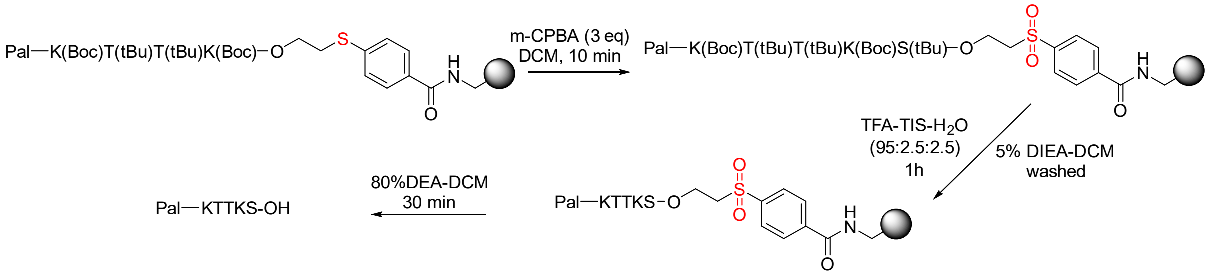

Scheme S9: Synthesis of Palmitoyl pentapeptide (Pal-KVK-OH)

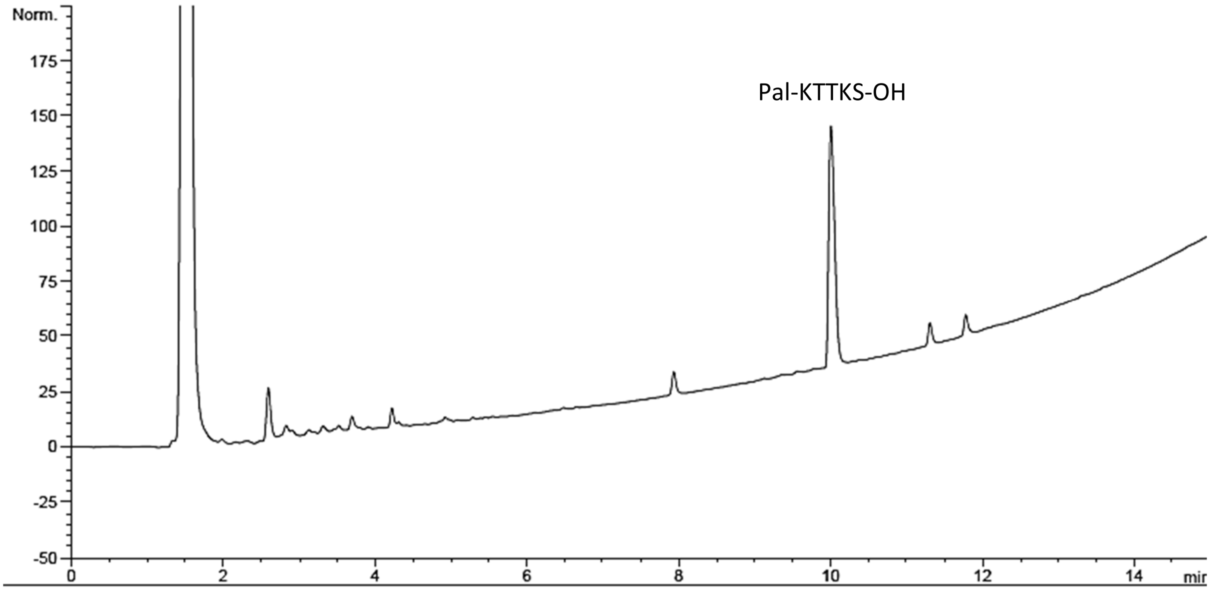

Figure S40: HPLC of Pal-KVK-OH

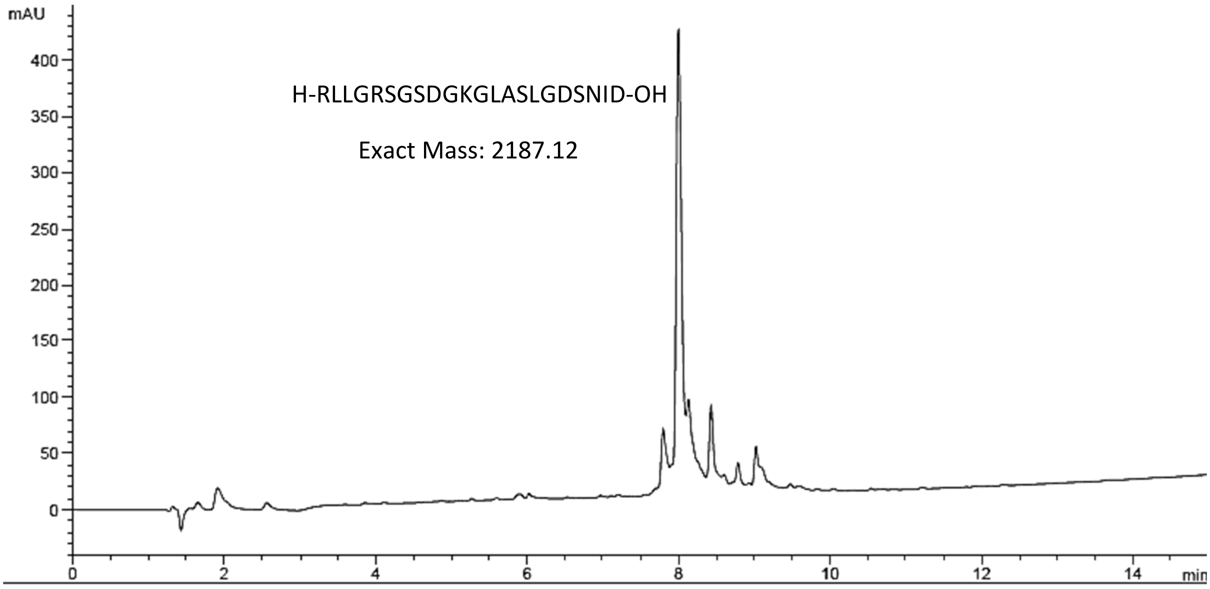

Figure S41: HPLC of peptide-SAK (H-RLLGRSGSDGKGLASLGDSNID-OH) with ETB resin using microwave synthesizer (CEM), 0-60% B (CH_3CN with 0.1% TFA) into A (H_2O with 0.1% TFA)

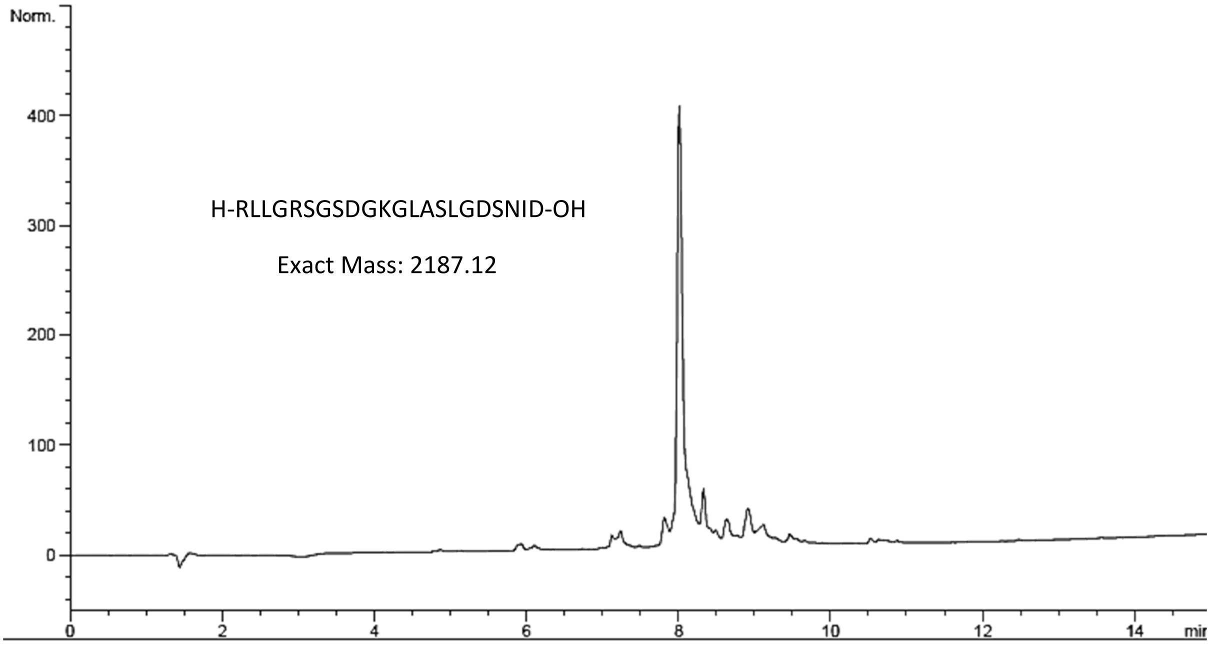

Figure S42: HPLC of peptide-SAK (H-RLLGRSGSDGKGLASLGDSNID-OH) with Wang resin (for reference) using microwave synthesizer (CEM), 0-60% B (CH_3CN with 0.1% TFA) into A (H_2O with 0.1% TFA)

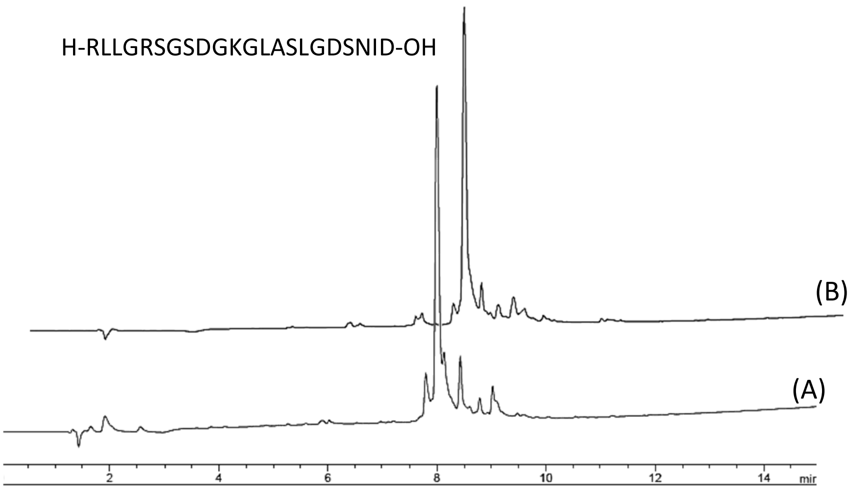

Figure S43: Comparison HPLC of peptide-SAK(H-RLLGRSGSDGKGLASLGDSNID-OH) with ETB resin (A) and Wang resin (B) (for reference) using microwave synthesizer (CEM), 0-60% B (CH_3CN with 0.1% TFA) into A (H_2O with 0.1% TFA)

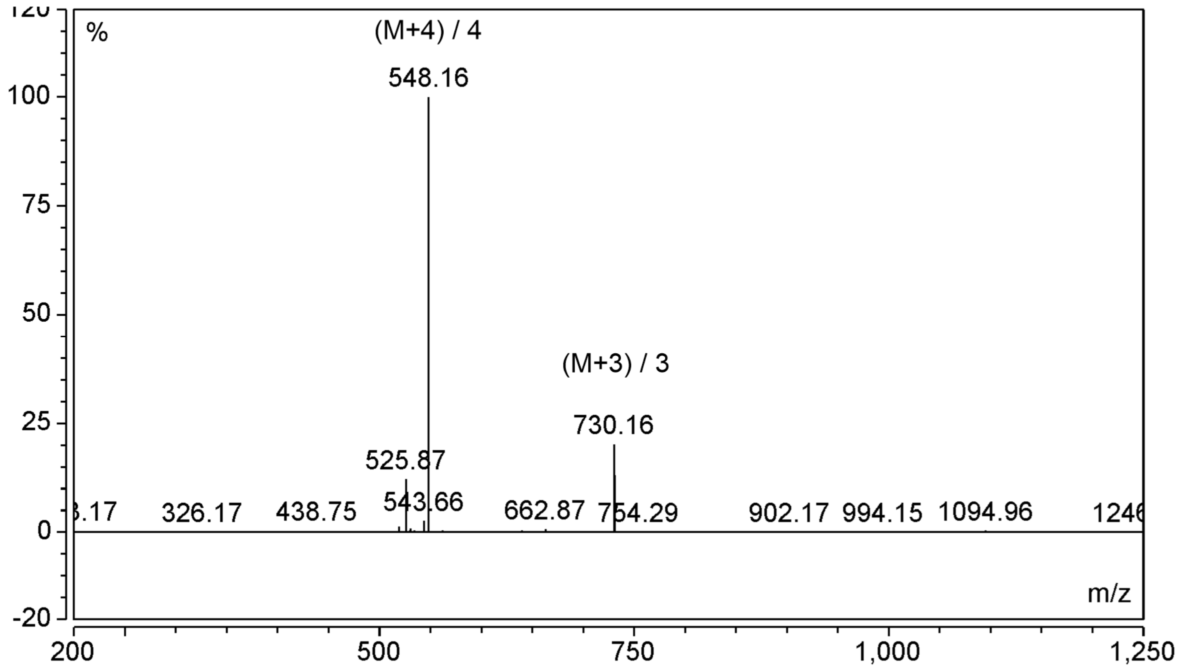

Figure S44: LCMS of peptide-SAK (H-RLLGRSGSDGKGLASLGDSNID-OH)

Synthesis and characterisation of ETB Linker 2

Noki Linker 2

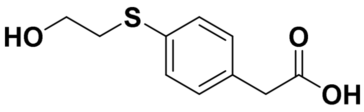

Exact Mass: 212,0507

Preparation of methyl 2-(4-mercaptophenyl) acetate

A mixture of 2-(4-mercaptophenyl) acetic acid (5.0 g), H_2SO_4 (0.063 mL) in methanol (20 mL) was heated to 70°C with stirring for 2 hours, the reaction mixture was monitoring by TLC (SiO_2) (MeOH/EtOAc, 5: 95). The reaction mixture was cooled to 0°C in ice bath, and then neutralized with $NaHCO_3$ to pH = 7. Solvent was concentrated to about 10 mL. Then DCM (30 mL) and water (20 mL) was added. The aqueous phase was extracted with DCM (20 mL x 3). The combined organics were dried over $MgSO_4$, filtered, and concentrated to render methyl 2-(4-mercaptophenyl) acetate (4.5 g). (90% yield).

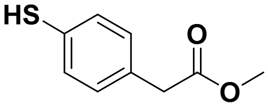

HPLC [5-95% of MeCN (0.1% TFA/ H_2O (0.1% TFA) over 15min] tR = 8.463 min. **^{1}H NMR** (600MHz, DMSO): δ=7.24 (d, *J*= 7.74 Hz, 2H; ArH), 7.13 (d, *J*= 7.75Hz, 3.62 (s, 2H, CH_2), 3.60 (s, 3H, CH_3), **^{13}C{^{1}H}NMR** (150 MHz, DMSO): 171.7, 130.0, 129.7, 127.8, 52.0, 40.6. **HRMS**: m/z: calcd. for $C_9H_9O_2S^-$: 181.0329 $[M\text{-}H]^-$; found: 181.0325

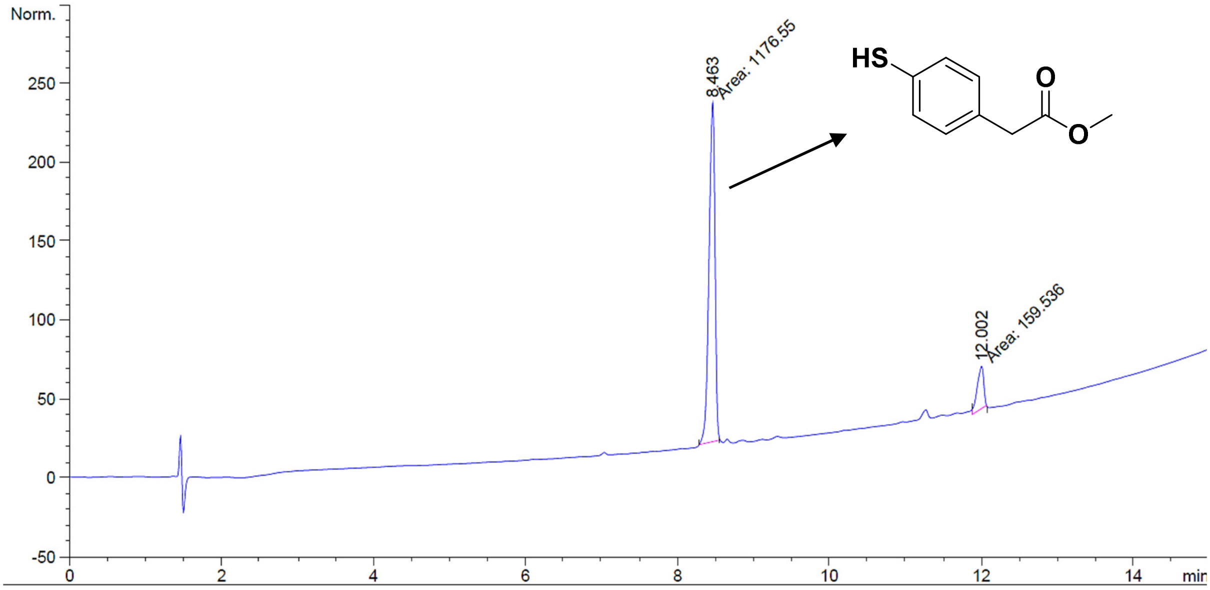

Figure S45: HPLC for methyl 2-(4-mercaptophenyl) acetate

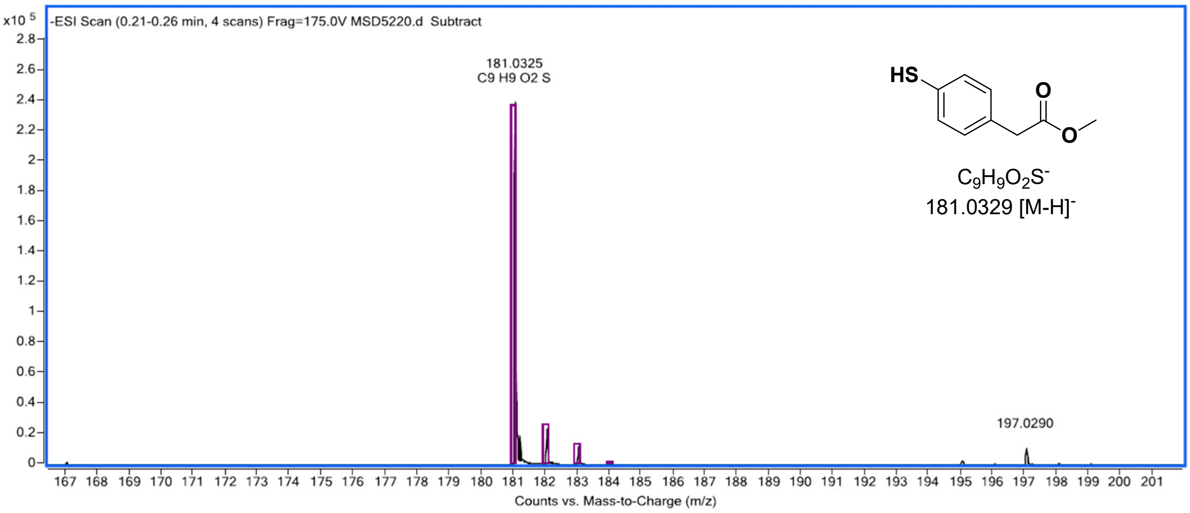

Figure S46: HRMS for methyl 2-(4-mercaptophenyl) acetate

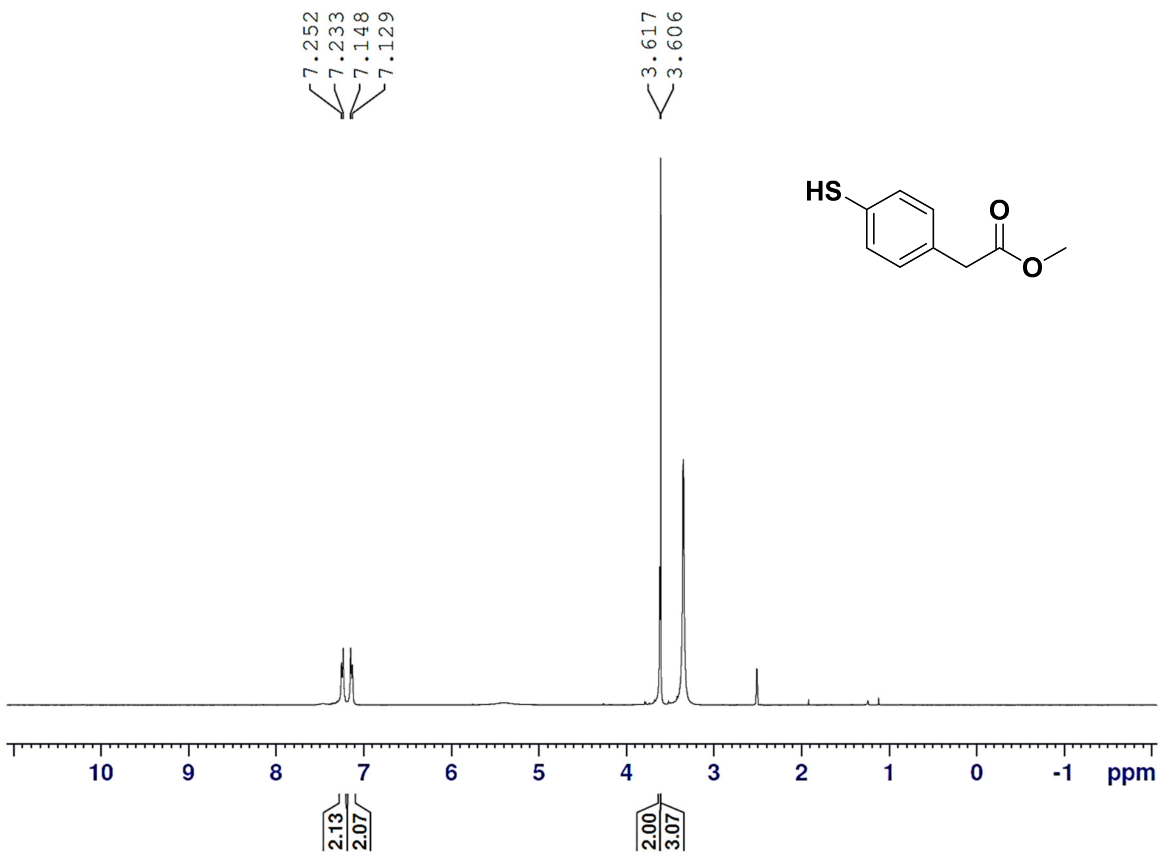

Figure S47: ^{1}H NMR for methyl 2-(4-mercaptophenyl) acetate

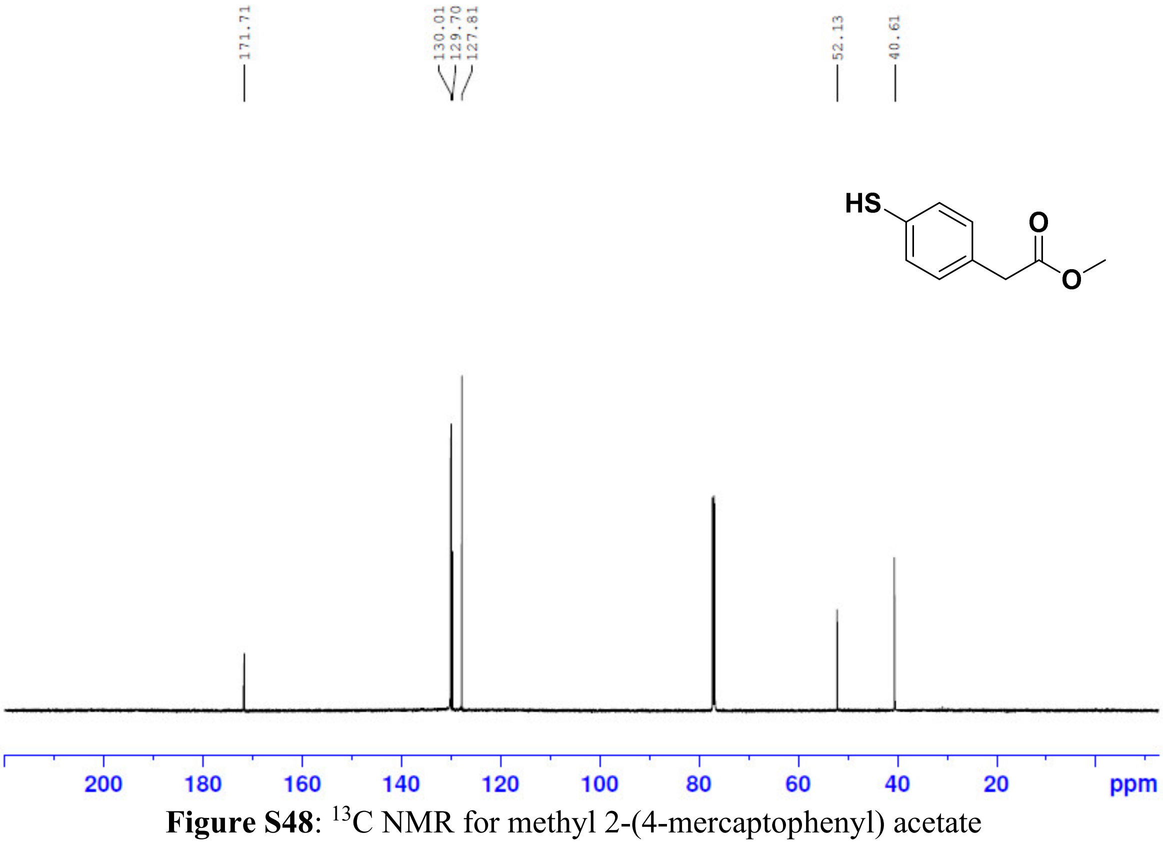

Figure S48: ^{13}C NMR for methyl 2-(4-mercaptophenyl) acetate

Preparation of methyl 2-(4-((2-hydroxyethyl) thio) phenyl acetate

A mixture of methyl 2-(4-mercaptophenyl) acetate (4.5 g), 2-bromoethanol (2.5 g) and Cs_2CO_3 (3.10 g) in N, N - dimethylformamide (DMF) (70 mL) was stirred at room temperature overnight. The mixture was filtered, and DCM (70 mL) was added. The solution was washed with water (50 mL x 5), brine (50 mL x 2), dried over MgSO4 filtered, and concentrated. The crude product was purified by flash chromatography (silica gel; PE: EA = 20:1 to 2:1), to render methyl 2-(4-((2-hydroxyethyl) thio) phenyl acetate (**3)** (4.g) as a white solid. (91% yield).

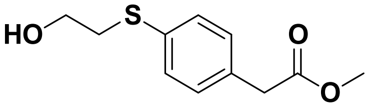

HPLC [5-95% of MeCN (0.1% TFA/ H_2O (0.1% TFA) over 15min tR = 11.501 ^{1}**H NMR** (600MHz, $CDCl_3$): δ=7.33 (d, *J*= 8.2 Hz, 2H; ArH), 7.20 (d, *J*= 8.1 Hz, 3.73 (t, *J*= 6.2 Hz, 2H), 3.68 (s, 2H), 3.59 (s, 3H), 3.08 (t, *J*= 6.2 Hz, 2H) ^{13}**C{**^{1}**H}NMR** (150 MHz, $CDCl_3$): 171.8, 133.9, 132.4, 130.2, 129.9, 60.4, 52.1, 40.6, 36.9. **HRMS**: m/z: calcd. for $C_{11}H_{15}O_3S^+$: 227.0736 $[M+H]^+$; found: 227.0741

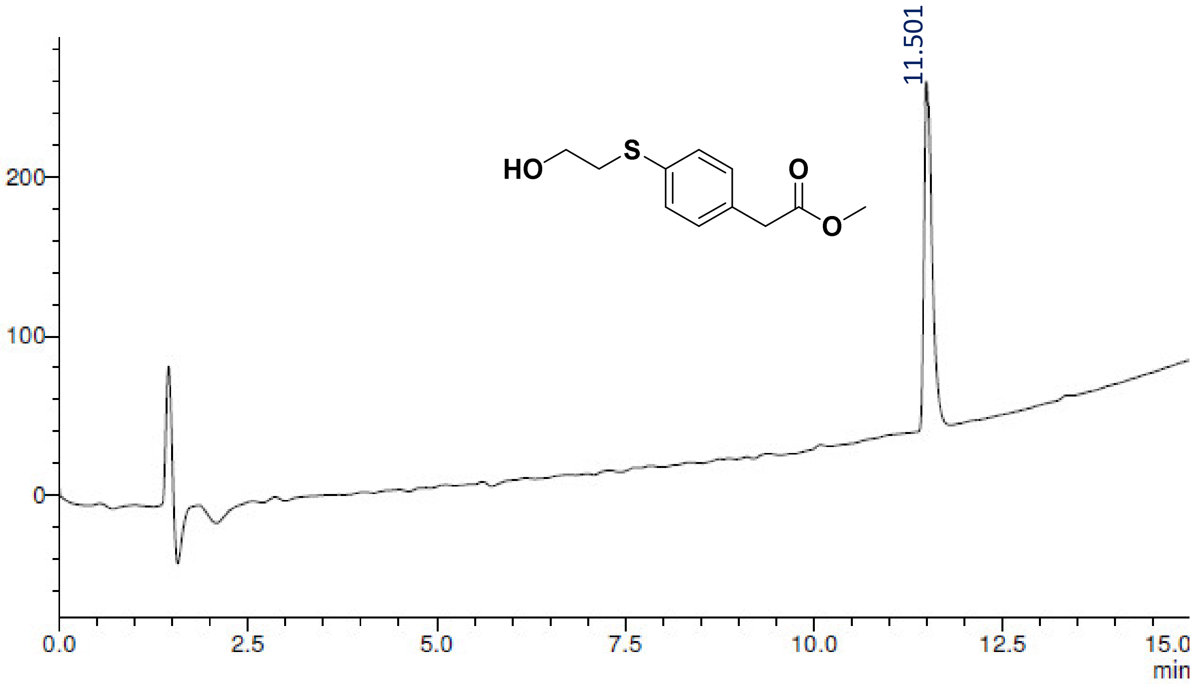

Figure S49: HPLC for methyl 2-(4-((2-hydroxyethyl) thio) phenyl acetate

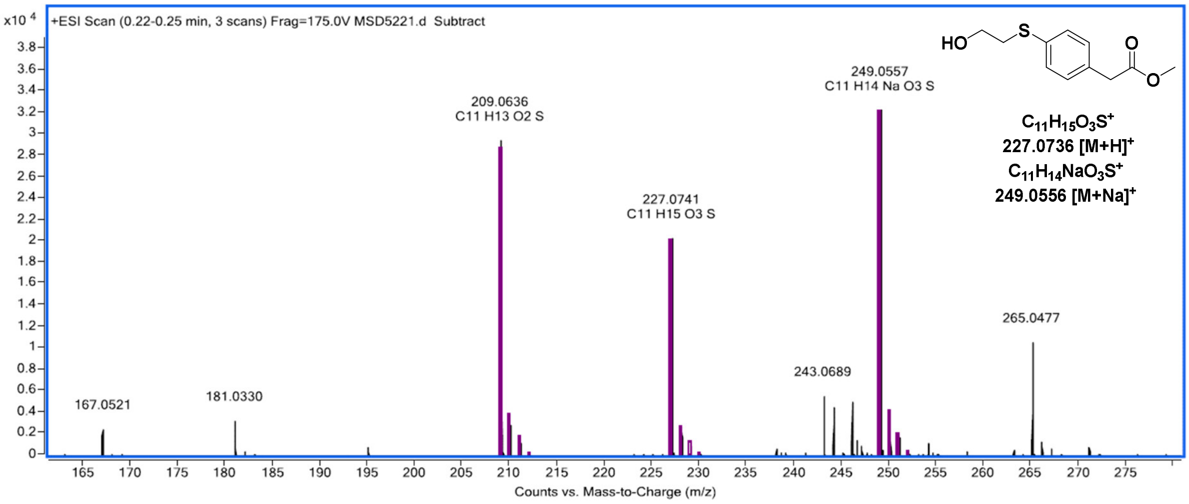

Figure S50: HRMS for methyl 2-(4-((2-hydroxyethyl) thio) phenyl acetate

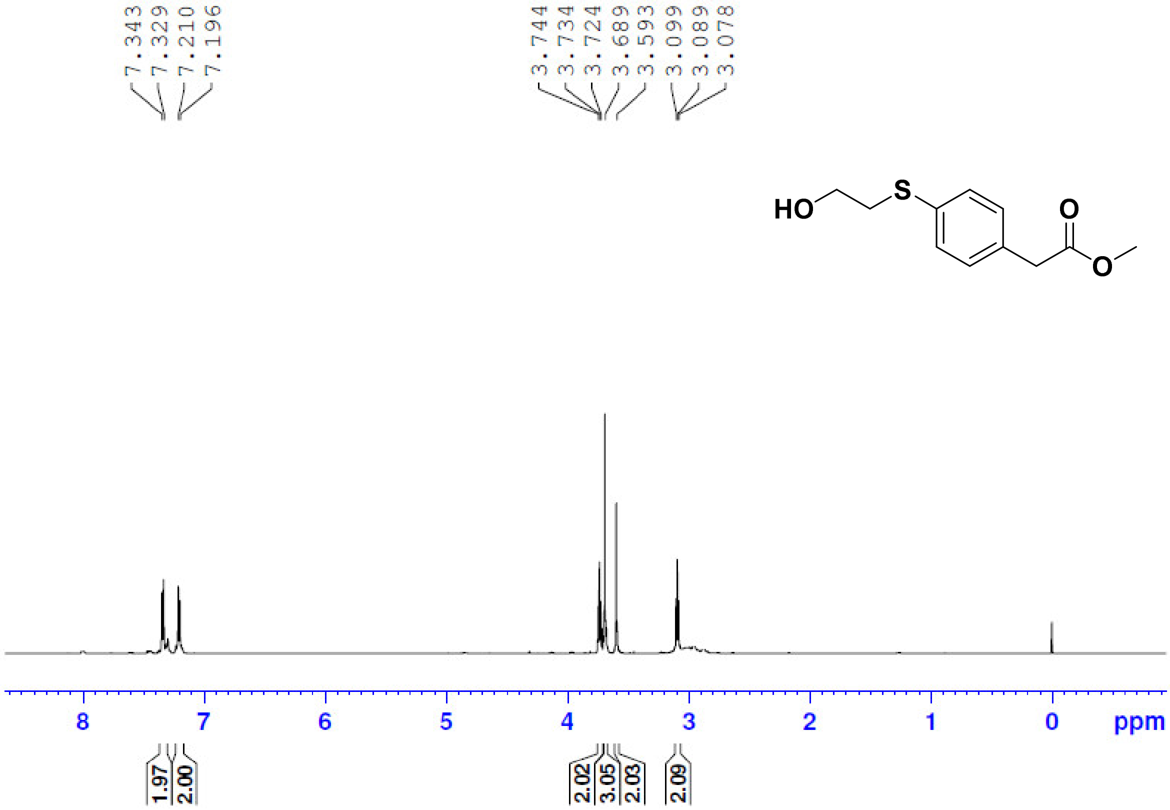

Figure S51: ^{1}H NMR for methyl 2-(4-((2-hydroxyethyl) thio) phenyl acetate

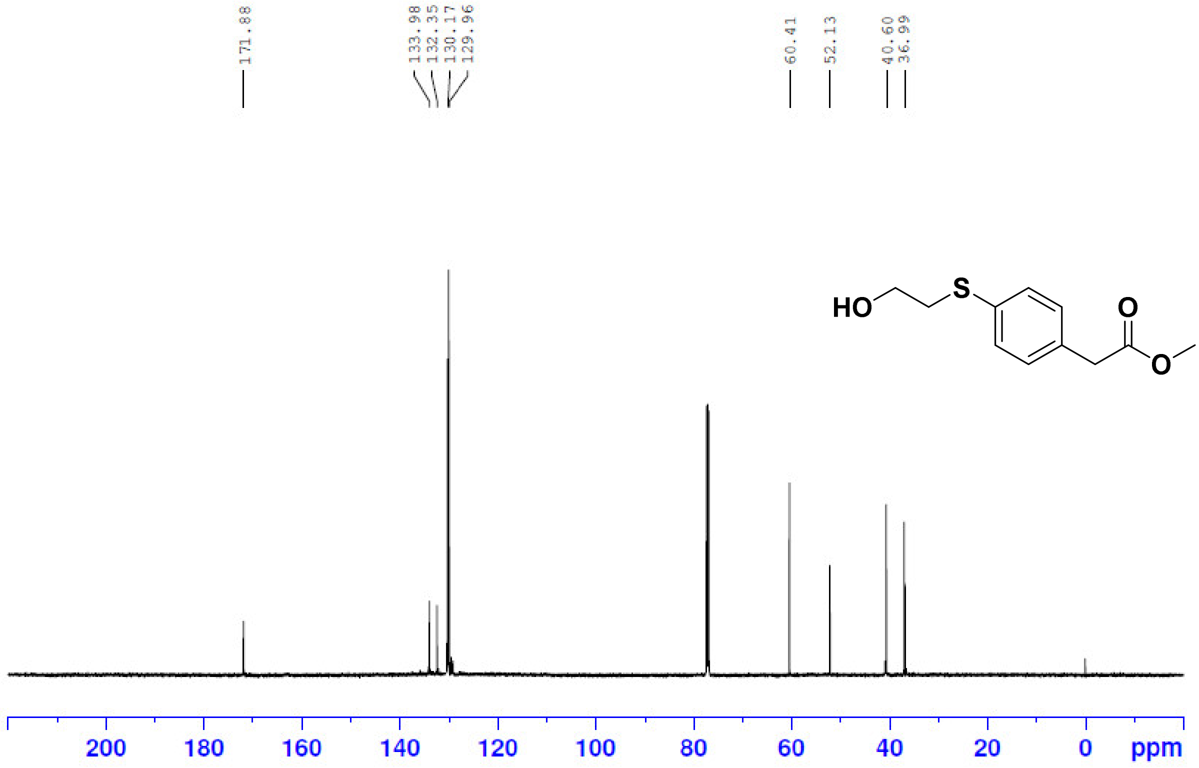

Figure S52: ^{13}C NMR for methyl 2-(4-((2-hydroxyethyl) thio) phenyl acetate

Preparation of 2-(4-((2-hydroxyethyl) thio) phenyl acetic acid

Lithium hydroxide monohydrate (1.4 g) in water (70 mL) was added into a solution of methyl 2-(4-((2-hydroxyethyl) thio) phenyl) acetate (**3**) (4.1 g) in tetrahydrofuran (THF) (70.0 mL). The reaction mixture was stirred at room temperature overnight. Solvent was removed under reduced pressure, and the mixture was cooled in an ice bath and acidified to pH = 1-2 with conc. HCl. The aqueous phase was extracted with EtOAc (50 mL x 5). The combined organic phases were washed with brine (50 mL x 2), dried over $MgSO_4$, filtered, and concentrated, to render 2-(4-((2-hydroxyethyl) thio) phenyl acetic acid (3.6 g) as a white solid. (90% yield).

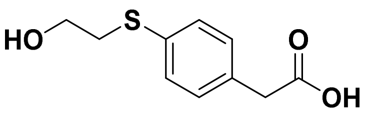

HPLC [5-95% of MeCN (0.1% TFA/ H_2O (0.1% TFA) over 15min tR = 5.049. ^{1}**H NMR** (600MHz, DMSO): δ=12.36 (s, 1H), 7.29 (d, *J*= 8.2 Hz, 2H; ArH), 7.21 (d, *J*= 8.3 Hz, 2H, ArH), 3.56 (t, *J*= 6.9 Hz, 2H), 3.54 (s, 2H), 3.02 (t, *J*= 6.9 Hz, 2H). ^{13}**C{**^{1}**H} NMR** (150 MHz, DMSO): 173.1, 134.8, 132.9, 130.5, 128.5, 60.3, 40.5, 35.5. **HRMS**: m/z: calcd. for $C_{10}H_{11}O_3S^-$: 211.0434 [M-H]$^-$; found: 211.0440.

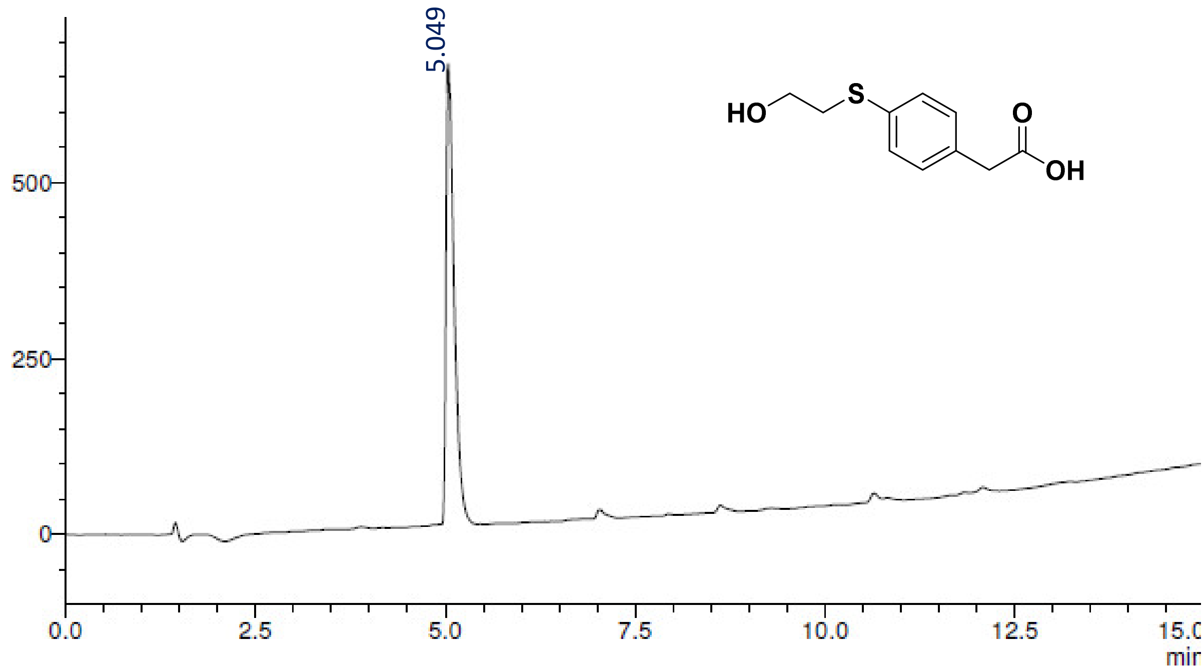

Figure S53: HPLC for 2-(4-((2-hydroxyethyl) thio) phenyl acetic acid

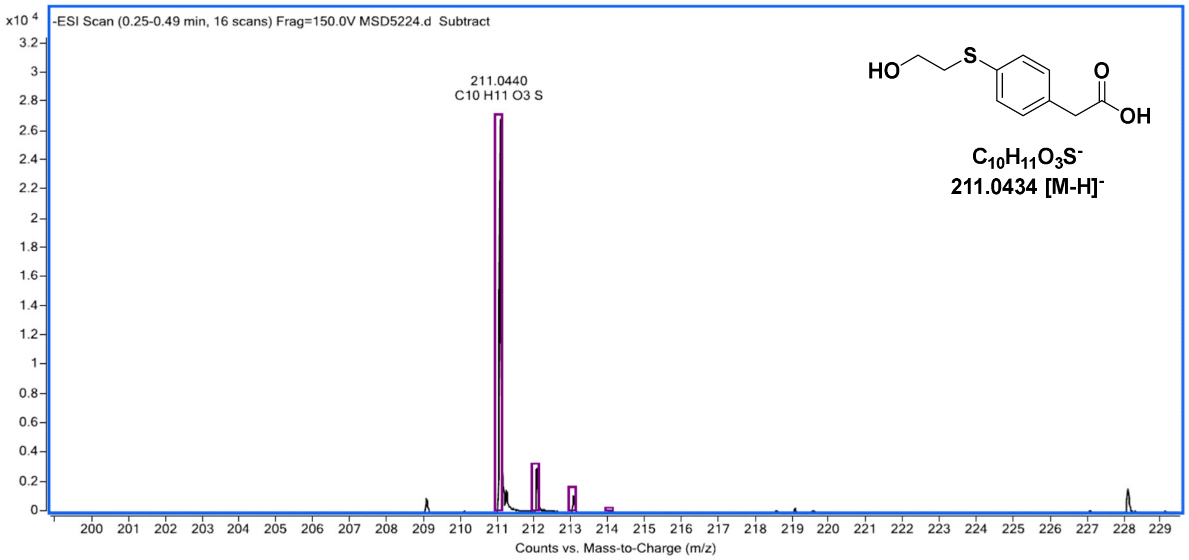

Figure S54: HRMS for 2-(4-((2-hydroxyethyl) thio) phenyl acetic acid

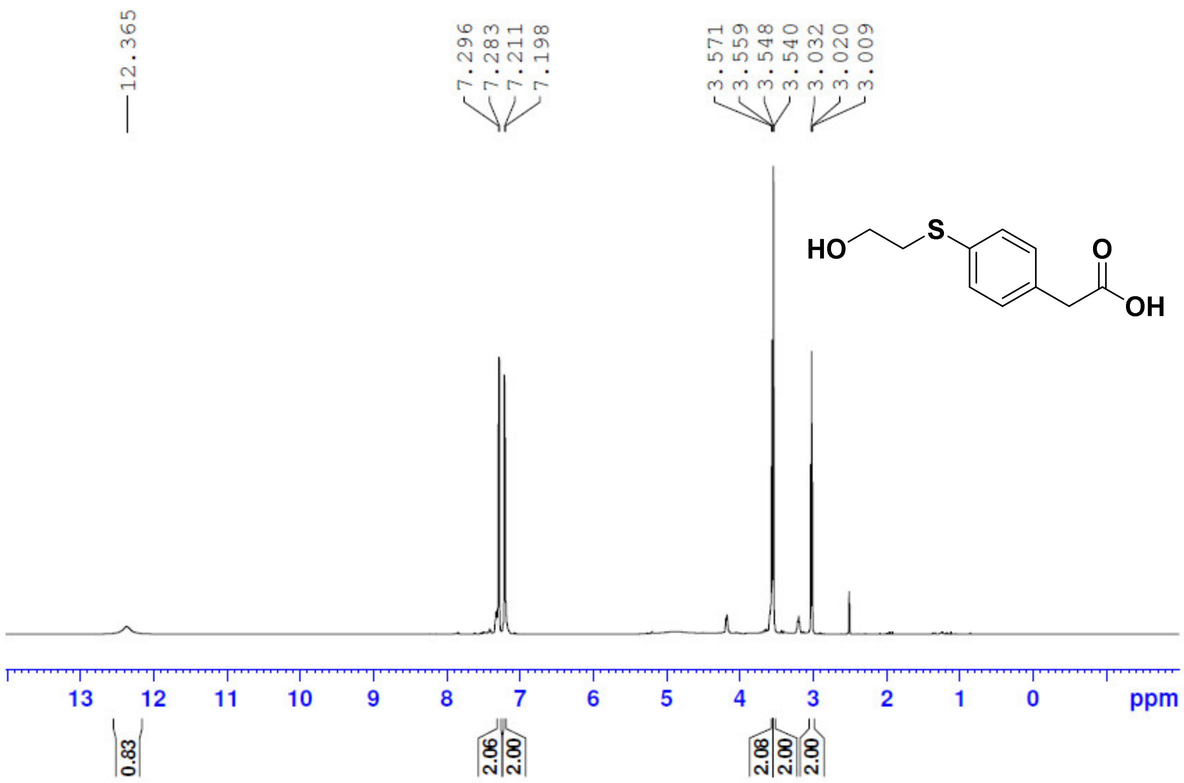

Figure S55: ^{1}H NMR for 2-(4-((2-hydroxyethyl) thio) phenyl acetic acid.

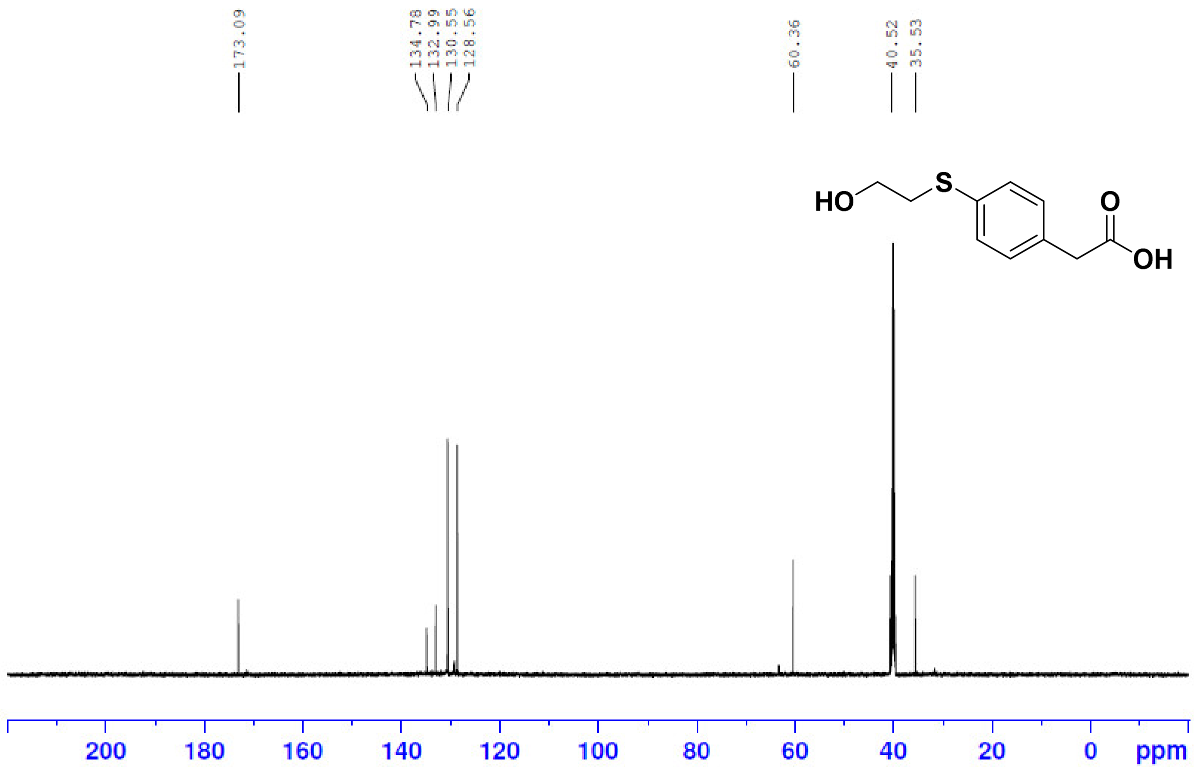

Figure S56: ^{13}C NMR for 2-(4-((2-hydroxyethyl) thio) phenyl acetic acid.
